# Synthesis and Evaluation of Novel Ligustrazine Derivatives as Multi-Targeted Inhibitors for the Treatment of Alzheimer’s Disease

**DOI:** 10.3390/molecules23102540

**Published:** 2018-10-05

**Authors:** Wenhao Wu, Xintong Liang, Guoquan Xie, Langdi Chen, Weixiong Liu, Guolin Luo, Peiquan Zhang, Lihong Yu, Xuehua Zheng, Hong Ji, Chao Zhang, Wei Yi

**Affiliations:** Key Laboratory of Molecular Target & Clinical Pharmacology, School of Pharmaceutical Sciences, Guangzhou Medical University, Guangzhou 511436, Guangdong, China; wenhao_wu@gzhmu.edu.cn (W.W.); kilyte18@163.com (X.L.); guoquanxie@163.com (G.X.); chenlangdi427@gmail.com (L.C.); 13760781141@139.com (W.L.); 13570203692@163.com (G.L.); pqzhang@gzhmu.edu.cn (P.Z.); ylh84@126.com (L.Y.); zhengxh23@gzhmu.edu.cn (X.Z.); dljih@126.com (H.J.)

**Keywords:** ligustrazine, acetylcholinesterase, self-induced Aβ aggregation, multi-targeted inhibitors, Alzheimer’s disease

## Abstract

A series of novel ligustrazine derivatives **8a**–**r** were designed, synthesized, and evaluated as multi-targeted inhibitors for anti-Alzheimer’s disease (AD) drug discovery. The results showed that most of them exhibited a potent ability to inhibit both ChEs, with a high selectivity towards AChE. In particular, compounds **8q** and **8r** had the greatest inhibitory abilities for AChE, with IC_50_ values of 1.39 and 0.25 nM, respectively, and the highest selectivity towards AChE (for **8q**, IC_50_ BuChE/IC_50_ AChE = 2.91 × 10^6^; for **8r**, IC_50_ BuChE/IC_50_ AChE = 1.32 × 10^7^). Of note, **8q** and **8r** also presented potent inhibitory activities against Aβ aggregation, with IC_50_ values of 17.36 µM and 49.14 µM, respectively. Further cellular experiments demonstrated that the potent compounds **8q** and **8r** had no obvious cytotoxicity in either HepG2 cells or SH-SY5Y cells, even at a high concentration of 500 μM. Besides, a combined Lineweaver-Burk plot and molecular docking study revealed that these compounds might act as mixed-type inhibitors to exhibit such effects via selectively targeting both the catalytic active site (CAS) and the peripheral anionic site (PAS) of AChEs. Taken together, these results suggested that further development of these compounds should be of great interest.

## 1. Introduction

Alzheimer’s disease (AD) is a progressive neurodegenerative brain disorder that is manifested as dementia, cognitive impairment, memory loss, severe behavioral abnormalities, and ultimately death [1,2,3,4]. To date, AD is thought to be a complex, multifactorial syndrome, with many related molecular lesions contributing to its pathogenesis. Based on the existing hypothesis, AD is characterized as amyloid plaques, neurofibrillary tangles, inflammatory intermediates, and reactive oxygen species (ROS), and imposes neuronal death via a complex array of networked pathways [5].

According to the cholinergic hypothesis, the cognitive and memory deterioration of AD is due to a loss of cholinergic function in the central nervous system. Acetylcholinesterase (AChE) and butyrylcholinesterase (BuChE) are two major cholinesterases (ChE) involved in the hydrolysis and regulation of choline in vertebrates. Indeed, current treatment of AD mainly focuses on the inhibition of AChE activity in order to rectify the deficiency of cerebral acetylcholine [6,7]. However, the role of BuChE in the progression of AD has recently been proved. For instance, BuChE inhibitors could recover cholinergic activity through restoring the AChE/BuChE activity ratios, as shown in a healthy brain [8], and BuChE may play a role in AD plaque due to its contribution to a subpopulation of Aβ plaques maturation by immunostaining analysis of AD brain tissues [9]. Nevertheless, many studies have showed that both ChEs could facilitate amyloid fibril formation to yield stable ChE-Aβ complexes, which are more toxic than a single Aβ peptide [10,11]. The complex of AChE, through interaction with Aβ, promotes amyloid fibril formation through a spanning hydrophobic sequence exposed on the surface of AChE, which is close to the peripheral anionic binding site (PAS) and interacts with liposomes. Besides, an AChE-derived 35-residue peptide corresponding to the above hydrophobic sequence is incorporated into the growing Aβ-fibrils. Therefore, drugs capable of inhibiting ChE might have beneficial therapy effects on the cognitive, functional, and behavioural symptoms of AD.

As a result, the development of the inhibitor targeting ChE, especially for AChE, has been an intensive research focus and a large number of AChE inhibitors have been reported thus far. Among them, those named “dual-site” AChE inhibitors occupy a particularly prominent position for the treatment of AD since they can interact with both the CAS and the PAS of AChE, leading to the potent activation of the cholinergic system, as well as the efficient inhibition of AChE-promoted Aβ production and aggregation [12]. 

On the other hand, the multi-target approach has been proposed as particularly suitable for combating the heterogeneity and the multifactorial nature of AD [13]. Compared to single-targeted drugs, several pieces of literature have disclosed that, the candidate compound, possessing two or more distinct pharmacological properties closely related to the neurodegenerative process, is more effective in AD therapy [14,15,16,17,18,19]. Building on this strong foundation, ever-increasing efforts toward multi-target drug discovery are being made in the field for the treatment of AD [20].

Ligustrazine (tetramethylpyrazine (TMP), **1**), one of the major efficacious components of the Chinese traditional medicine herb chuanxiong (*Ligusticum wallichii*), is widely used in China as a novel calcium channel antagonist for the treatment of coronary atherosclerotic cardiovascular disease and ischemic cerebrocardiac vascular disease [21]. TMP is also used with a combination of Rhizoma Curcumae Longae and Calculus bovis to treat cancer [22]. A preliminary pharmacokinetic investigation revealed that TMP could be rapidly absorbed into the blood and then crosses the blood-brain and blood-labyrinth barriers [23]. Recently, it was also reported that TMP could inhibit AChE, self-induced Aβ aggregation, by acting as the potential antioxidant [24,25,26]. 

Motivated by the aforementioned information and in continuation of our interest in developing multi-target inhibitors as potent candidates for the treatment of AD [27], we herein disclose the design, synthesis, and biological evaluation of a series of novel ligustrazine-based derivatives as multi-targeted inhibitors against AChE, BChE, and Aβ aggregation for potent treatment of AD. Furthermore, the structure–activity relationships (SARs) are discussed and the inhibition mechanism, the inhibitory kinetics, and the cytotoxicity of selected compounds are studied systematically. Taken together, our present results demonstrate that such compounds can serve as a promising candidate for the treatment of AD and establish a rational foundation for future design of new pharmacological agents as multi-target inhibitors of AChE, BChE, and Aβ aggregation.

## 2. Results and Discussion

### 2.1. Chemistry

**Scheme 1:**
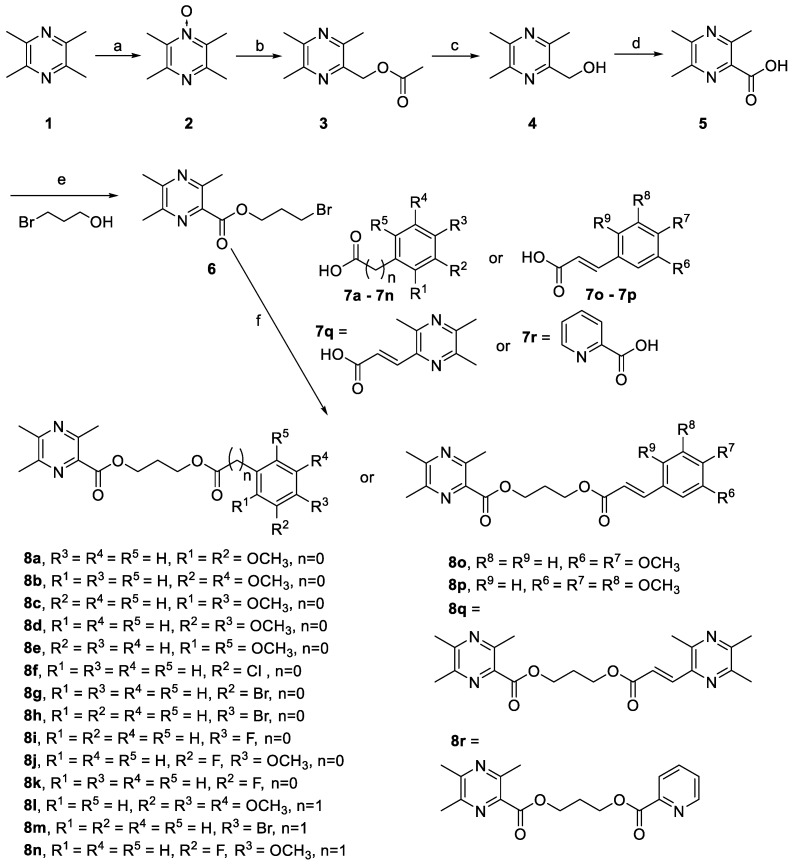
Synthesis of novel ligustrazine hybrids. Reagents and conditions: (a) 30% H_2_O_2_, HOAc, 90 °C, 4 h; (b) Ac_2_O, reflux, 105 °C, 2.5 h; (c) NaOH, THF: MeOH: H_2_O, r.t., 1 h; (d) KMnO_4_, H_2_O, 50 °C, 12 h; (e) EDC^.^HCl, DMAP, CH_2_Cl_2_, r.t., 24 h; (f) anhydrous K_2_CO_3_, DMF, 30 °C, 12 h.

The ligustrazine hybrids were conveniently synthesized according to the synthetic routes shown in Scheme 1. First, compound **5** was prepared based on a previous reported method [24]. Then, it was treated with 3-bromopropan-1-ol in the presence of EDC·HCl and DMAP to yield 3-bromopropyl 3,5,6-trimethylpyrazine-2-carboxylate **6** in a yield of 73%. Finally, intermediate **6** was reacted with the corresponding carboxylic acid derivatives **7a**–**r** in the presence of DMF and anhydrous K_2_CO_3_ to afford the desired products **8a**–**r**. The structures of synthesized compounds were confirmed by ^1^H and ^13^C-NMR and HRMS. It should be noted that 3-(3,5,6-trimethylpyrazin-2-yl)acrylic acid (**7q**) was smoothly synthesized according to the literature, with a total yield of 60% [28]. 

### 2.2. In Vitro Inhibition Studies on AChE, BuChE and Aβ (1-42) Self-Induced Aggregation.

To determine the potential of the target compounds **8a**–**r** for the treatment of AD, their AChE (from electric eel) and BuChE (from equine serum) inhibitory activities were evaluated by using the method of Ellman [29], in which tacrine and galanthamine were employed as the reference compounds. The IC_50_ values of all compounds for ChEs (AChE and BuChE) and the affinity ratios were summarized as shown in Table 1. The results demonstrated that most of the tested compounds had a potent capability to inhibit AChE, even with an IC_50_ value at the nanomolar level. In sharp contrast, these compounds showed relatively weak inhibitory activities towards BuChE (with an IC_50_ value in the millimol grade). Obviously, these compounds displayed a good selectivity for AChE over BuChE, and the ratios of IC_50_ BuChE/IC_50_ AChE affinity values ranged from 1.14 × 10^2^ to 1.32 × 10^7^.

As shown in Table 1, the reference compound-tacrine had a potent inhibitory activity, with an IC_50_ value of 73.36 nM, which was in good agreement with previous reports [27,30]. Interestingly, among these, compound **8r**, bearing a picolinic acid group, showed the most potent inhibition for AChE, with an IC_50_ value of 0.25 nM, and the potency was 293-times stronger than tacrine. However, its IC_50_ value against BuChE was 3.30 mM, which was much weaker than AChE. Moreover, the greatest selectivity index was also obtained for this compound, with an IC_50_ BuChE/IC_50_ AChE value of 1.32 × 10^7^. It indicated that compound **8r** had an exclusive selectivity for AChE over BuChE. Furthermore, an SAR analysis suggested that the electron density of the benzene ring moiety in the product played a significant role in determining the inhibitory activity of AChE, and in general, the compounds bearing the electron-donating substituent exhibited more potent AChE inhibitory activity than those bearing the electron-withdrawing group. For instance, compounds **8a**–**8e,** in which the two methoxyl substituents were installed in different positions of the benzene ring, showed strong AChE inhibitory activities, with ranges of IC_50_ values from 4.12 to 387.9 nM. However, the homogeneous class of compounds **8g**–**h** and **8m**, bearing a bromo substituent in the benzene ring, exhibited relatively low AChE inhibitory activities compared with **8a**–**e**.

Moreover, compounds **8l**–**n** exhibited AChE inhibitory effects, with IC_50_ values that ranged from 303.4 µM to 7.16 mM, which was less active than **8a**–**k** (from 4.12 nM to 993.39 µM), suggesting that the introduction of the methylene part between the ester group and the benzene ring was harmful to AChE inhibitory activity. Interestingly, the introduction of the conjugated vinyl group into **8d** that gave the compound **8o**, demonstrated potent AChE inhibitory effects, with an IC_50_ value of 3.24 nM. A similar conclusion was observed in comparison with **8l** and **8p**. The results revealed that the introduction of a proper and relatively rigid group between the ester group and the benzene ring was beneficial to AChE inhibitory activity. Of note, replacement of the benzene ring moiety with the *N*-heterocyclic ring, such as pyrazine or pyridine, led to a dramatic increase in inhibitory activity, since the obtained compounds **8q** and **8r** were found to be the most potent inhibitors of AChE, with excellent selectivity towards AChE (for **8q**, IC_50_ = 1.39 nM, IC_50_ BuChE/IC_50_ AChE = 1.32 × 10^7^; for **8r**, IC_50_ = 0.25 nM, IC_50_ BuChE/IC_50_ AChE = 2.91 × 10^6^), indicating that the introduction of the nitrogen atom contributed to AChE inhibitory activities, probably due to the additional affinity with the active pocket of AChE.

Inspired by the above results, these synthesized compounds were further tested for their abilities to inhibit self-mediated aggregation of Aβ (1-42) by using a thioflavin-T fluorescence method [29] and employing the well-known tacrine as a standard reference. As summarized in Table 1, the results showed that most of the ligustrazine derivatives apparently prevented self-mediated Aβ aggregation. In particular, compounds **8b** (IC_50_ = 3.66 µM), **8j** (IC_50_ = 7.12 µM), and **8k** (IC_50_ = 5.10 µM) showed higher potency than that of the reference compound tacrine (12.21 µM). Gratefully, the two best potent AChE inhibitors **8q** and **8r** also possessed acceptable inhibitory activities against self-induced Aβ (1-42) aggregation, with IC_50_ values of 17.36 µM and 49.14 µM, respectively. Taken together, the data from the ChEs and Aβ aggregation test revealed that these developed compounds might act as novel multi-targeted “hits” for potent anti-AD drug discovery. Thus, further development of such compounds should be of great interest.

### 2.3. Inhibition of Aβ (1-42) Fibril Formation Monitored by Transmission Electron Microscopy (TEM)

**Figure 1:**
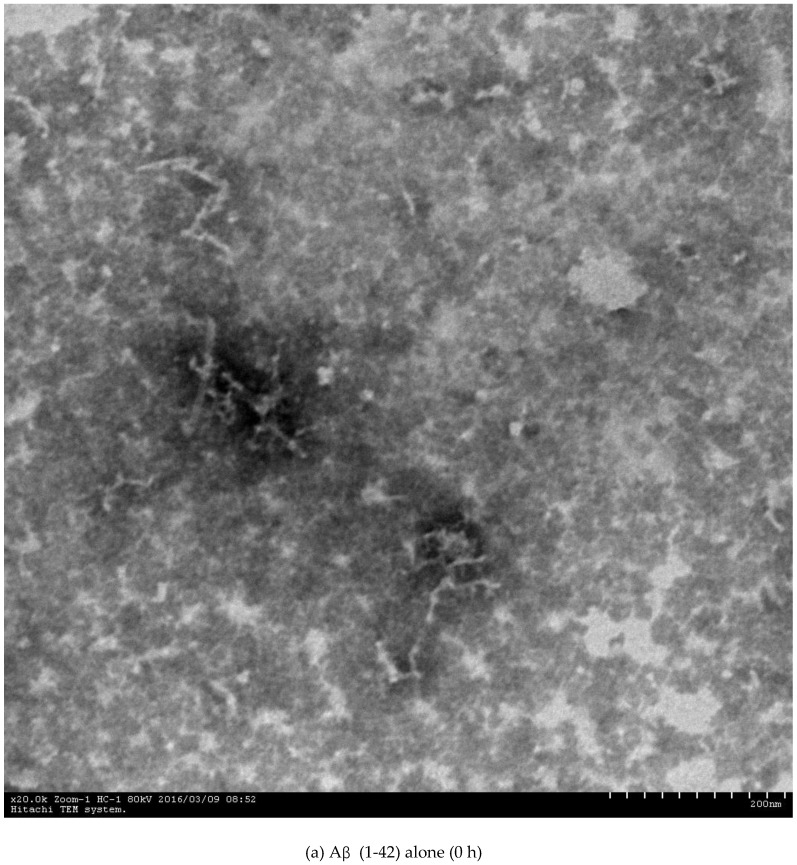

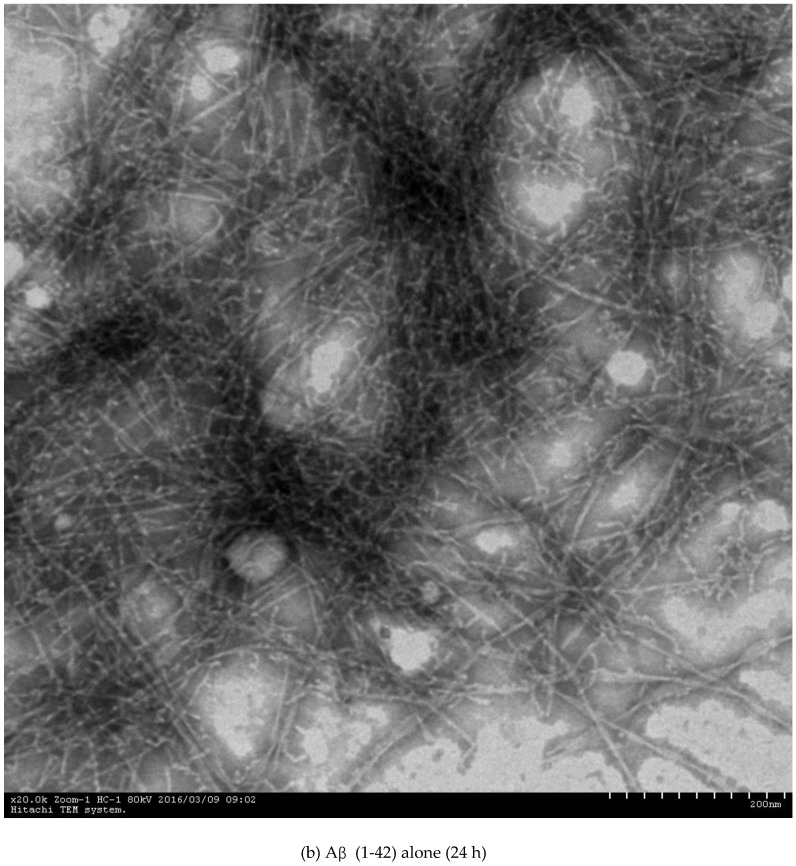

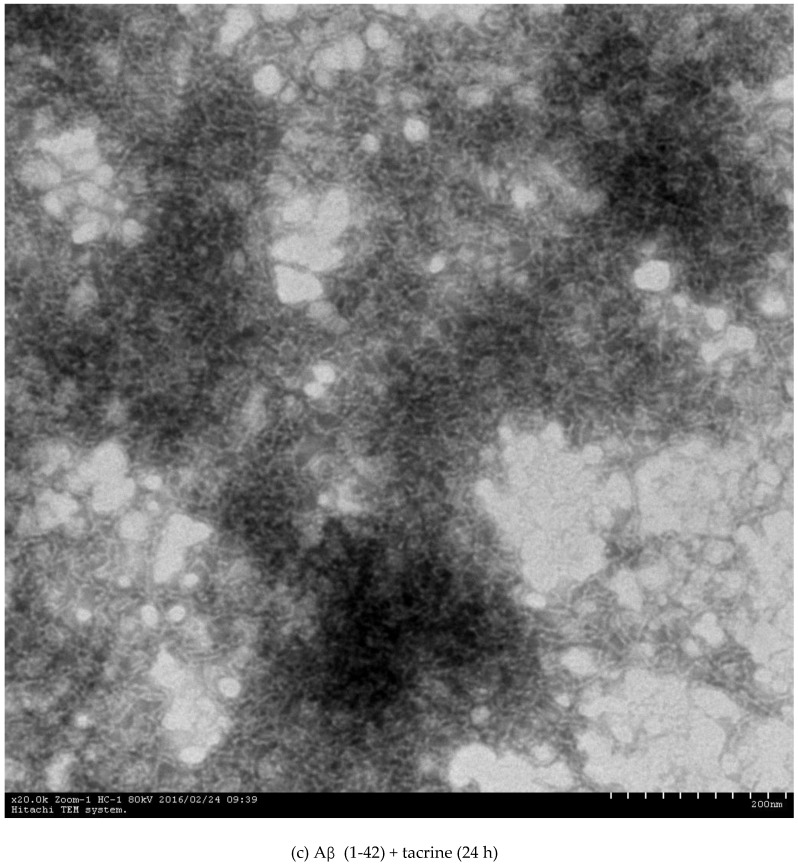

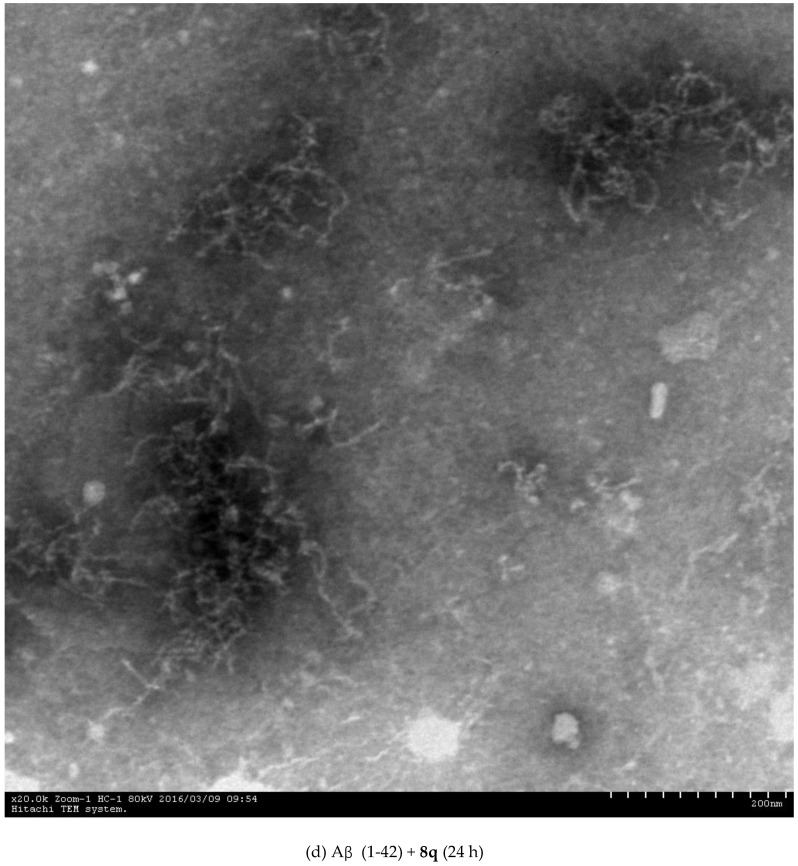
TEM image analysis of Aβ (1-42) aggregation. (**a**) Aβ (1-42) alone (20 µM) was incubated at 37 °C for 0 h; (**b**) Aβ (1-42) alone (20 µM) was incubated at 37 °C for 24 h; (**c**)Aβ (1-42) (20 µM) and tacrine (50 µM) were incubated at 37 °C for 24 h; (**d**) Aβ (1-42) (20 µM) and **8q** (50 µM) were incubated at 37 °C for 24 h.

To further confirm the ability of these compounds in inhibiting Aβ (1-42) aggregation, the inhibitory activity of the selected compound **8q** was monitored by using TEM [31]. As shown in Figure 1, after 24 h of incubation at 37 °C, Aβ (1-42) alone aggregated into well-defined Aβ fibrils and amorphous deposits were observed (Figure 1a,b). In sharp contrast, no obvious Aβ fibril was detected in the presence of either tacrine or **8q** (Figure 1c,d) under identical conditions. Moreover, compound **8q** could obviously alleviate the formation of Aβ amorphous deposits compared with tacrine. These results were in agreement with the data from the thioflavin-T fluorescence experiment, also suggesting that **8q** can serve as a promising candidate for the treatment of AD. 

### 2.4. Kinetic Characterization of AChE Inhibition

**Figure 2:**
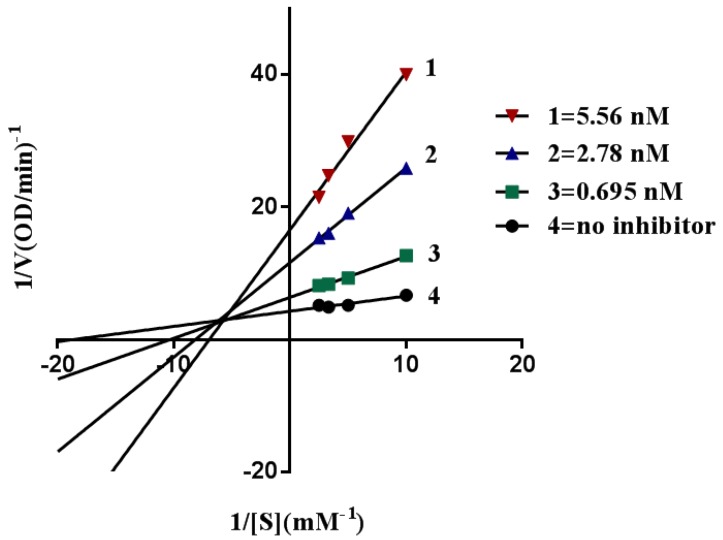
Determination of the inhibitory type of selected compound **8q** on AChE. Merged Lineweaver-Burk reciprocal plots of AChE initial velocity with increasing substrate concentrations (0.1–0.4 mM) in the absence or presence of **8q**. Lines were derived from a weighted least-squares analysis of data points.

Encouraged by the above results, we further selected the most potent AChE inhibitor **8q** for kinetic analysis to uncover the inhibition mechanism by using the graphical analysis of the reciprocal Lineweaver–Burk plot. As demonstrated in Figure 2, the results showed that the plots of 1/V versus 1/[S] gave a family of straight lines, with different slopes that intersected one another in the second quadrant. With the increase of the concentration of **8q**, the values of Vmax descended, but the values of Km kept increasing, indicating that it was a mixed-type inhibitor of AChE. 

### 2.5. Molecular Modeling Study of AChE

**Figure 3:**
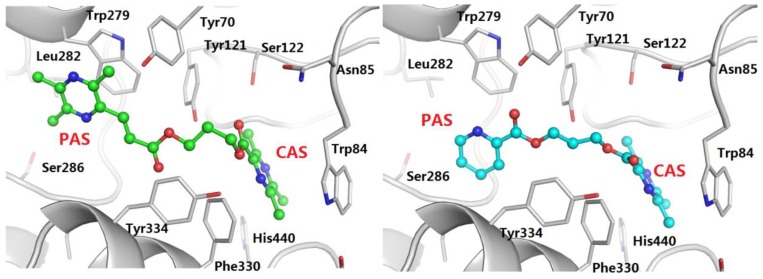
Binding modes of **8q** (green carbon) and **8r** (cyan carbon) obtained from molecular docking. Surrounding residues are depicted in grey (carbon), red (oxygen), and blue (nitrogen).

To gain further insight into the molecular basis, the most active compounds **8q** and **8r** were selected to dock into the active site of TcAChE. It is well-known that, in previous crystallographic studies, significant conformation changes were observed in the active site of TcAChE [31,32,33,34,35]. For example, bis-tacrine derivatives with a 7-carbon spacer (PDB code 2ckm) induced the rotation of side chains of Tyr70 and Trp279 to promote the sandwich interaction with the active PAS site in the pocket of AChE, while their analogues with a 5-carbon spacer (PDB code 2cmf) caused the structural alteration of Trp279-Ser291’s loop in the CAS site [32]. Based on this information, we herein used 2ckm and 2cmf as molecular modeling for **8q** containing a 7-carbon spacer and **8r** with a 5-carbon spacer, respectively. The results showed that no large-scale structural displacement occurred in **8q** or **8r** binding to AChE, mainly due to their small molecular size compared with tacrine derivatives. Alternatively, the crystal structure of the HupA-TcAChE complex (PDB code 1vot), where most residues were retained in the native conformations, was chosen as the molecular docking model [33]. Gratefully, re-docking of the original ligand into the active site gave an acceptable RMSD difference of 0.5384, which verified the reliability of our strategy. Figure 3 shows that both **8q** and **8r** spanned the active gorge with an extended conformation. The proximal pyrazine ring was bound in the CAS at the bottom, stacking against the side chain of Trp84, while the distal pyrazine/pyridine ring was present in the PAS near the mouth, forming a π-π interaction with Trp279. Moreover, some hydrophobic or van der Waals interactions might occur between **8q** or **8r** and the surrounding residues, such as Tyr70, Asp72, Asn85, Gly119, Tyr121, Ser122, Gly123, Tyr130, Leu282, Ser286, Ile287, Phe288, Phe290, Phe330, Phe331, Tyr334, Gly335, Trp432, His440, and Tyr442. Such interactions all contributed to their potent affinities with AChE. It should be noted that, owing to the fact that BuChE did not have the PAS [36], these compounds could not form effective binding interactions with the active pocket of BuChE, thereby giving a specific selectivity towards AChE. Finally, the result also revealed that compound **8q** could bind to both the CAS and the PAS of AChE.

### 2.6. Cytotoxicity Assay for ***8q*** and ***8r***

One of the best documented side-effects of the drug discovery and development of AD is cytotoxicity [37,38,39]. To probe whether such a type of target compounds involves cytotoxic effects, we respectively treated HepG2 cells and SH-SY5Y cells with several concentrations of the corresponding compound. Cell viability was determined by a CCK-8 assay. As reported, the referenced compound tacrine showed dose-dependent cytotoxicity (Table 2 and Table 3). Excitedly, our potent “hits” **8q** and **8r** displayed no obvious cytotoxicity in the two cell lines, even at a high concentration of 500 μM, suggesting that potent compounds **8q** and **8r** have a potent safe window for the next stage of the development of anti-AD drug discovery.

## 3. Materials and Methods 

### 3.1. Chemistry

All commercially available compounds and dried solvents were used without further purification. The NMR (^1^H and ^13^C) spectra were recorded on a Bruker Avnace III 400 spectrometer (Bruker Avnace, Billerica, MA, USA) or Varian Mercury–Plus 300 spectrometer (Palo Alto, CA, USA). Chemical shifts (δ) were referenced to TMS as an internal standard. Coupling constants are given in Hz. MS spectra were obtained on an Agilent 6330 Ion Trap Mass Spectrometer (Palo Alto, CA, USA). HRMS spectra were recorded on a Shimazu LCMS-IT-TOF mass spectrometer (Kyoto, Japan). Microscopy images were captured on a HITACHI H-7650 transmission electron microscope (Tokyo, Japan). TLC was monitored by precoated silica gel GF254 plates purchased from Merck& Co., (Darmstadt, Germany) and the spots were detected through a UV lamp (Yuhua Co. Ltd., Guangzhou, China) at 254 nm. Flash column chromatography was performed with silica gel (200–300 mesh) purchased from Qingdao Haiyang Chemical Co. Ltd., Qingdao, China.

### 3.2. Synthesis of Intermediate ***6***

3-Bromopropan-1-ol was added dropwise to an ice-cold solution of **5** (20.0 mmol), DMAP (10 mg), and EDC·HCl (52 mmol) in CH_2_Cl_2_ (20 mL). After the addition had been completed, the mixture was allowed to adjust to room temperature and was stirred for 24 h. When the reaction had been monitored by TLC, the solvent was removed in vacuum. The obtained brown residue was dissolved by adding distilled water, extracted with EtOAc three times (50 mL × 3), and washed with water. The organic layer was washed with brine dried over anhydrous Na_2_SO_4_. It was concentrated under reduced pressure to give compound **6** (2.08 g) as tan jelly without further purification. The yield was 73%.

### 3.3. General Procedures for the Preparation of ***8a**–**r***

To a solution of **7** (1.22 mmol) in DMF (2 mL), anhydrous K_2_CO_3_ (1.62 mmol) was first added, **6** (0.81 mmol) in DMF (2 mL) was then added dropwise, and the reaction mixture was stirred at 30 °C for 3 h. When the reaction was completed, the mixture was diluted with water (20 mL) and extracted with EtOAc (25 mL × 3). The organic layer was washed with brine, dried over anhydrous Na_2_SO_4_, and concentrated under reduced pressure. The obtained residue was purified by silica gel chromatography to afford the desire product.

*3-[(2,3-Dimethoxybenzoyl)oxy]propyl 3,5,6-trimethylpyrazine-2-carboxylate* (**8a**). Compound **6** was treated with 2,3-dimethoxybenzoic acid (**7a**) according to general procedure to give the desired product **8a** as a white solid. Yield 48%, ^1^H-NMR (CDCl_3_, 300 MHz) δ (ppm): 2.29 (p, *J* = 6.3 Hz, 2H), 2.56 (s, 6H), 2.74 (s, 3H), 3.90 (d, *J* = 8.5 Hz, 6H), 4.48 (t, *J* = 6.2 Hz, 2H), 4.58 (t, *J* = 6.5 Hz, 2H), 7.06 (d, *J* = 3.9 Hz, 2H), 7.38–7.27 (m, 1H).^13^C-NMR (CDCl_3_, 75 MHz) δ (ppm): 21.55, 22.17, 22.57, 28.07, 56.05, 61.58 (d, *J* = 14.0 Hz), 62.56, 115.86, 122.19, 123.80, 125.95, 139.40, 149.18 (d, *J* = 16.3 Hz), 151.13, 153.52, 154.50, 165.91, 166.18. ESI-HRMS Calcd. for C_20_H_24_N_2_O_6_ [M + Na]^+^: 411.1527; found: 411.1532.

*3-[(3,5-Dimethoxybenzoyl)oxy]propyl 3,5,6-trimethylpyrazine-2-carboxylate* (**8b**). Compound **6** was treated with 3,5-dimethoxybenzoic acid (**7b**) according to general procedure to give the desired product **8b** as a white solid. Yield 46%, ^1^H-NMR (CDCl_3_, 300 MHz) δ (ppm): 2.30 (p, *J* = 6.3 Hz, 2H), 2.54(s, 3H), 2.56 (s, 3H), 2.73 (s, 3H), 3.82 (s, 6H), 4.49 (t, *J* = 6.2 Hz, 1H), 4.58 (t, *J* = 6.4 Hz, 1H), 6.63 (t, *J* = 2.4 Hz, 1H), 7.15 (d, *J* = 2.4 Hz, 2H).^13^C-NMR (CDCl_3_, 75 MHz) δ (ppm): 21.53, 22.15, 22.57, 28.10, 55.54, 61.87, 62.60, 105.61, 107.14(d, *J* = 4.4 Hz), 131.87, 139.29, 149.26, 151.19,154.52, 160.58, 165.86, 166.19. ESI-HRMS Calcd. for C_20_H_24_N_2_O_6_ [M + Na]^+^: 411.1527; found: 411.1535.

*3-[(2,4-Dimethoxybenzoyl)oxy]propyl 3,5,6-trimethylpyrazine-2-carboxylate* (**8c**). Compound **6** was treated with 2,4-dimethoxybenzoic acid (**7c**) according to general procedure to give the desired product **8c** as a white solid. Yield 48%, ^1^H-NMR (CDCl_3_, 300 MHz) δ (ppm):2.26 (m, 2H), 2.54 (s, 6H), 2.71 (s, 3H), 3.86 (s, 3H), 3.89(s, 3H), 4.45 (t, *J* = 6.2 Hz, 2H), 4.55 (t, *J* = 6.5 Hz, 2H), 7.03 (s, 1H), 7.04 (d, *J* = 1.7 Hz, 1H), 7.28 (dd, *J* = 5.5, 3.9 Hz, 1H). ^13^C-NMR (CDCl_3_, 75 MHz) δ (ppm): 21.53, 22.14, 22.54, 28.05, 56.02, 61.55, 62.53, 115.87, 122.20, 123.78, 125.92, 139.37, 149.15, 151.09, 153.50, 154.47, 165.88, 166.15. ESI-HRMS Calcd. for C_20_H_24_N_2_O_6_ [M + Na]^+^: 411.1527; found: 411.1538.

*3-[(3,4-Dimethoxybenzoyl)oxy]propyl 3,5,6-trimethylpyrazine-2-carboxylate* (**8d**). Compound **6** was treated with 3,4-dimethoxybenzoic acid (**7d**) according to general procedure to give the desired product **8d** as a white solid. Yield 41%, ^1^H-NMR (*d*_6_-DMSO, 400 MHz) δ (ppm): 2.18 (m, 2H), 2.43 (s, 3H), 2.48 (s, 3H), 2.57 (s, 3H), 3.77 (s, 3H), 3.83 (s, 3H), 4.38 (t, *J* = 6.1 Hz, 2H), 4.47 (t, *J* = 6.2 Hz, 2H), 7.00 (d, *J* = 8.5 Hz, 1H), 7.38 (d, *J* = 2.0 Hz, 1H), 7.54 (dd, *J* = 8.4, 2.0 Hz, 1H). ^13^C-NMR (*d*_6_-DMSO, 101 MHz) δ (ppm): 21.42, 22.15, 22.31, 28.08, 55.90, 56.15, 62.11, 62.95, 111.40, 112.02, 122.25, 123.55, 139.51, 148.76, 149.47, 150.16, 153.34, 154.77, 165.89, 165.92. ESI-HRMS Calcd. for C_20_H_24_N_2_O_6_ [M + Na]^+^: 411.1527; found: 411.1528.

*3-[(2,6-Dimethoxybenzoyl)oxy]propyl 3,5,6-trimethylpyrazine-2-carboxylate* (**8e**). Compound **6** was treated with 2,6-dimethoxybenzoic acid (**7e**) according to general procedure to give the desired product **8e** as a white solid. Yield 51%, ^1^H-NMR (CDCl_3_, 300 MHz) δ (ppm): 2.25 (m, 2H), 2.56 (s, 6H), 2.73 (s, 3H), 3.81 (s, 6H), 4.49 (t, *J* = 6.1 Hz, 2H), 4.55 (t, *J* = 6.5 Hz, 2H), 6.54 (d, *J* = 8.4 Hz, 2H), 7.27 (t, *J* = 8.4 Hz, 1H). ^13^C-NMR (CDCl_3_, 75 MHz) δ21.54, 22.14, 22.55, 27.99, 55.94, 61.59, 62.53, 103.83, 112.82, 131.15, 139.59, 149.27, 150.93, 154.39, 157.33, 165.99, 166.42. ESI-HRMS Calcd. for C_20_H_24_N_2_O_6_ [M + Na]^+^: 411.1527; found: 411.1526.

*3-[(3-Chlorobenzoyl)oxy]propyl 3,5,6-trimethylpyrazine-2-carboxylate* (**8f**). Compound **6** was treated with 3-chlorobenzoic acid (**7f**) according to general procedure to give the desired product **8f** as a white solid. Yield 51%, ^1^H-NMR (CDCl_3_, 300 MHz) δ (ppm): 2.30 (m, 2H), 2.53 (s, 3H), 2.55 (s, 3H), 2.72 (s, 3H), 4.49 (t, *J* = 6.2 Hz, 2H), 4.57 (t, *J* = 6.4 Hz, 2H), 7.34 (t, *J* = 7.9 Hz, 1H),7.50 (ddd, *J* = 8.0, 2.2, 1.1 Hz, 1H), 7.88 (dt, *J* = 7.7, 1.3 Hz, 1H), 7.95 (t, *J* = 1.8 Hz, 1H). ^13^C-NMR (CDCl_3_, 75 MHz) δ (ppm): 21.54, 22.16, 22.57, 28.04, 62.17, 62.55, 127.66, 129.61 (d, *J* = 7.1 Hz), 131.73, 132.96, 134.44, 139.21, 149.27, 151.21, 154.56, 165.19, 165.84. ESI-HRMS Calcd. for C_18_H_19_N_2_O_4_Cl [M + Na]^+^: 385.0926; found: 385.0936.

*3-[(3-Bromobenzoyl)oxy]propyl 3,5,6-trimethylpyrazine-2-carboxylate* (**8g**). Compound **6** was treated with 3-bromobenzoic acid **(7g**) according to general procedure to give the desired product **8g** as a white solid. Yield 42%, ^1^H-NMR (*d*_6_-DMSO, 400 MHz) δ (ppm): 2.19 (m, 2H), 2.41 (s, 3H), 2.47 (s, 3H), 2.56 (s, 3H), 4.43 (t, *J* = 5.9 Hz, 2H), 4.48 (t, *J* = 6.0 Hz, 2H), 7.43 (t, *J* = 7.3 Hz, 1H), 7.81 (d, *J* = 7.9 Hz, 1H), 7.94 (d, *J* = 7.6 Hz, 1H), 8.04 (s, 1H). ^13^C-NMR (*d*_6_-DMSO, 101 MHz) δ (ppm): 165.83, 164.89, 150.22, 139.35, 138.38, 136.39, 132.50, 131.92, 131.45, 131.26, 128.55, 122.25, 63.02, 57.68, 31.86, 27.88, 22.29, 22.14, 21.40. ESI-HRMS Calcd. for C_18_H_19_N_2_O_4_Br [M + H]^+^: 407.0601; found: 407.0605.

*3-[(4-Bromobenzoyl)oxy]propyl 3,5,6-trimethylpyrazine-2-carboxylate* (**8h**). Compound **6** was treated with 4-bromobenzoic acid (**7h**) according to general procedure to give the desired product **8h** as a white solid. Yield 42%, ^1^H-NMR (*d*_6_-DMSO, 400 MHz) δ (ppm): 2.19 (m, 2H), 2.43 (s, 3H), 2.49 (s, 3H), 2.57 (s, 3H), 4.42 (t, *J* = 6.0 Hz, 2H), 4.47 (t, *J* = 6.2 Hz, 2H), 7.66 (d, *J* = 8.5 Hz, 2H), 7.81 (d, *J* = 8.5 Hz, 2H). ^13^C-NMR (*d*_6_-DMSO, 101 MHz) δ (ppm): 21.43, 22.21, 22.32, 27.92, 31.93, 57.68, 62.80, 63.01, 127.74, 129.26, 131.48, 132.18, 139.45, 149.48, 154.81, 165.46, 165.90. ESI-HRMS Calcd. for C_18_H_19_N_2_O_4_Br [M + H]^+^: 407.0601; found: 407.0616.

*3-[(4-Fluorobenzoyl)oxy]propyl 3,5,6-trimethylpyrazine-2-carboxylate* (**8i**). Compound **6** was treated with 4-fluorobenzoic acid (**7i**) according to general procedure to give the desired product **8i** as a white solid. Yield 42%, ^1^H-NMR (*d*_6_-DMSO, 400 MHz) δ (ppm):2.19 (m, 2H), 2.44 (s, 3H), 2.49 (s, 3H), 2.57 (s, 3H), 4.41 (t, *J* = 6.1 Hz, 2H), 4.48 (t, *J* = 6.2 Hz, 2H), 7.29 (t, *J* = 8.8 Hz, 2H), 7.98 (dd, *J* = 8.8, 5.6 Hz, 1H). ^13^C-NMR (*d*_6_-DMSO, 101 MHz) δ (ppm): 21.44, 22.17, 22.30, 27.97, 31.98, 57.70, 62.56, 62.92, 116.08, 116.30, 132.39, 132.48, 139.48, 149.52, 150.20, 154.82, 165.21, 165.92. ESI-HRMS Calcd. for C_18_H_19_N_2_O_4_F [M + H]^+^: 347.1402; found: 347.1411.

*3-[(3-Fluoro-4-methoxybenzoyl)oxy]propyl 3,5,6-trimethylpyrazine-2-carboxylate* (**8j**). Compound **6** was treated with 3-fluoro-4-methoxybenzoic acid (**7j**) according to general procedure to give the desired product **8j** as a white solid. Yield 43%, ^1^H-NMR (*d*_6_-DMSO, 400 MHz) δ (ppm): 2.18 (m, 2H), 2.44 (s, 3H), 2.48 (s, 3H), 2.57 (s, 3H), 3.91 (s, 3H), 4.39 (t, *J* = 6.0 Hz, 2H), 4.47 (t, *J* = 6.2 Hz, 2H), 7.22 (t, *J* = 8.6 Hz, 1H), 7.61 (dd, *J* = 11.9, 2.1 Hz, 1H), 7.74 (ddd, *J* = 8.6, 2.1, 1.2 Hz, 1H). ^13^C-NMR (*d*_6_-DMSO, 101 MHz) δ (ppm): 21.42, 22.15, 22.30, 27.98, 56.77, 62.55, 63.03, 113.81, 116.55, 116.75, 122.53, 122.59, 127.14, 127.17, 139.48, 149.48, 150.18, 151.68, 151.78, 152.42, 154.77, 164.99, 165.91. ESI-HRMS Calcd. for C_19_H_21_N_2_O_7_F [M + H]^+^: 377.1507; found: 377.1515.

*3-[(3-Fluorobenzoyl)oxy]propyl 3,5,6-trimethylpyrazine-2-carboxylate* (**8k**). Compound **6** was treated with 3-fluoro-4-methoxybenzoic acid (**7k**) according to general procedure to give the desired product **8k** as a white solid. Yield 48%, ^1^H-NMR (CDCl_3_, 300 MHz) δ (ppm): 2.30 (m, 2H), 2.53 (s, 3H), 2.55 (s, 3H), 2.72 (s, 3H), 4.49 (t, *J* = 6.1 Hz, 2H), 4.57 (t, *J* = 6.3 Hz, 2H), 7.34 (t, *J* = 7.9 Hz, 1H), 7.50 (ddd, *J* = 8.0, 2.2, 1.1 Hz, 1H), 7.88 (dt, *J* = 7.7, 1.4 Hz, 1H), 7.95 (t, *J* = 1.9 Hz, 1H). ^13^C-NMR (*d*_6_-DMSO, 101 MHz) δ (ppm): 164.69, 162.86, 160.42, 154.07, 149.95, 148.81, 138.20, 129.30, 129.22, 124.15, 118.86, 114.83, 62.00, 61.66, 26.84, 20.22, 19.70, 18.84. ESI-HRMS Calcd. for C_18_H_19_N_2_O_4_F [M + Na]^+^: 369.1221; found: 369.1231.

*3-{[(3,4,5-Trimethoxyphenyl)acetyl]oxy}propyl 3,5,6-trimethylpyrazine-2-carboxylate* (**8l**). Compound **6** was treated with (3,4,5-trimethoxyphenyl)acetic acid (**7l**) according to general procedure to give the desired product **8l** as a white solid. Yield 43%, ^1^H-NMR (CDCl_3_, 300 MHz) δ (ppm): 2.17 (m, 2H), 2.57 (s, 6H), 2.73 (s, 3H), 3.57 (s, 2H), 3.83 (s, 3H), 3.86 (s, 6H), 4.27 (t, *J* = 6.2 Hz, 2H), 4.48 (t, *J* = 6.5 Hz, 2H), 6.51 (s, 2H).^13^C-NMR (CDCl_3_, 75 MHz) δ (ppm): 21.55, 22.16, 22.54, 27.95, 41.46, 56.06, 60.79, 61.48, 62.28, 106.23, 129.37, 137.04, 139.27, 149.29, 151.14, 153.20, 154.60, 165.84, 171.44. ESI-HRMS Calcd. for C_22_H_28_N_2_O_7_ [M + Na]^+^: 455.1789; found: 455.1783.

*3-{[(4-Bromophenyl)acetyl]oxy}propyl 3,5,6-trimethylpyrazine-2-carboxylate* (**8m**). Compound **6** was treated with (4-bromophenyl)acetic acid (**7m**) according to general procedure to give the desired product **8m** as a white solid. Yield 47%, ^1^H-NMR (*d*_6_-DMSO, 400 MHz) δ (ppm): 2.04 (m, 2H), 2.47 (s, 3H), 2.51 (s, 3H), 2.59 (s, 3H), 3.68 (s, 2H), 4.17 (t, *J* = 6.3 Hz, 1H), 4.35 (t, *J* = 6.4 Hz, 1H), 7.22 (d, *J* = 8.4 Hz, 2H), 7.49 (d, *J* = 8.4 Hz, 2H). ^13^C-NMR (*d*_6_-DMSO, 101 MHz) δ (ppm):21.48, 22.22, 22.31, 27.93, 31.94, 57.62, 61.77, 62.50, 120.49, 131.62, 132.10, 134.26, 134.40, 139.54, 149.60, 150.18, 154.89, 165.90, 171.26. ESI-HRMS Calcd. for C_19_H_21_N_2_O_4_Br [M + H]^+^: 443.0577; found: 443.0581.

*3-{[(3-Fluoro-4-methoxyphenyl)acetyl]oxy}propyl 3,5,6-trimethylpyrazine-2-carboxylate* (**8n**). Compound **6** was treated with (3-fluoro-4-methoxyphenyl)acetic acid (**7n**) according to general procedure to give the desired product **8n** as a white solid. Yield 47%. ^1^H-NMR (*d*_6_-DMSO, 400 MHz) δ (ppm): 6.86 (d, *J* = 7.6 Hz, 1H), 6.67 (d, *J* = 9.6 Hz, 1H), 6.41 (t, *J* = 8.5 Hz, 1H), 3.67 (t, *J* = 6.1 Hz, 3H), 3.60 (t, *J* = 6.1 Hz, 3H), 2.42 (s, 2H), 1.75 (s, 3H), 1.63 (s, 3H), 1.59 (s, 3H), 1.37–1.43 (m, 2H). ^13^C-NMR (*d*_6_-DMSO, 101 MHz) δ (ppm): 18.88, 19.73, 20.23, 26.74, 38.58, 54.50, 60.53, 61.33, 112.46, 115.53, 115.71, 124.09, 126.24, 126.31, 138.27, 148.89, 149.92, 154.13, 164.72, 170.97. ESI-HRMS Calcd. for C_20_H_23_N_2_O_5_F [M + H]^+^: 413.1483; found: 413.1484.

*3-{[(2E)-3-(3,4-Dimethoxyphenyl)prop-2-enoyl]oxy}propyl 3,5,6-trimethyl-pyrazine-2-carboxylate* (**8o**). Compound **6** was treated with (2E)-3-(3,4-dimethoxyphenyl)prop-2-enoic acid (**7o**) according to general procedure to give the desired product **8o** as a white solid. Yield 53%, ^1^H-NMR (*d*_6_-DMSO,400 MHz) δ (ppm): 2.12 (m, 2H), 2.45 (s, 3H), 2.46 (s, 3H), 2.61 (s, 3H), 3.79 (s, 3H), 3.80 (s, 3H), 4.28 (t, *J* = 6.2 Hz, 2H), 4.43 (t, *J* = 6.3 Hz, 2H), 6.50 (d, *J* = 15.9 Hz, 1H), 6.98 (d, *J* = 8.3 Hz, 1H), 7.18 (dd, *J* = 8.3, 2.0 Hz, 1H), 7.30 (d, *J* = 1.9 Hz, 1H), 7.54 (d, *J* = 15.9 Hz, 1H). ^13^C-NMR (*d*_6_-DMSO, 101 MHz) δ (ppm): 21.47, 22.16, 22.34, 28.11, 56.04, 56.06, 61.41, 62.83, 110.72, 111.93, 115.77, 123.43, 127.24, 139.54, 145.20, 149.41, 149.55, 150.20, 151.45, 154.83, 165.94, 166.94. ESI-HRMS Calcd. for C_22_H_26_N_2_O_6_ [M + Na]^+^:437.1683; found: 437.1686. 

*3-{[(2E)-3-(3,4,5-Trimethoxyphenyl)prop-2-enoyl]oxy}propyl 3,5,6-tri-methylpyrazine-2-carboxylate* (**8p**). Compound **6** was treated with (2E)-3-(3,4,5-trimethoxyphenyl)prop-2-enoic acid (**7p**) according to general procedure to give the desired product **8p** as a white solid. Yield 53%, ^1^H-NMR (CDCl_3_, 300 MHz,) δ (ppm): 2.18–2.25 (m, 2H), 2.53(s, 6H), 2.73(s, 3H), 3.88(s, 9H), 4.34-4.38(t, *J* = 6.2 Hz, 2H), 4.52-4.56(t, *J* = 6.4 Hz,2H), 6.31 (d, *J* = 15.9 Hz, 1H), 6.72(s, 2H), 7.56 (d, *J* = 15.9 Hz,1H). ^13^C-NMR (*d*_6_-DMSO, 101 MHz) δ (ppm): 21.46, 22.15, 22.36, 28.08, 32.11, 56.51, 57.76, 60.56, 61.55, 61.83, 62.83, 106.41, 117.60, 117.89, 130.11, 139.91, 145.09, 145.23, 149.57, 153.52, 154.85, 165.96,166.93. ESI- HRMS Calcd. for C_23_H_28_N_2_O_7_ [M + Na]^+^: 467.1789; found: 467.1759. 

*3-{[(2E)-3-(3,5,6-Trimethylpyrazin-2-yl)prop-2-enoyl]oxy}propyl 3,5,6-trimethylpyrazine-2-carboxylate* (**8q**). Compound **6** was treated with (2E)-3-(3,5,6-trimethylpyrazin-2-yl)prop-2-enoic acid (**7q**) according to general procedure to give the desired product **8q** as a white solid. Yield 53%, ^1^H-NMR (CDCl_3_, 300 MHz,) δ (ppm): 2.22 (m, 2H), 2.54–2.50 (m, 12H), 2.59 (s, 3H), 2.73 (s, 3H), 4.37 (t, *J* = 6.2 Hz, 2H), 4.53 (t, *J* = 6.4 Hz, 2H), 7.02 (d, *J* = 15.3 Hz, 1H), 7.83(d, *J* = 15.3 Hz, 1H). ^13^C-NMR (CDCl_3_, 126 MHz) δ (ppm): 166.84,166.53, 165.64, 154.41, 152.49, 151.02, 149.84, 149.20, 148.70, 142.38, 138.70, 122.75, 61.58, 58.45, 31.61, 22.41, 21.95, 21.76, 21.55, 21.34, 20.43. ESI-HRMS Calcd. for C_21_H_26_N_4_O_4_ [M + Na]^+^: 421.1846; found: 421.1848. 

*3-[(Pyridine-2-carbonyl)oxy]propyl 3,5,6-trimethylpyrazine-2-carboxylate* (**8r**). Compound **6** was treated with pyridine-2-carboxylic acid (**7r**) according to general procedure to give the desired product **8r** as a white solid. Yield 53%, ^1^H-NMR (CDCl_3_, 300 MHz,) δ (ppm): 2.31 (m, 2H), 2.54 (s, 3H), 2.55 (s, 3H), 2.73 (s, 3H), 4.52 (t, *J* = 6.2 Hz, 2H), 4.57 (t, *J* = 6.3 Hz, 2H), 7.37 (dd, *J* = 8.0, 4.9 Hz, 1H), 8.27 (dt, *J* = 8.0, 1.9 Hz, 1H), 8.76 (dd, *J* = 4.8, 1.6 Hz, 1H), 9.19 (d, *J* = 1.9 Hz, 1H). ^13^C-NMR (CDCl_3_, 75 MHz) δ (ppm): 21.55, 22.17, 22.56, 28.04, 62.27 (d, *J* = 17.9 Hz), 123.26, 125.91, 137.00, 139.18, 149.29, 150.86, 151.23, 153.44, 154.62, 165.12, 165.84. ESI-HRMS Calcd. for C_17_H_19_N_3_O_4_ [M + Na]^+^: 352.1268; found: 352.1271.

### 3.4. Biological Activity

#### 3.4.1. Inhibitory Activities of AChE and BuChE

Acetylcholinesterase (AChE, E.C. 3.1.1.7, from electric eel), butyrylcholinesterase (BuChE, E.C. 3.1.1.8, from equine serum), acetylthiocholine chloride (ATC), butyrylthiocholine chloride (BTC), and 5,5′-dithiobis-(2-nitrobenzoic acid) (Ellman’s reagent, DTNB) were purchased from Merck & Co. Tacrine hydrochloride (Darmstadt, Germany) and galanthamine hydrobromide were used as the reference and purchased from Sigma-Aldrich (Santa Clara, CA. USA,). Photometric 96-well microplates made from polystyrene (Thermo Scientific, Waltham, CA. USA) were used for measurement purposes.

AChE and BuChE inhibitory activities were measured by the spectrophotometric method of Ellman, with slight modification [29]. Briefly, all in vitro assays were performed in 0.1 M NaH_2_PO_4_/Na_2_HPO_4_ buffer, pH 7.4, using a THERMO Enzyme-labeled Instrument. The enzyme solutions were prepared to obtain 2.0 units/mL in 2.0 mL aliquots. The assay medium (1 mL) consisted of phosphate buffer (pH 7.4), 10 μL of the enzyme, 30 μL of 0.01 M DTNB, and 30 μL of substrate (ATC or BTC) at a concentration of 0.01 M. The test and reference compounds were first dissolved in DMSO (5‰) and fetal bovine serum (1‰), and then diluted in 0.1 M NaH_2_PO_4_/Na_2_HPO_4_ buffer (pH 7.4) to provide a final concentration range. The inhibitors were added to the assay solution containing enzyme, buffer, and DTNB with pre-incubation for 20 min at 37 °C, prior to the addition of the substrate. The activity was determined by measuring the increase in absorbance at 410 nm at 1 min intervals at 37 °C. The data were calculated according to the method described by Ellman et al. Each concentration was assayed in triplicate. Blanks containing all of the components except AChE were included in the analyses.

#### 3.4.2. Inhibition of Self-Mediated Aβ (1-42) Aggregation

A Thioflavin T-based fluorometric assay was performed in order to investigate the self-induced Aβ (1-42) aggregation [27]. Briefly, compounds to be tested were firstly dissolved in DMSO to obtain a 10 mM solution and then diluted in phosphate buffer (pH 7.4) to provide a final concentration range. Aβ (1-42) peptide (Sigma-Aldrich) was prepared in 10 mM phosphate buffer (pH 7.4) to give a final concentration of 20 μM solution and incubated at 37 °C for 24 h with the inhibitors at different concentrations (0, 5, 10, 20, 50, 100, 150, 200, 250, 300 μM). After incubation, thioflavin-T (5 μM in 50 mM glycine-NaOH buffer, pH 8.0) was added into the above 96-well microplate and the samples were diluted to a final volume of 150 μL. Fluorescence was measured at 450 nm (λex) and 485 nm (λem) on a Varioskan Flash Multimode Reader (Thermo Scientific). Each inhibitor was run in triplicate.

#### 3.4.3. Transmission Electron Microscopy (TEM) Assay

Aβ (1-42) peptide (Sigma-Aldrich) was added into a 96-well microplate and then diluted by phosphate buffer (10 mM, pH 7.4) to provide a concentration of 100 μM solution at 4 °C before use and the sample preparation procedure was the same as that used for the Thioflavin T assay. Aliquots (5 μL) of the samples were placed on a carbon-coated copper/rhodium grid for 2 min at room temperature. Each grid was stained with phosphomolybdic acid solution (3%, 5 μL) for 2 min. After draining off the excess staining solution, the specimen was transferred for imaging by transmission electron microscopy.

#### 3.4.4. Kinetic Study of AChE Inhibition

The assay solution (100 μL) was afforded by the addition of 30 μL of DTNB (0.01 M), 10 μL of 2 units/mL AChE, and 30 μL of substrate (ATC) to the phosphate buffer (0.1 M, pH 7.0). Three different concentrations of **8q** were added to the assay solution with the AChE and pre-incubated for 20 min at 37 °C, followed by the addition of substrate in different concentrations. Kinetic characterization of the hydrolysis of ATC catalysed by AChE was carried out at 410 nm. A parallel control was made with the assay solution of no inhibitor for each time. The plots were assessed by a weighted least square analysis that assumed the variance of V to be a constant percentage of V for the entire data set. Slopes of these reciprocal plots were then plotted against the concentration of **8q** in a weighted analysis, and Km was determined as the intercept on the negative x-axis.

#### 3.4.5. In Vitro Cytotoxicity of Tacrine and Ligustrazine Hybrids **8q**, **8r** in HepG2 Cells

HepG2 cells (human hepatocellular liver carcinoma cell line from American Type Culture Collection, ATCC) were cultured in Eagle’s minimum essential medium (EMEM) supplemented with 10% heat-inactivated fetal bovine serum (FBS), and 100 U/mL of penicillin/streptomycin (Gino Biomedical Technology Co. LTD., Hangzhou, China). Cultures were seeded into flasks containing supplemented medium and maintained at 37 °C in a humidified atmosphere of 5% CO_2_. For assays, cells (1.0 × 10^5^ cells/well) were seeded in a 96-well plate in complete medium, the medium was removed after 24 h, and cells were exposed to the increasing concentrations of compounds **8q** or **8r** (0, 25, 50, 125, 200, 300, 400, and 500 μM) in DMEM with no serum for a further 24 h. Cell survival was measured through a CCK-8 assay. All compounds were dissolved in pure DMSO.

#### 3.4.6. In Vitro Cytotoxicity of Tacrine and Compounds **8q**, **8r** in SH-SY5Y Cells

SH-SY5Y cells (human neuroblastoma cell line from American Type Culture Collection, ATCC, Manassas, VA, USA) were seeded into 96-well plates at a density of 4 × 10^4^ cells/mL in Dulbecco’s modified Eagle’s medium (DMEM) supplemented with 10% FBS and 100 U/mL of penicillin/streptomycin (Gino Biomedical Technology Co. LTD., Hangzhou, China), and incubated in a humidified atmosphere containing 5% CO_2_ at 37 °C. For the experiments, cells (1.0 × 105 cells/well) were seeded in a 96-well plate in complete medium; after 24 h, cells were treated with the test compound in different concentrations (0, 25, 50, 125, 200, 300, 400, and 500 μM) at 37 °C for 24 h. After this incubation, 10 μL/well of CCK-8 (5 mg/mL) was added and incubated at 37 °C for 4 h. The optical density (OD) of each well was measured using an Epoch Microplate Spectrophotometer (BioTek Instruments, Inc., Winooski, VT, USA) with a test wavelength of 450, 630 nm.

### 3.5. Molecular Docking

For the docking studies, the Surflex dock in Sybyl X-2.1.1 (Certara, L.P., Princeton, NJ, USA) was used. The structures of **8q** and **8r** were built in Sybyl and the energy was minimized with the conjugate gradient method. For the protein, all the water molecules were extracted and the AMBER FF99 force field was applied. The standard docking procedure was used with the parameters set in default. Re-docking the original ligand into the crystal structure was performed to test the reliability of the docking strategy. Binding modes and interactions were analyzed in Pymol.

## 4. Conclusions

In summary, a series of novel ligustrazine derivatives **8a**–**8r** have been designed, synthesized, and evaluated for the first time as multi-targeted inhibitors for anti-AD drug discovery. The results demonstrated that: (1) most of the target compounds exhibited potent AChE inhibitory activities, even at a nanomolar level, with excellent selectivity towards AChE. In particular, compounds **8q** and **8r** were found to be the most active inhibitors against AChE, with IC_50_ values of 1.39 and 0.25 nM, respectively, and the highest selectivity for AChE (for **8q**, IC_50_ BuChE/IC_50_ AChE = 2.91 × 10^6^; for **8r**, IC_50_ BuChE/IC_50_ AChE = 1.32 × 10^7^). Additionally, (2) the two most potent AChE inhibitors **8q** and **8r** possessed acceptable inhibitory activities against self-induced Aβ (1-42) aggregation, with IC_50_ values of 17.36 µM and 49.14 µM, respectively. The SAR analysis indicated that the introduction of a proper and relatively rigid group between the ester group and the benzene ring was beneficial to AChE inhibitory activity, and the replacement of the benzene ring moiety with a pyrazine unit or pyridine core also led to a dramatic increase in AChE inhibitory activity. A combined Lineweaver-Burk plot and molecular docking study revealed that these compounds might act as mixed-type inhibitors to exhibit such effects via selectively targeting both the CAS and the PAS of AChE. Moreover, the investigations of both the HepG2 cells and the SH-SY5Y cells showed that the potent “hits” **8q** and **8r** have a potent safe window for the next stage of the development of anti-AD drug discovery. Taken together, these results suggested that **8q** and **8r** could serve as promising candidates for the treatment of AD disorders and also provide a rational basis for the development of future multi-targeted inhibitors as anti-AD drugs with validated advantages over the classical anti-AD drug tacrine.

## Figures and Tables

**Table 1:** Inhibition of ChEs activity, affinity ratios, and inhibition of Aβ (1-42) self-induced aggregation ^a,b^.

Compounds	IC_50_^c^ for AChE (nM)	IC_50_ for BuChE (mM)	Selectivity Index ^f^	IC_50_ for Aβ (1-42) Aggregation (µM) ^g^
**8a**	167.9 ± 0.39	3.63 ± 0.07	2.16 × 10^4^	NA
**8b**	4.43 ± 0.35	5.64 ± 0.38	1.27 × 10^6^	3.66 ± 0.104
**8c**	387.9 ± 0.45	2.70 ± 0.03	5.16 × 10^3^	22.54 ± 0.014
**8d**	4.12 ± 0.24	4.29 ± 0.12	1.02 × 10^6^	51.81 ± 0.047
**8e**	143.8 ± 0.25	0.34 ± 0.03	2.36 × 10^3^	55.86 ± 0.035
**8f**	6.61 ± 0.13	3.27 ± 0.06	4.95 × 10^5^	45.88 ± 0.024
**8g**	NA ^d^	21.13 ± 0.46	6.23	NA
**8h**	NA	2.49 ± 0.29	2.62	NA
**8i**	NA	112.74 ± 0.47	1.14 × 10^2^	211.18 ± 0.039
**8j**	NA	0.857 ± 0.002	2.97	7.12 ± 0.012
**8k**	NA	5.49 ± 0.15	9.49	5.10 ± 0.040
**8l**	303.4 ± 0.26	3.96 ± 0.26	1.31 × 10^4^	77.45 ± 0.029
**8m**	NA	373.52 ± 0.78	52.17	200.84 ± 0.016
**8n**	NA	3.06 ± 0.34	20.67	NA
**8o**	3.24 ± 0.10	3.97 ± 0.10	1.23 × 10^6^	45.29 ± 0.033
**8p**	93.51 ± 0.20	4.02 ± 0.03	4.30 × 10^4^	71.28 ± 0.044
**8q**	1.39 ± 0.33	4.04 ± 0.91	2.91 × 10^6^	17.36 ± 0.027
**8r**	0.25 ± 0.39	3.30 ± 0.06	1.32 × 10^7^	49.14 ± 0.025
**Tacrine**	73.36 ± 0.22	14.45 ± 0.06 ^e^	0.20	12.21 ± 0.02
**Galanthamine**	23.74 ± 0.06	0.180 ± 0.004	7.58 × 10^3^	NA

^a^ Data was expressed as mean ± SEM of at least three independent experiments. ^b^ All tested compounds were soluble at a final concentration in each assay. ^c^ IC_50_, inhibitor concentration for 50% inactivation of AChE. ^d^ No potent inhibitory activity. ^e^ Unit of nM (nanomolar). ^f^ Selectivity index = IC_50_ (BChE)/IC_50_ (AChE). ^g^ The thioflavin-T fluorescence method was used and the measurements were carried out in the presence of 0, 5, 10, 20, 50, 100, 150, 200, 250, and 300 μM tested compounds, respectively.

**Table 2:** In vitro cytotoxicity of tacrine and ligustrazine hybrids** 8q** and** 8r **in HepG2 cells ^a,b^.

Compounds	Control	25 µM	50 µM	125 µM	200 µM	300 µM	400 µM	500 µM
**8q**	100 ± 0.82	96.81 ± 3.05 ^ns^	91.09 ± 0.99 ^ns^	81.19 ± 1.48 **	64.81 ± 0.54 **	66.75 ± 1.11 **	68.89 ± 0.51 **	70.10 ± 1.98 **
**8r**	100 ± 0.86	102.83 ± 0.57 ^ns^	88.05 ± 1.12 ^ns^	81.72 ± 0.87 **	71.10 ± 1.06 **	71.10 ± 0.80 **	68.85 ± 1.11 **	68.31 ± 0.53 **
**Tacrine**	100 ± 2.87	99.78 ± 1.07 ^ns^	95.02 ± 2.30 ^ns^	83.72 ± 0.73 **	81.80 ± 1.86 **	74.32 ± 3.93 **	23.61 ± 0.05 **	19.92 ± 0.05 **

^a^ Cell viability was determined by a Cell Counting Kit-8 (CCK-8) assay and all compounds were assayed at increasing concentrations (1–500 µM, as indicated); ^b^ Data are normalized as a percentage of control and are expressed as the means ± SEM of triplicates from at least three different cultures. ** *p* ≤ 0.01 ^ns^ Not significant with respect to the control group.

**Table 3:** In vitro cytotoxicity of tacrine and compounds **8q** and **8r** in SH-SY5Y cells ^a,b^.

Compounds	Control	25 µM	50 µM	125 µM	200µM	300 µM	400 µM	500 µM
**8q**	100 ± 0.28	104.30 ± 0.29 ^ns^	96.08 ± 0.78 ^ns^	80.25 ± 1.53 **	72.17 ±0.54 **	58.92 ± 0.70 **	55.14 ± 0.50 **	55.28 ± 0.55 **
**8r**	100 ± 1.75	94.51 ± 0.45 ^ns^	91.46 ± 0.29 ^ns^	83.23 ± 0.78 *	83.26 ± 2.74 *	68.43 ± 0.48 **	62.68 ± 0.56 **	66.38 ± 1.57 **
**Tacrine**	100 ± 1.86	87.89 ± 0.85 ^ns^	88.17 ± 1.55 ^ns^	75.42 ± 0.78 **	48.75 ± 0.43 **	43.39 ± 2.69 **	34.69 ± 2.86 **	27.93 ± 1.44 **

^a^ Cell viability was determined by a Cell Counting Kit-8 (CCK-8) assay and all compounds were assayed at increasing concentrations (1–500 µM, as indicated); ^b^ Data are normalized as a percentage of the control and are expressed as the means ± SEM of triplicates from at least three different cultures. ** *p* ≤ 0.01. * *p* ≤ 0.05 ^ns^ Not significant with respect to the control group.

## Supplementary Materials

The following are available online.
