# Supplementary material for: Synthesis and Evaluation of Novel Ligustrazine Derivatives as Multi-Targeted Inhibitors for the Treatment of Alzheimer’s Disease
Source: Molecules. 2018 Oct 5;23(10):2540. doi: 10.3390/molecules23102540 (PMC6222487; doi:10.3390/molecules23102540)
Supplement: Supplementary file 1 [file molecules-23-02540-s001.pdf]

Supporting Information

for

Synthesis and Evaluation of Novel Ligustrazine Derivatives as Multi-Targeted Inhibitors for the Treatment of Alzheimer's Disease

Wenhao Wu[†], Xintong Liang[†], Guoquan Xie, Langdi Chen, Weixiong Liu, Guolin Luo, Peiquan Zhang, Lihong Yu, Xuehua Zheng, Hong Ji, Chao Zhang* and Wei Yi*

Key Laboratory of Molecular Target & Clinical Pharmacology, School of Pharmaceutical Sciences, Guangzhou Medical University, Guangzhou 511436, Guangdong, China

Email: chao-zh@163.com and yiwei@gzhmu.edu.cn.

[†] These authors contributed equally to this work

^{1}H NMR (300 MHz, $CDCl_3$) of **8a**

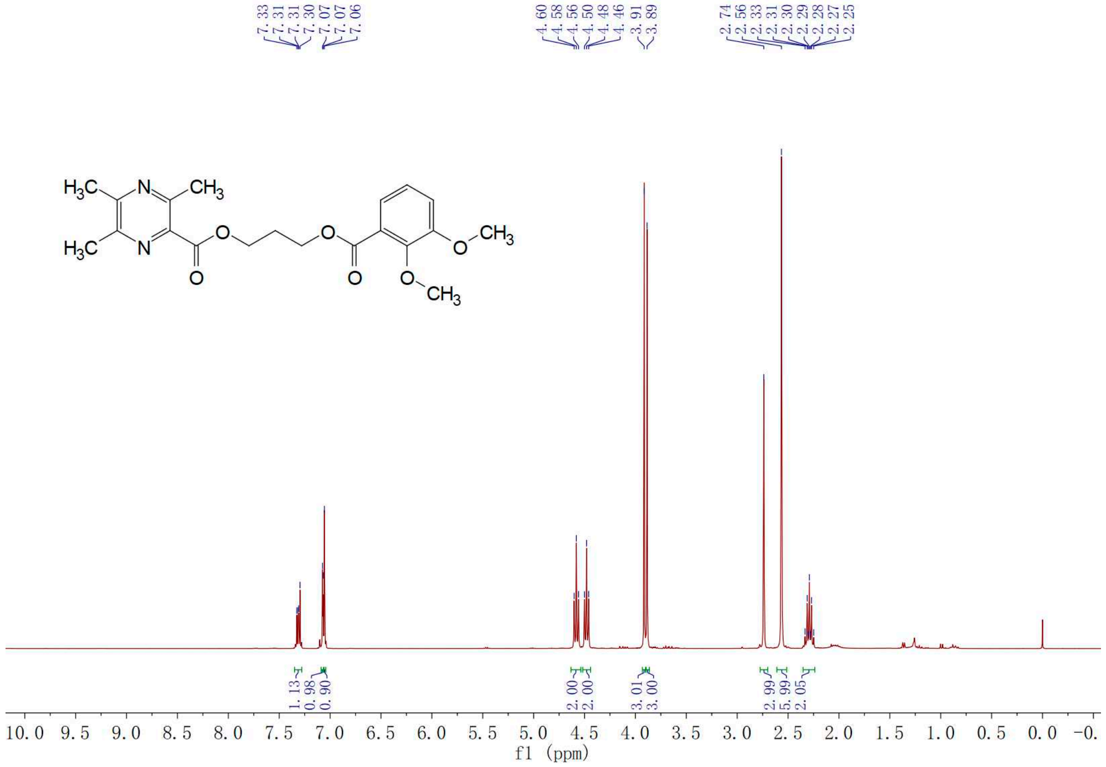

^{13}C NMR (75 MHz, $CDCl_3$) of **8a**

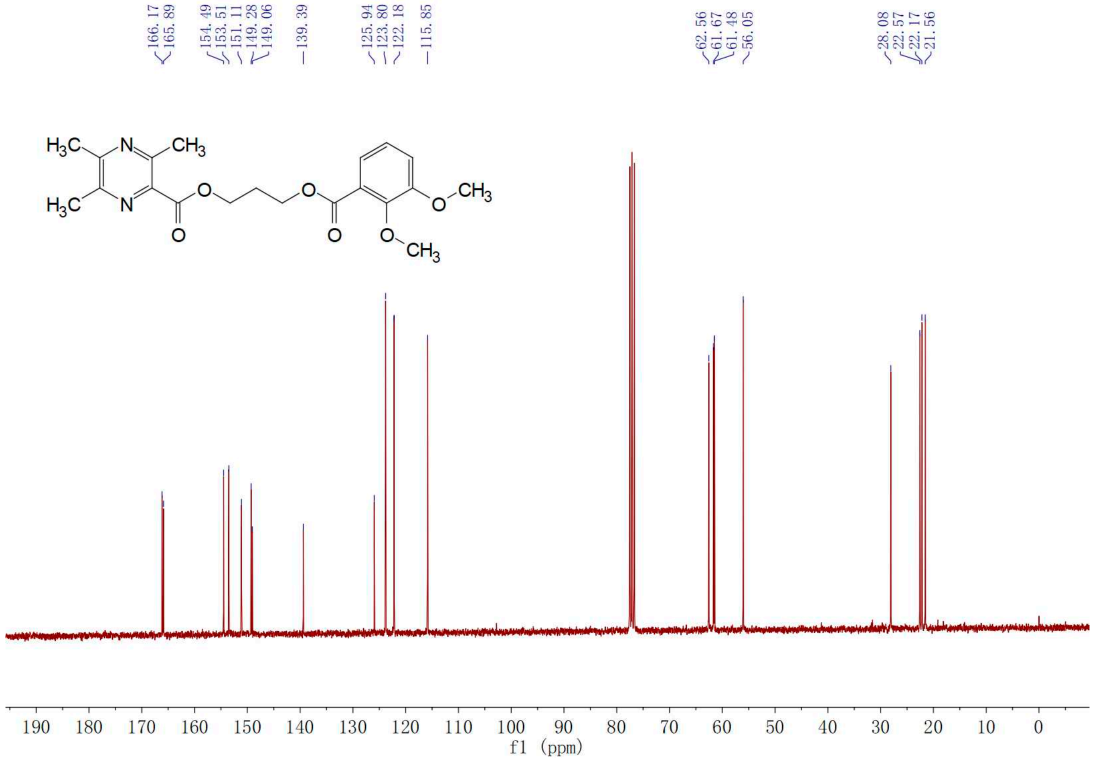

HRMS spectra of **8a**

Data File: I:\20170411-GY\13.lcd

Elmt	Val.	Min	Max	Elmt	Val.	Min	Max	Elmt	Val.	Min	Max	Elmt	Val.	Min	Max	Use Adduct
H	1	0	60	O	2	0	6	P	3	0	0	Cu	2	0	0	H
B	3	0	0	18O	2	0	0	S	2	0	0	Br	1	0	1	Na
C	4	0	50	F	1	0	1	Cl	1	0	0	I	3	0	0	
N	3	0	5	Si	4	0	0	Ni	2	0	0					

Error Margin (ppm):	20	DBE Range:	-2.0 - 1000.0	Electron Ions:	both
HC Ratio:	unlimited	Apply N Rule:	yes	Use MSn Info:	no
Max Isotopes:	all	Isotope RI (%):	1.00	Isotope Res:	10000
MSn Iso RI (%):	75.00	MSn Logic Mode:	AND	Max Results:	500

Event#: 1 MS(E+)   Ret. Time : 1.120   Scan# : 169

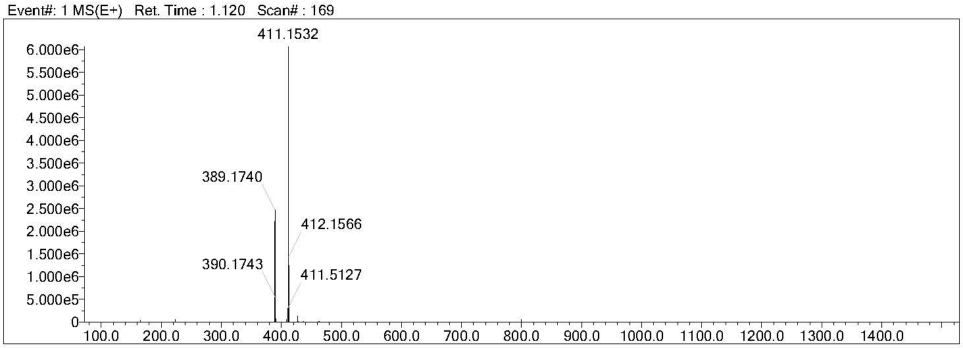

Measured region for 411.1532 m/z

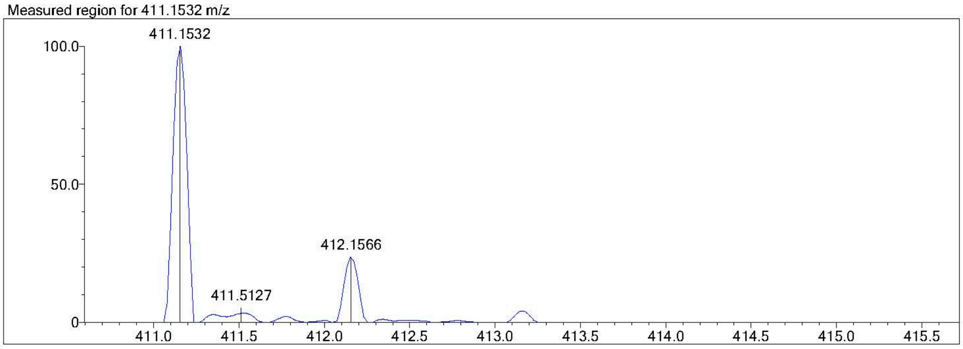

C20 H24 N2 O6 [M+Na]+ : Predicted region for 411.1527 m/z

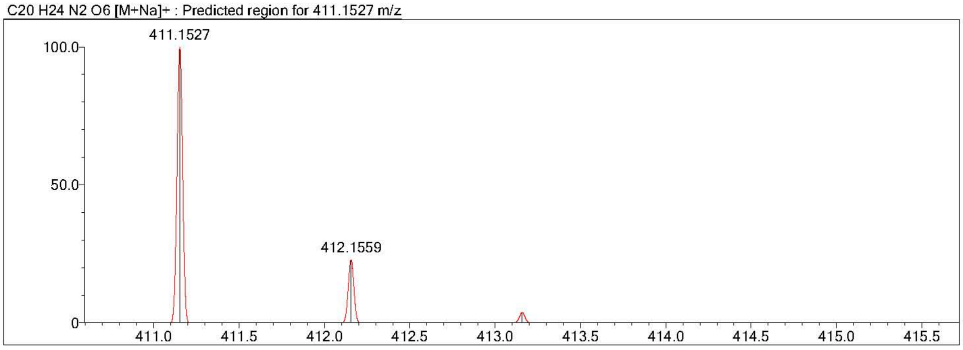

Rank	Score	Formula (M)	Ion	Meas. m/z	Pred. m/z	Df. (mDa)	Df. (ppm)	Iso	DBE
1	88.64	C20 H24 N2 O6	[M+Na]+	411.1532	411.1527	0.5	1.22	89.13	10.0

^{1}H NMR (300 MHz, $CDCl_3$) of **8b**

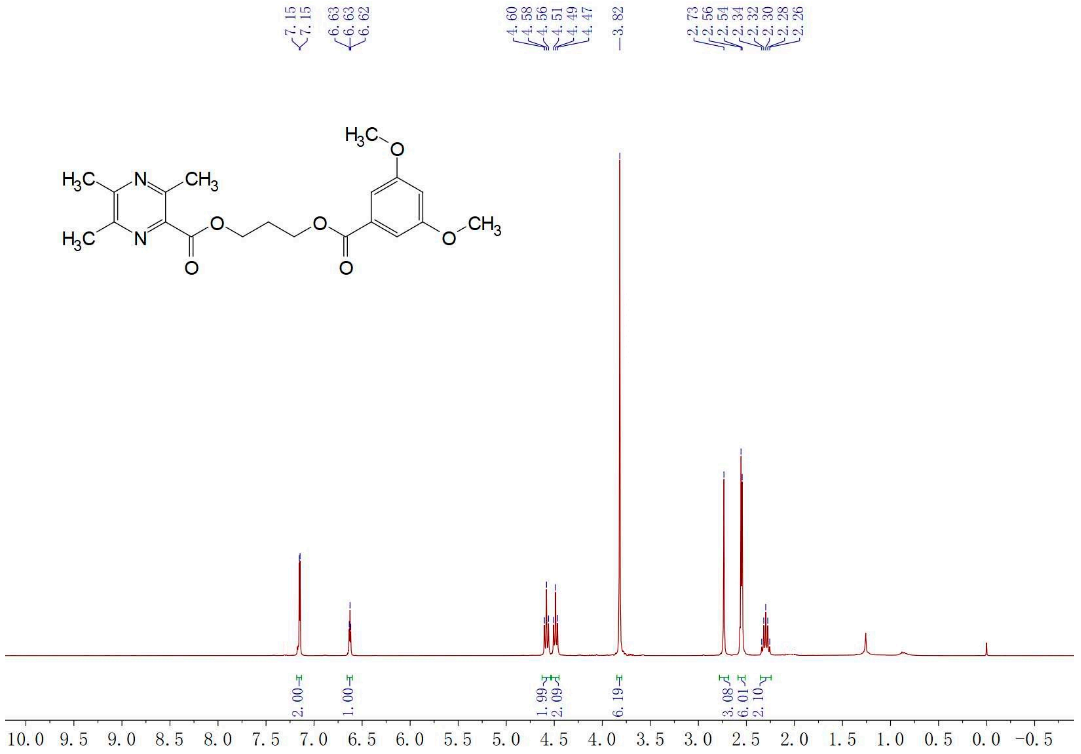

^{13}C NMR (75 MHz, $CDCl_3$) of **8b**

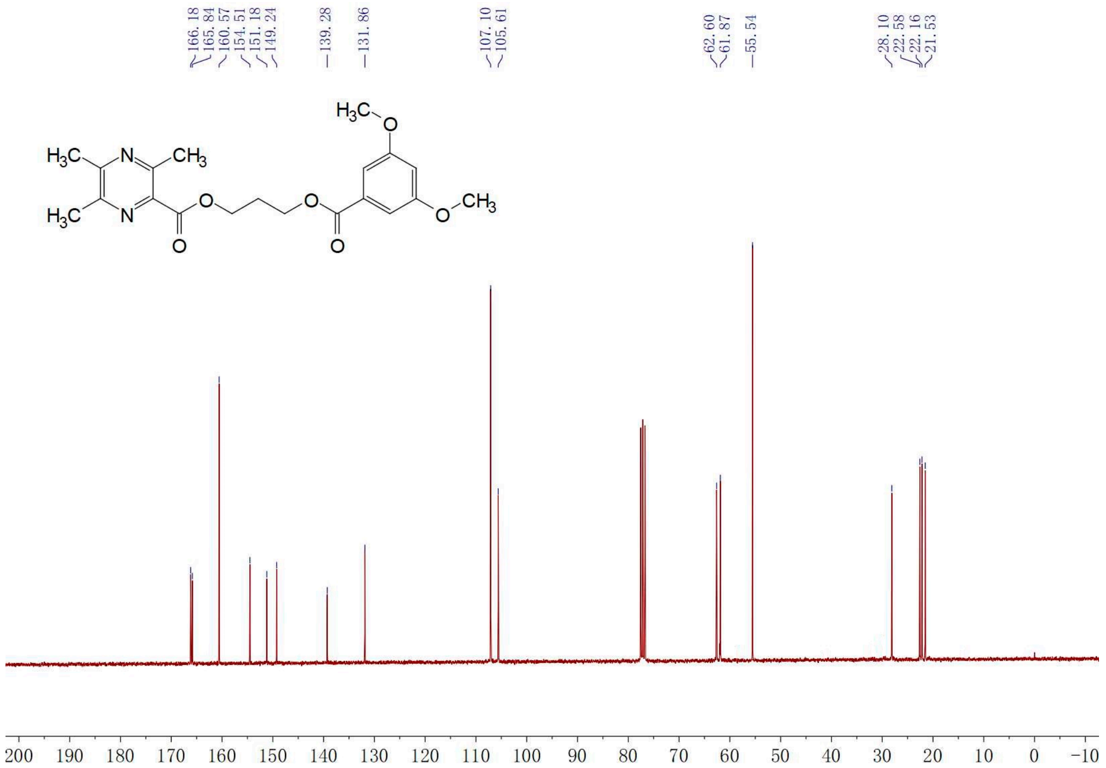

HRMS spectra of **8b**

Formula Predictor Report - 14.lcd

Data File: I:\20170411-GY\14.lcd

Elmt	Val.	Min	Max	Elmt	Val.	Min	Max	Elmt	Val.	Min	Max	Elmt	Val.	Min	Max	Use Adduct
H	1	0	60	O	2	0	6	P	3	0	0	Cu	2	0	0	H
B	3	0	0	18O	2	0	0	S	2	0	0	Br	1	0	1	Na
C	4	0	50	F	1	0	1	Cl	1	0	0	I	3	0	0	
N	3	0	5	Si	4	0	0	Ni	2	0	0					

Error Margin (ppm): 20
HC Ratio: unlimited
Max Isotopes: all
MSn Iso RI (%): 75.00

DBE Range: -2.0 - 1000.0
Apply N Rule: yes
Isotope RI (%): 1.00
MSn Logic Mode: AND

Electron Ions: both
Use MSn Info: no
Isotope Res: 10000
Max Results: 500

Event#: 1 MS(E+) Ret. Time : 1.027 - 1.080 -> 1.242 Scan# : 155 - 163 -> 187

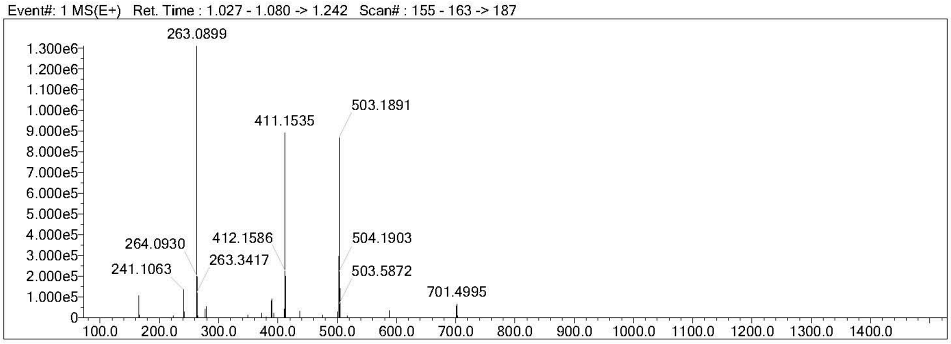

Measured region for 411.1535 m/z

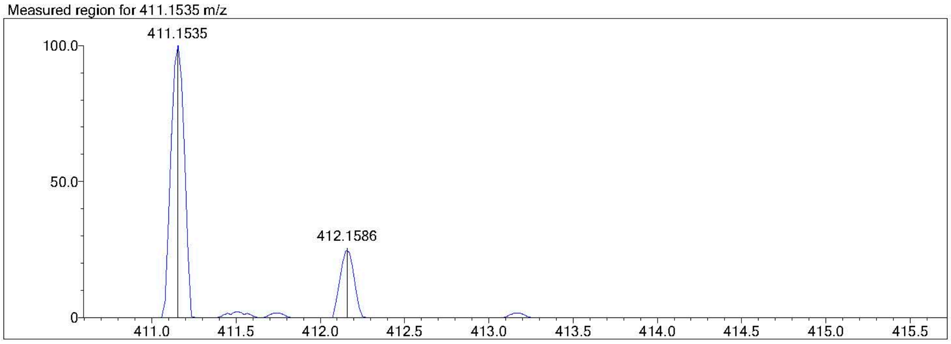

C20 H24 N2 O6 [M+Na]+ : Predicted region for 411.1527 m/z

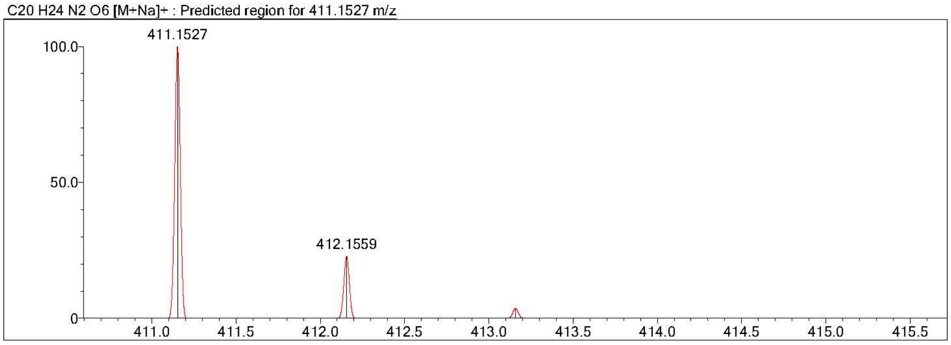

Rank	Score	Formula (M)	Ion	Meas. m/z	Pred. m/z	Df. (mDa)	Df. (ppm)	Iso	DBE
1	79.85	C20 H24 N2 O6	[M+Na]+	411.1535	411.1527	0.8	1.95	81.80	10.0

^{1}H NMR (300 MHz, $CDCl_3$) of **8c**

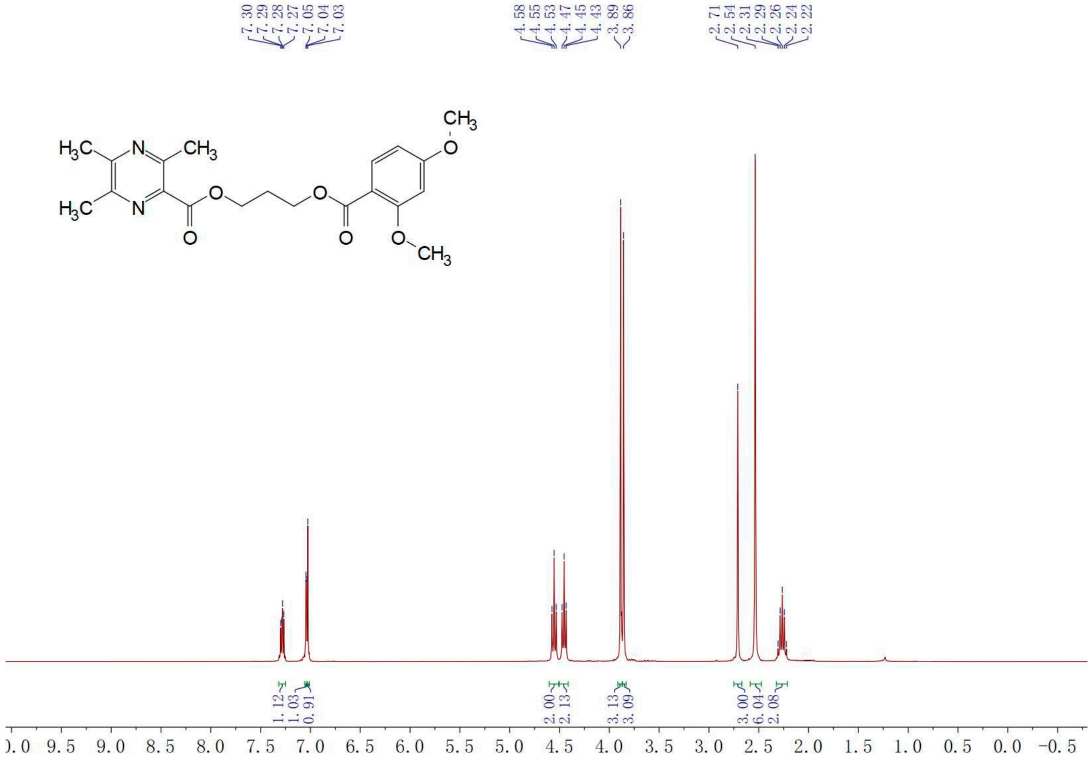

^{13}C NMR (75 MHz, $CDCl_3$) of **8c**

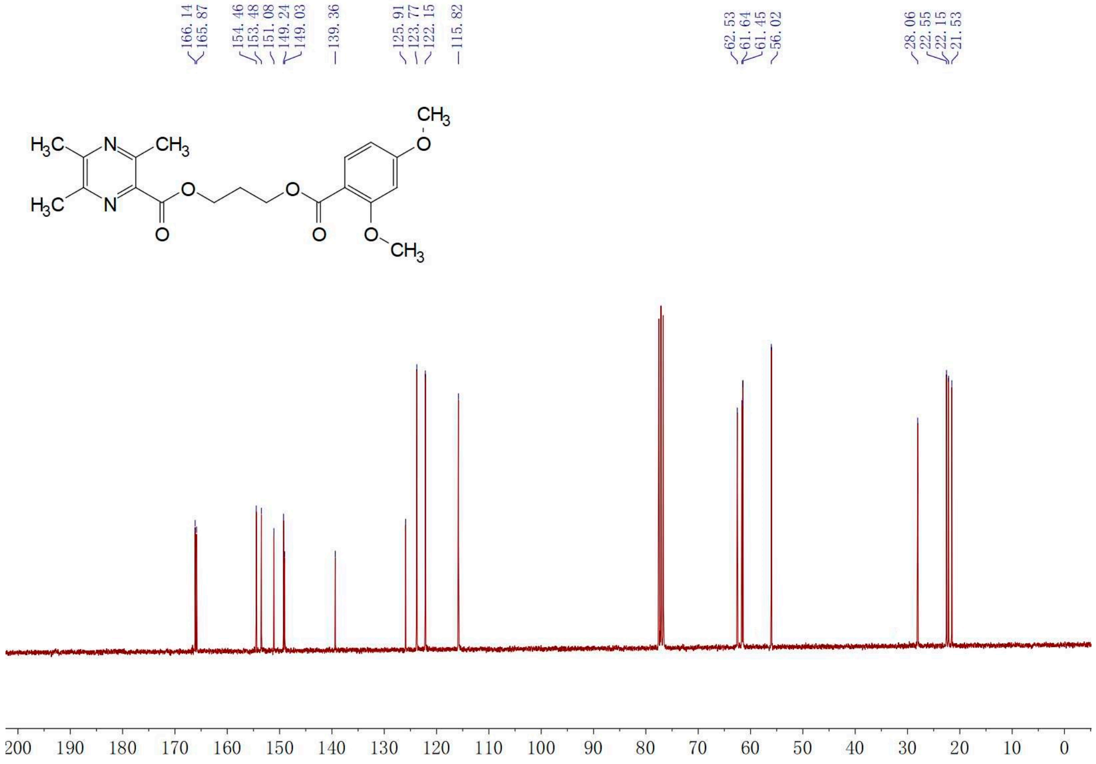

HRMS spectra of **8c**

Data File: I:\20170411-GY\16.lcd

Elmt	Val.	Min	Max	Elmt	Val.	Min	Max	Elmt	Val.	Min	Max	Elmt	Val.	Min	Max	Use Adduct
H	1	0	60	O	2	0	7	P	3	0	0	Cu	2	0	0	H
B	3	0	0	18O	2	0	0	S	2	0	0	Br	1	0	1	Na
C	4	0	50	F	1	0	1	Cl	1	0	0	I	3	0	0	
N	3	0	5	Si	4	0	0	Ni	2	0	0					

Error Margin (ppm):	20	DBE Range:	-2.0 - 1000.0	Electron Ions:	both
HC Ratio:	unlimited	Apply N Rule:	yes	Use MSn Info:	no
Max Isotopes:	all	Isotope RI (%):	1.00	Isotope Res:	10000
MSn Iso RI (%):	75.00	MSn Logic Mode:	AND	Max Results:	500

Event#: 1 MS(E+) Ret. Time : 1.187 - 1.240 -> 1.439 Scan# : 179 - 187 -> 217

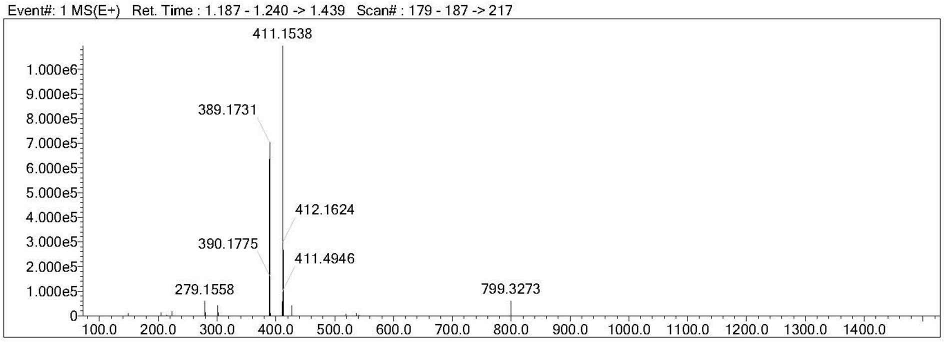

Measured region for 411.1538 m/z

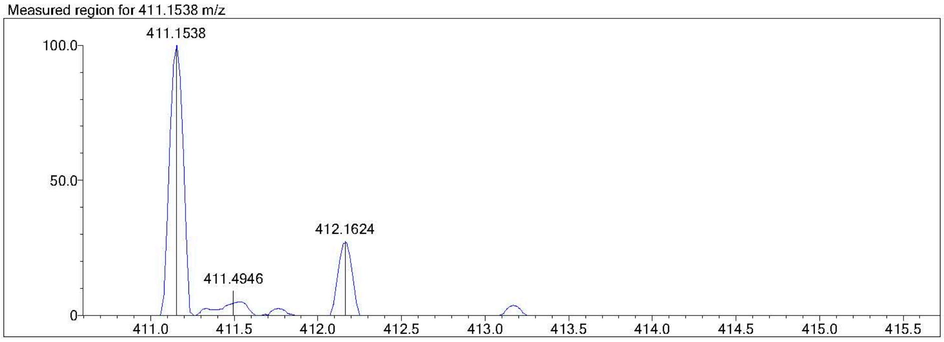

C20 H24 N2 O6 [M+Na]+ : Predicted region for 411.1527 m/z

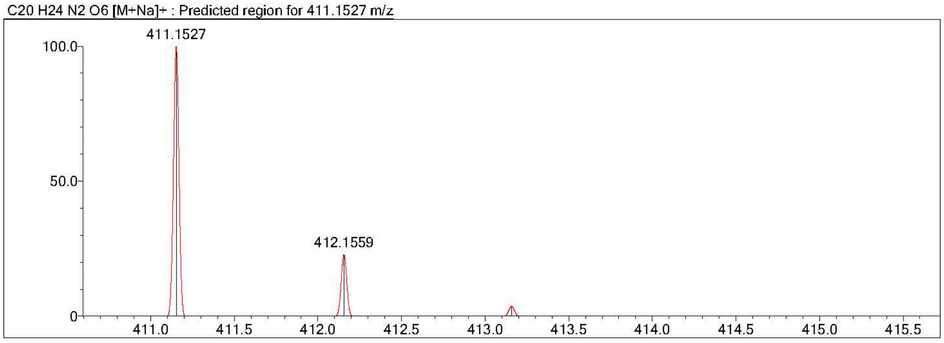

Rank	Score	Formula (M)	Ion	Meas. m/z	Pred. m/z	Df. (mDa)	Df. (ppm)	Iso	DBE
2	79.58	C20 H24 N2 O6	[M+Na]+	411.1538	411.1527	1.1	2.68	83.07	10.0

^{1}H NMR (400 MHz, DMSO-d6) of **8d**

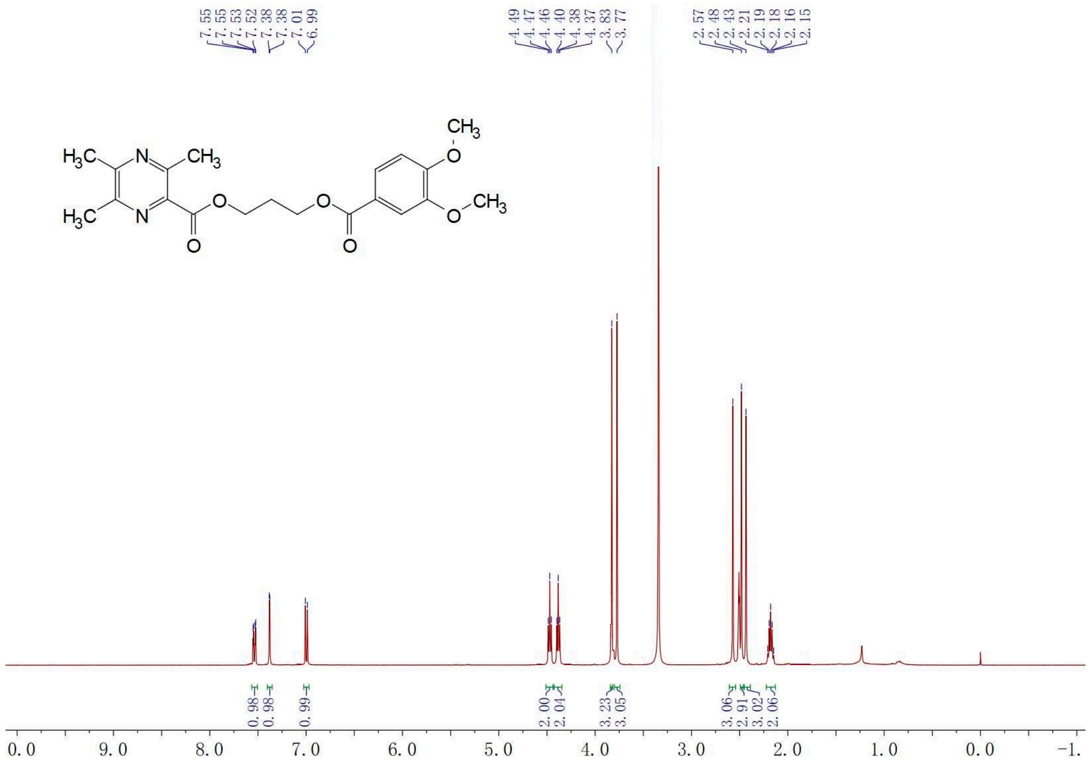

^{13}C NMR (100 MHz, DMSO-d6) of **8d**

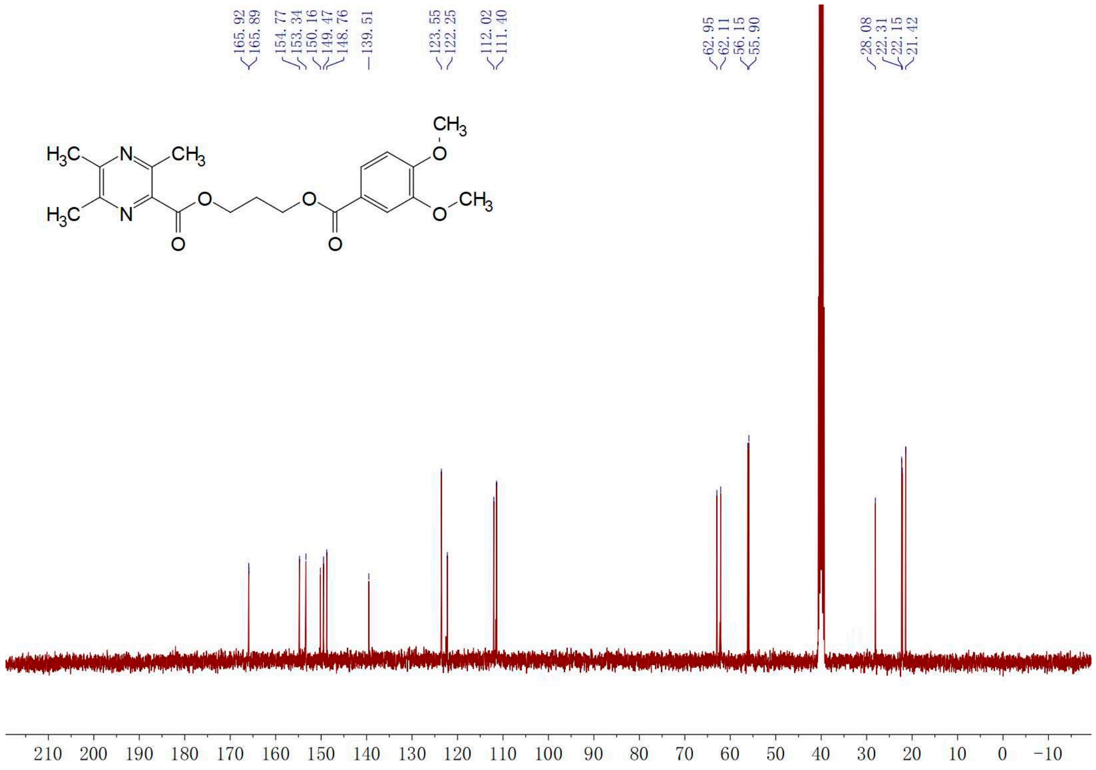

HRMS spectra of **8d**

Data File: I:\20170411-GY\17.lcd

Elmt	Val.	Min	Max	Elmt	Val.	Min	Max	Elmt	Val.	Min	Max	Elmt	Val.	Min	Max	Use Adduct
H	1	0	60	O	2	0	7	P	3	0	0	Cu	2	0	0	H
B	3	0	0	18O	2	0	0	S	2	0	0	Br	1	0	1	Na
C	4	0	50	F	1	0	1	Cl	1	0	0	I	3	0	0	
N	3	0	5	Si	4	0	0	Ni	2	0	0					

Error Margin (ppm):	20	DBE Range:	-2.0 - 1000.0	Electron Ions:	both
HC Ratio:	unlimited	Apply N Rule:	yes	Use MSn Info:	no
Max Isotopes:	all	Isotope RI (%):	1.00	Isotope Res:	10000
MSn Iso RI (%):	75.00	MSn Logic Mode:	AND	Max Results:	500

Event#: 1 MS(E+) Ret. Time : 1.187 - 1.280 -> 1.555 Scan# : 179 - 193 -> 235

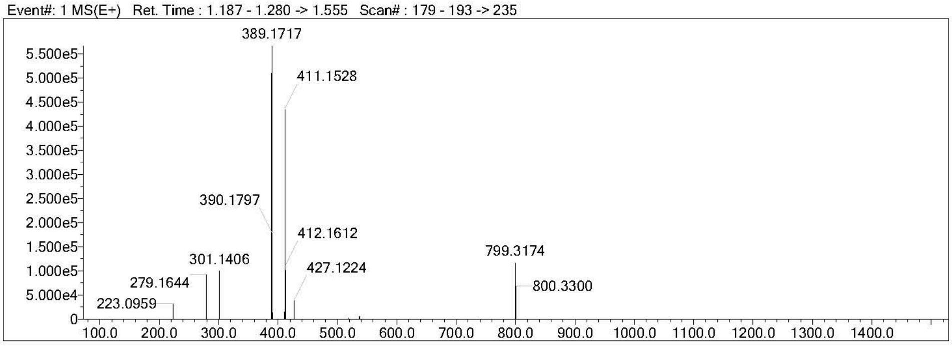

Measured region for 411.1528 m/z

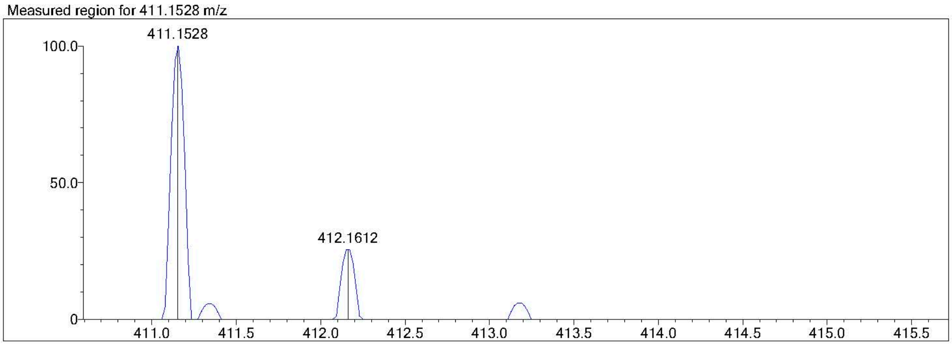

C20 H24 N2 O6 [M+Na]+ : Predicted region for 411.1527 m/z

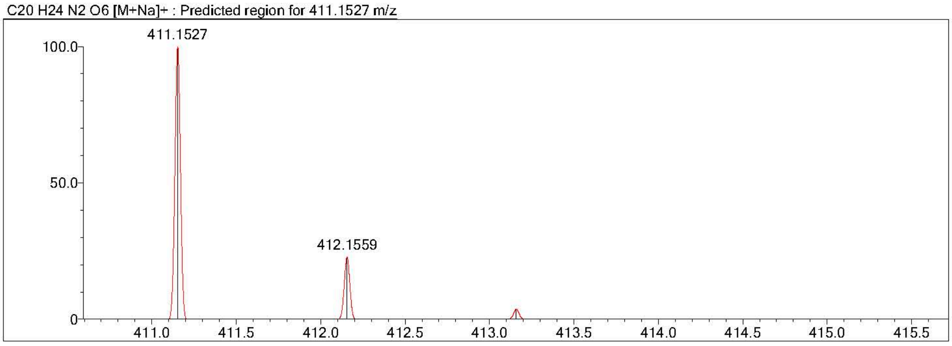

Rank	Score	Formula (M)	Ion	Meas. m/z	Pred. m/z	Df. (mDa)	Df. (ppm)	Iso	DBE
1	72.02	C20 H24 N2 O6	[M+Na]+	411.1528	411.1527	0.1	0.24	72.02	10.0

^{1}H NMR (300 MHz, $CDCl_3$) of **8e**

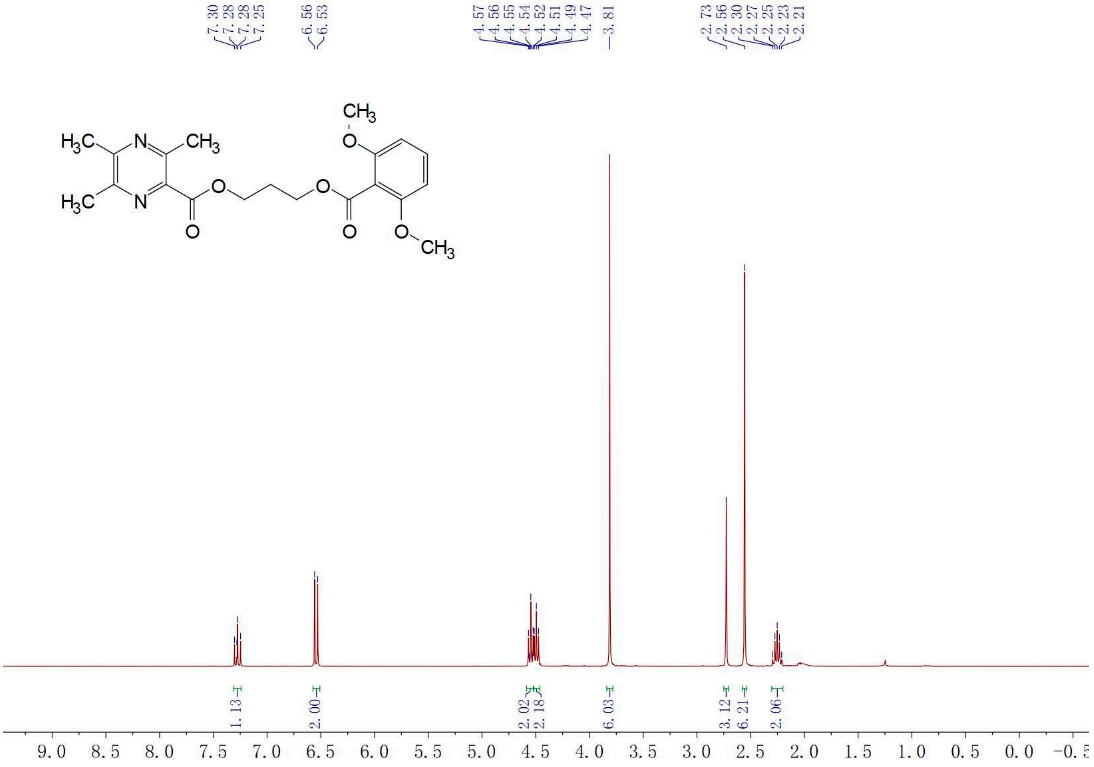

^{13}C NMR (75 MHz, $CDCl_3$) of **8e**

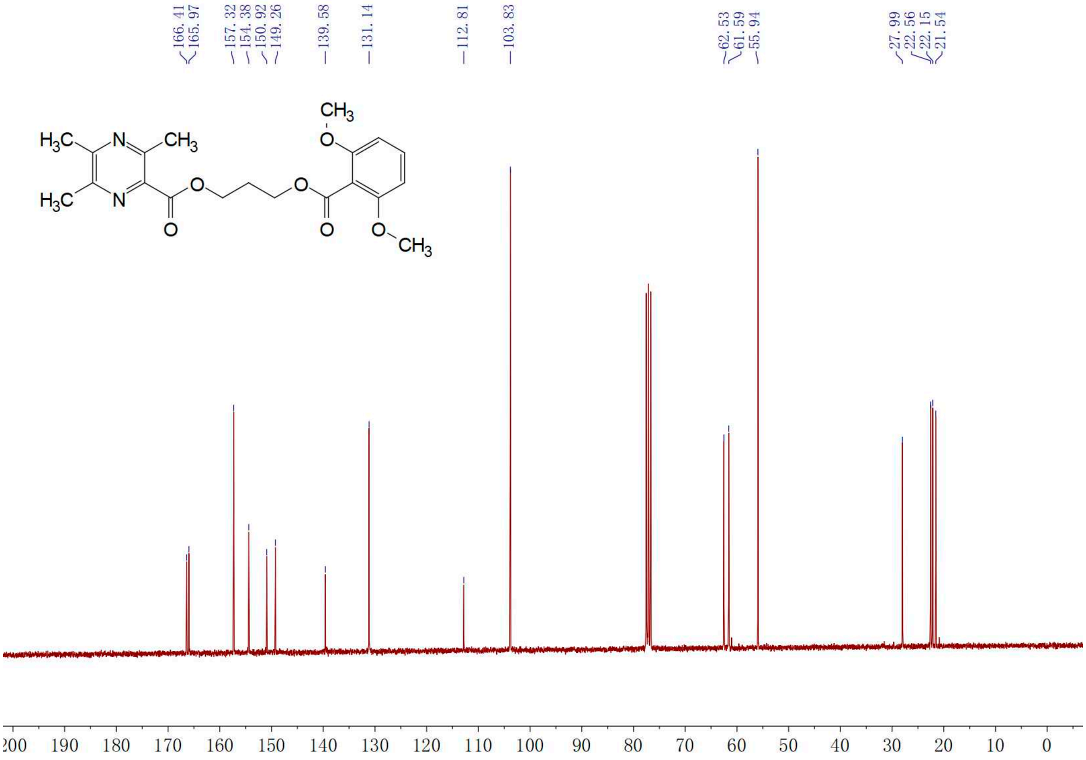

HRMS spectra of **8e**

Data File: I:\20170411-GY\20.lcd

Elmt	Val.	Min	Max	Elmt	Val.	Min	Max	Elmt	Val.	Min	Max	Elmt	Val.	Min	Max	Use Adduct
H	1	0	60	O	2	0	7	P	3	0	0	Cu	2	0	0	H
B	3	0	0	18O	2	0	0	S	2	0	0	Br	1	0	1	Na
C	4	0	50	F	1	0	1	Cl	1	0	0	I	3	0	0	
N	3	0	5	Si	4	0	0	Ni	2	0	0					

Error Margin (ppm):	20	DBE Range:	-2.0 - 1000.0	Electron Ions:	both
HC Ratio:	unlimited	Apply N Rule:	yes	Use MSn Info:	no
Max Isotopes:	all	Isotope RI (%):	1.00	Isotope Res:	10000
MSn Iso RI (%):	75.00	MSn Logic Mode:	AND	Max Results:	500

Event#: 1 MS(E+) Ret. Time : 0.987 - 1.080 -> 1.278 Scan# : 149 - 163 -> 193

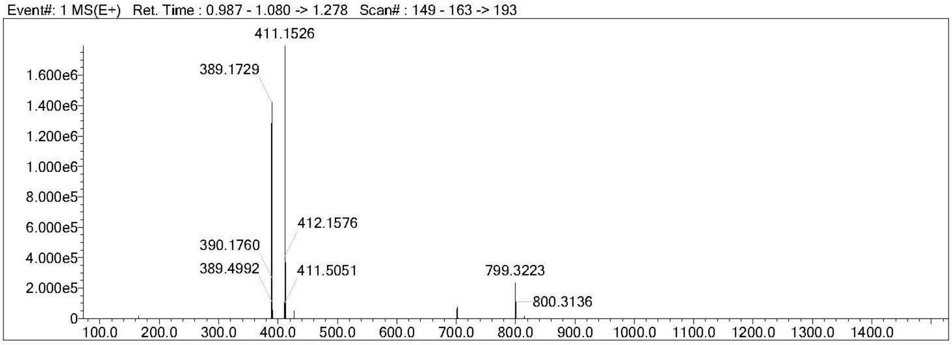

Measured region for 411.1526 m/z

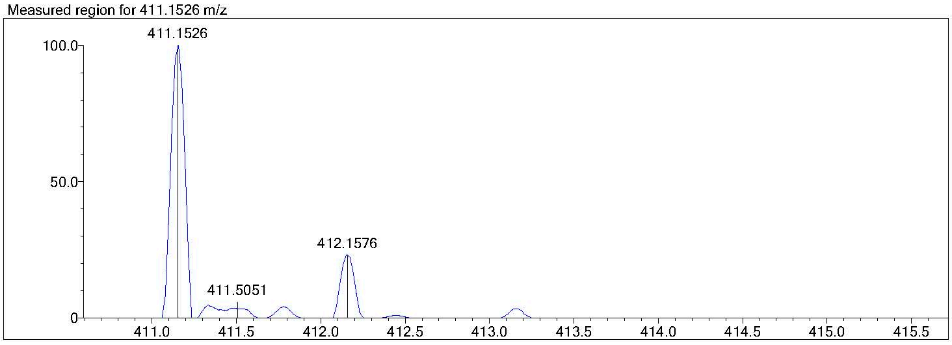

C20 H24 N2 O6 [M+Na]+ : Predicted region for 411.1527 m/z

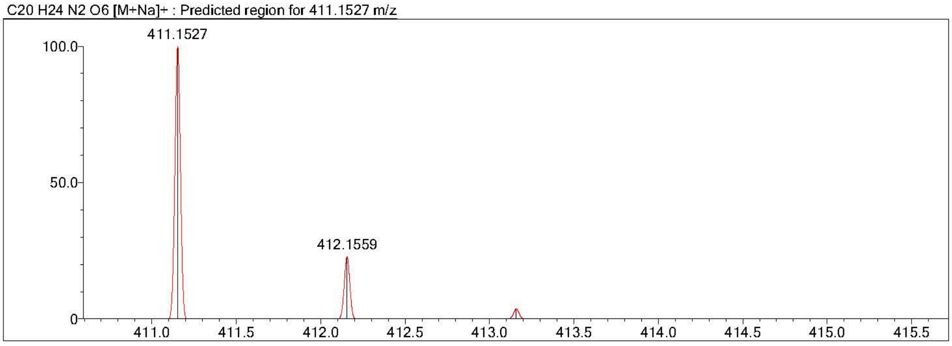

Rank	Score	Formula (M)	Ion	Meas. m/z	Pred. m/z	Df. (mDa)	Df. (ppm)	Iso	DBE
1	88.17	C20 H24 N2 O6	[M+Na]+	411.1526	411.1527	-0.1	-0.24	88.17	10.0

^{1}H NMR (300 MHz, $CDCl_3$) of **8f**

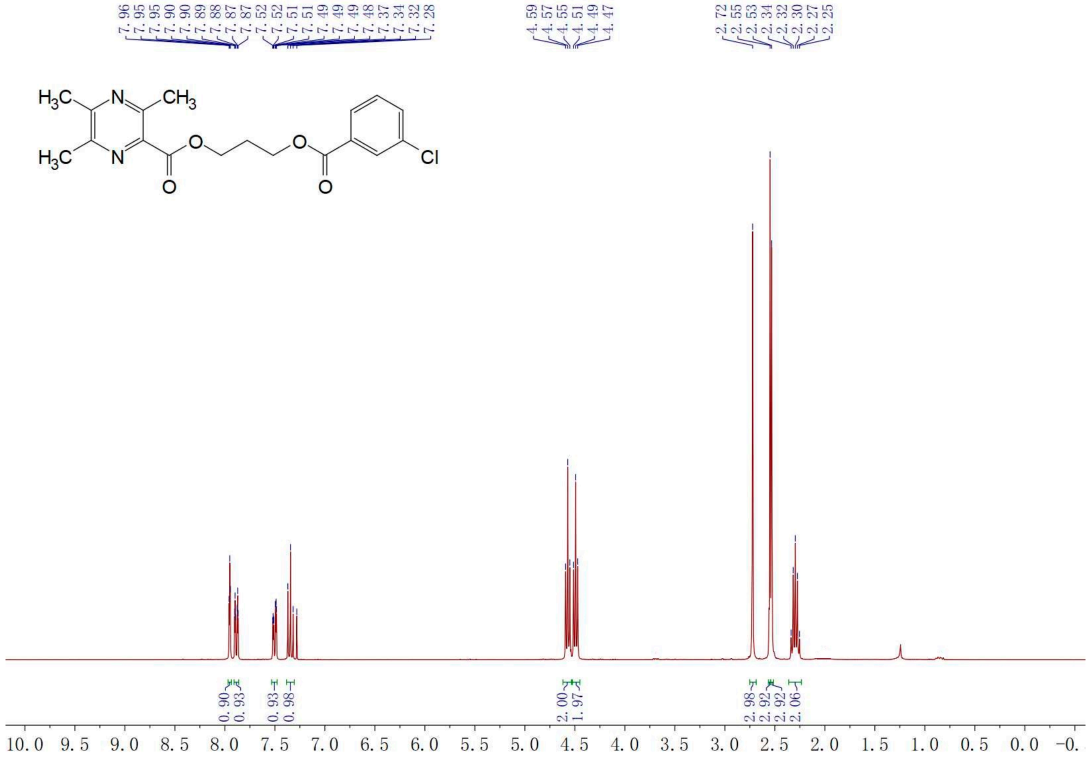

^{13}C NMR (75 MHz, $CDCl_3$) of **8f**

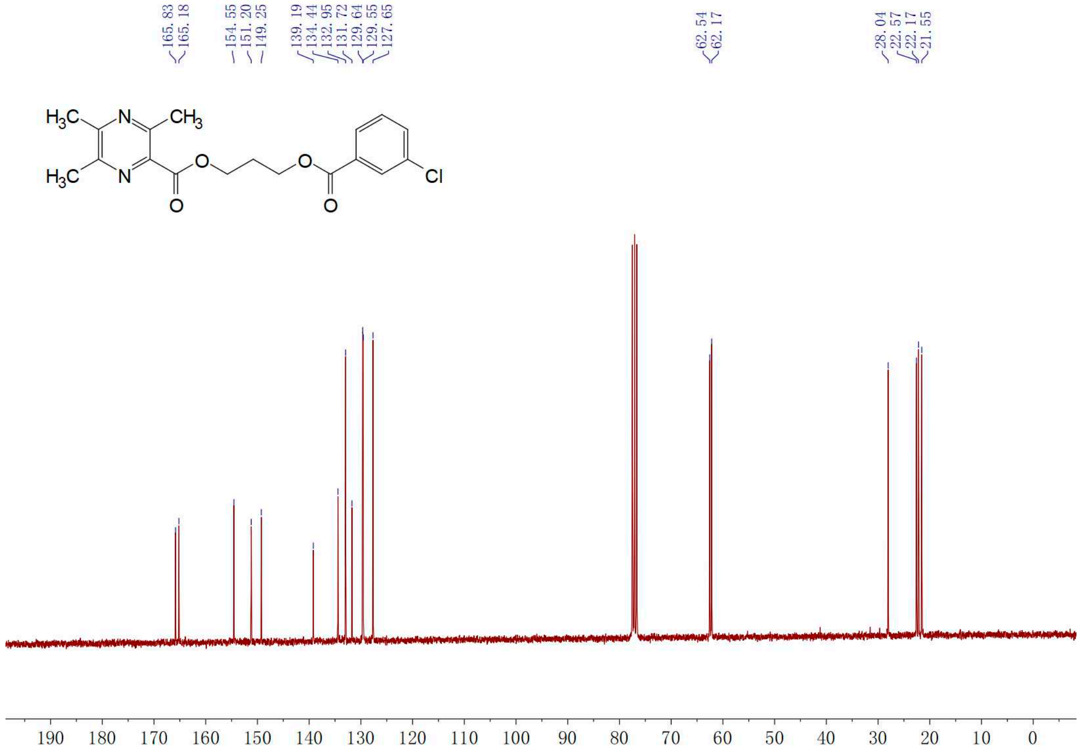

HRMS spectra of **8f**

Formula Predictor Report - 23.lcd

Data File: I:\20170411-GY\23.lcd

Elmt	Val.	Min	Max	Elmt	Val.	Min	Max	Elmt	Val.	Min	Max	Elmt	Val.	Min	Max	Use Adduct
H	1	0	60	O	2	0	7	P	3	0	0	Cu	2	0	0	H
B	3	0	0	18O	2	0	0	S	2	0	0	Br	1	0	1	Na
C	4	0	50	F	1	0	0	Cl	1	0	1	I	3	0	0	
N	3	0	5	Si	4	0	0	Ni	2	0	0					

Error Margin (ppm):	20	DBE Range:	-2.0 - 1000.0	Electron Ions:	both
HC Ratio:	unlimited	Apply N Rule:	yes	Use MSn Info:	no
Max Isotopes:	all	Isotope RI (%):	1.00	Isotope Res:	10000
MSn Iso RI (%):	75.00	MSn Logic Mode:	AND	Max Results:	500

Event#: 1 MS(E+) Ret. Time : 1.107 Scan# : 167

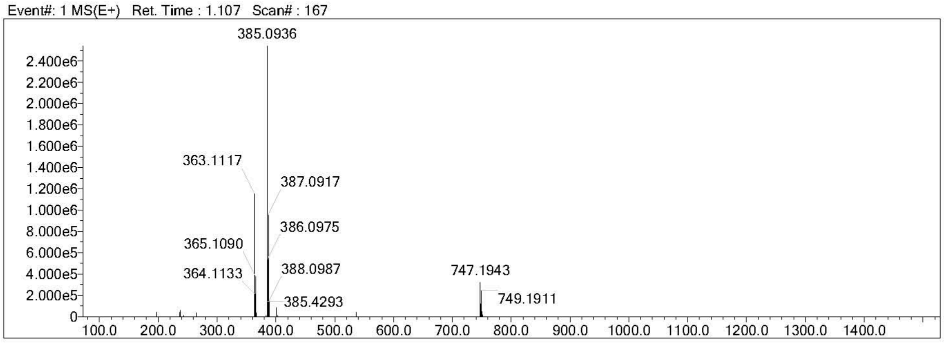

Measured region for 385.0936 m/z

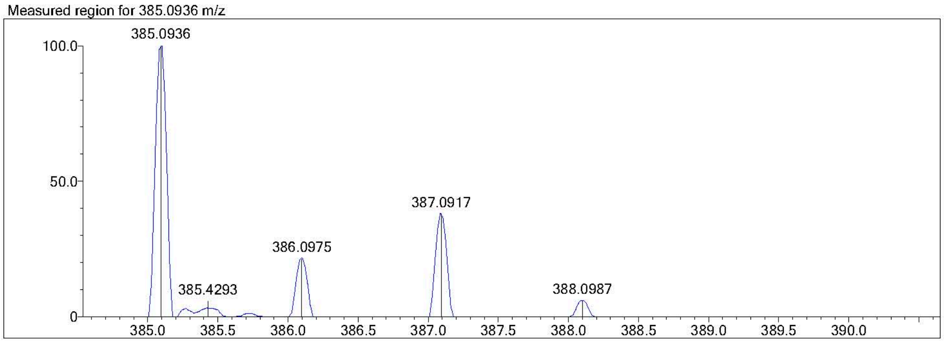

C18 H19 N2 O4 Cl [M+Na]+ : Predicted region for 385.0926 m/z

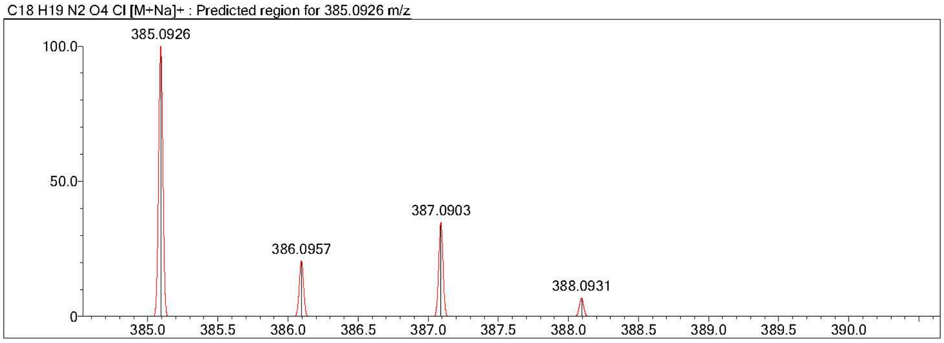

Rank	Score	Formula (M)	Ion	Meas. m/z	Pred. m/z	Df. (mDa)	Df. (ppm)	Iso	DBE
2	83.09	C18 H19 N2 O4 Cl	[M+Na]+	385.0936	385.0926	1.0	2.60	86.56	10.0

^{1}H NMR (400 MHz, DMSO-d6) of **8g**

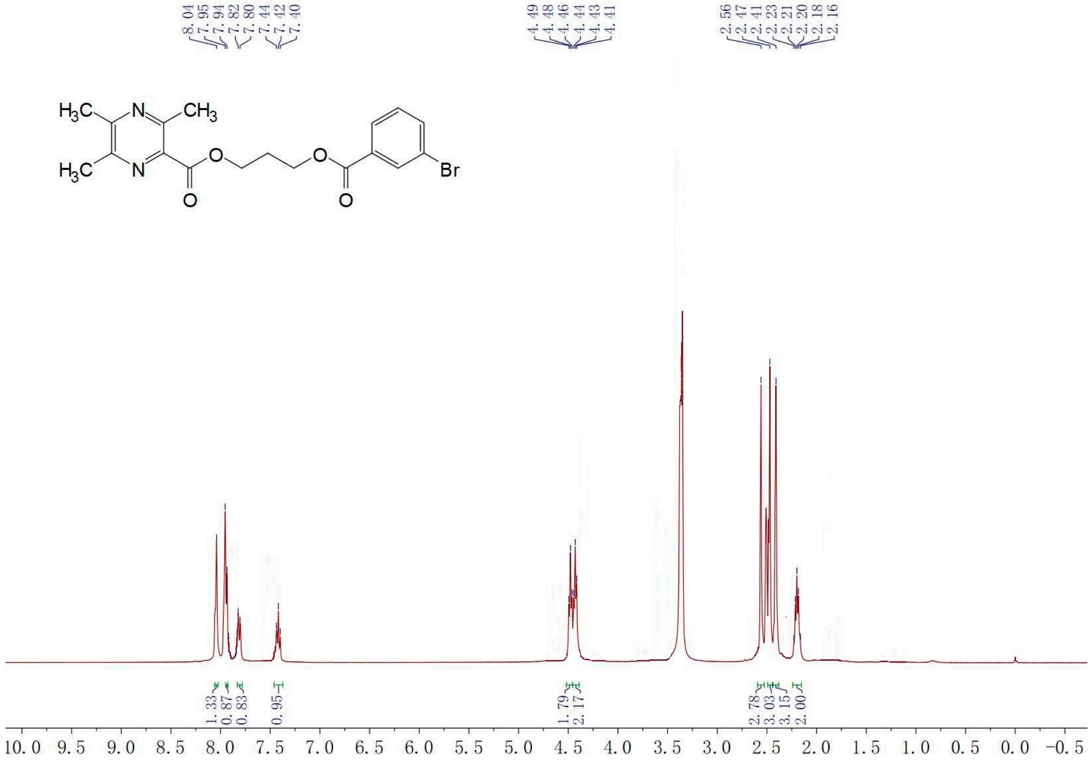

^{13}C NMR (100 MHz, DMSO-d6) of **8g**

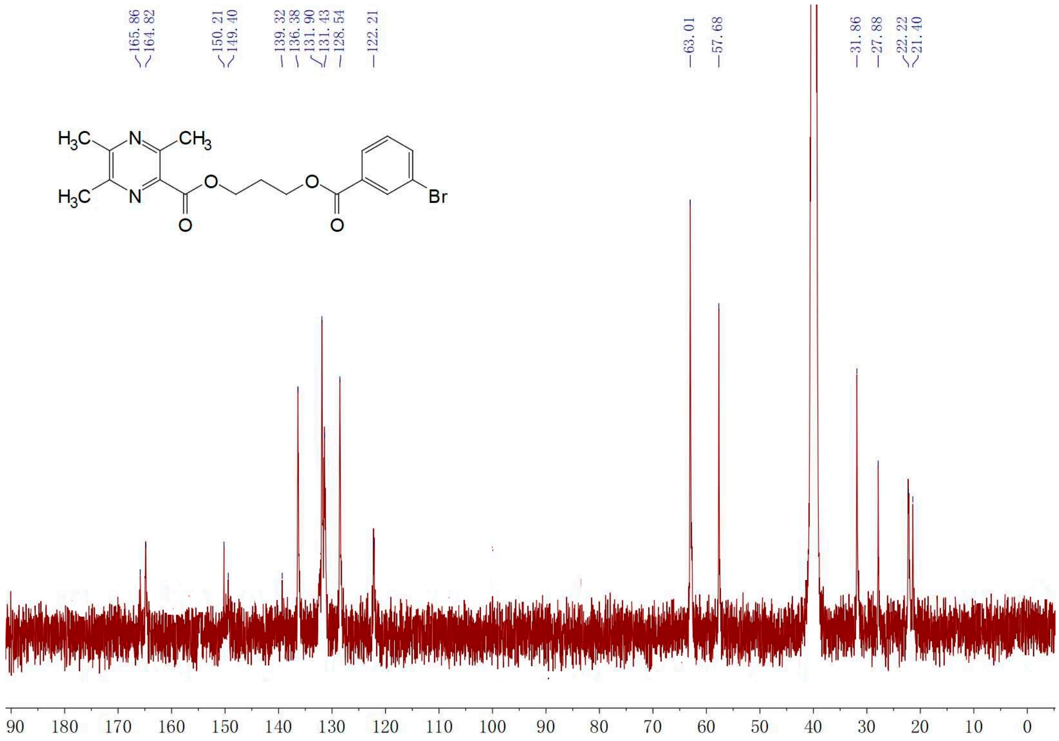

HRMS spectra of **8g**

Data File: I:\20170411-GY\1.lcd

Elmt	Val.	Min	Max	Elmt	Val.	Min	Max	Elmt	Val.	Min	Max	Elmt	Val.	Min	Max	Use Adduct
H	1	0	60	O	2	0	5	P	3	0	0	Cu	2	0	0	H
B	3	0	1	18O	2	0	0	S	2	0	0	Br	1	0	1	Na
C	4	0	50	F	1	0	0	Cl	1	0	0	I	3	0	0	
N	3	0	5	Si	4	0	0	Ni	2	0	0					

Error Margin (ppm):	100	DBE Range:	-2.0 - 1000.0	Electron Ions:	both
HC Ratio:	unlimited	Apply N Rule:	yes	Use MSn Info:	no
Max Isotopes:	all	Isotope RI (%):	1.00	Isotope Res:	10000
MSn Iso RI (%):	75.00	MSn Logic Mode:	AND	Max Results:	500

Event#: 1 MS(E+) Ret. Time : 1.240 - 1.507 -> 1.793 Scan# : 187 - 227 -> 269

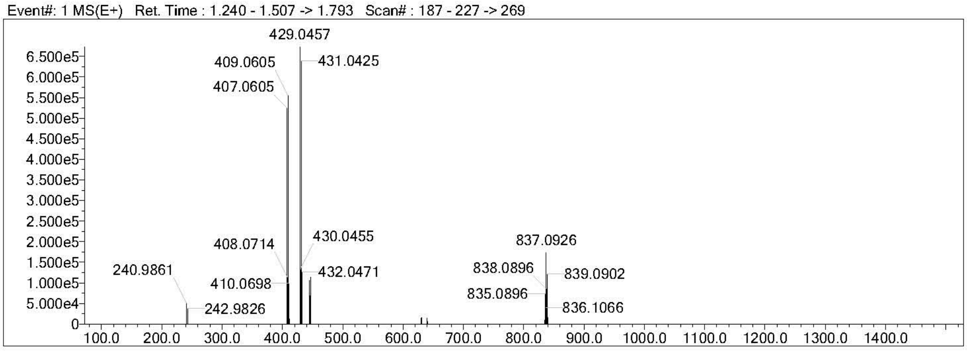

Measured region for 407.0605 m/z

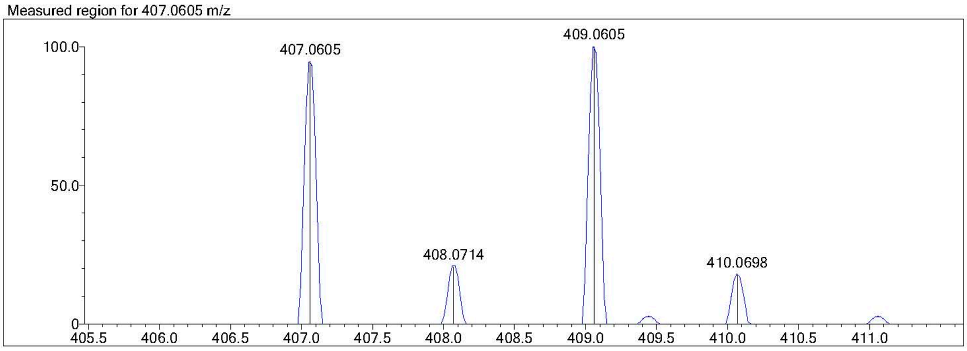

C18 H19 N2 O4 Br [M+H]+ : Predicted region for 407.0601 m/z

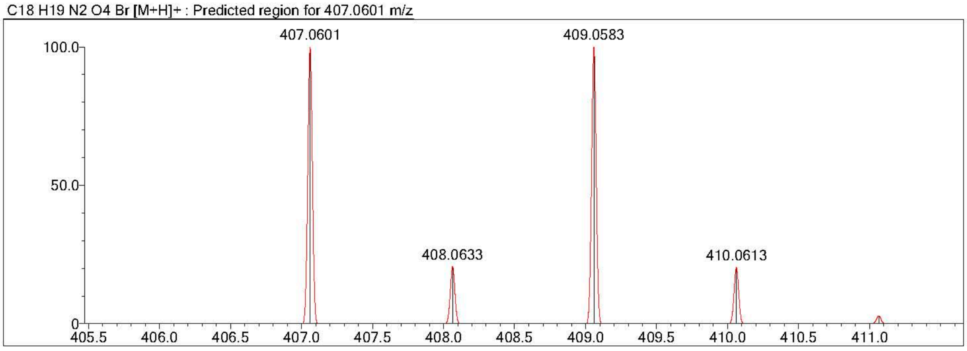

Rank	Score	Formula (M)	Ion	Meas. m/z	Pred. m/z	Df. (mDa)	Df. (ppm)	Iso	DBE
2	72.39	C18 H19 N2 O4 Br	[M+H]+	407.0605	407.0601	0.4	0.98	72.39	10.0

^{1}H NMR (400 MHz, DMSO-d6) of **8h**

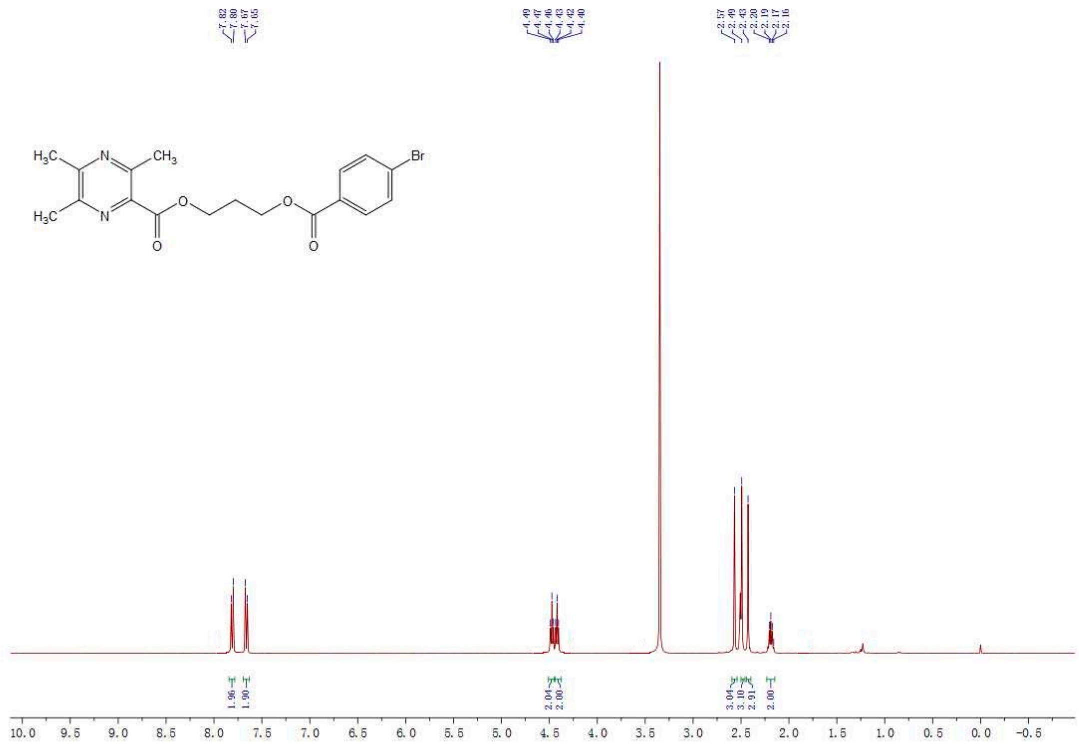

^{13}C NMR (100 MHz, DMSO-d6) of **8h**

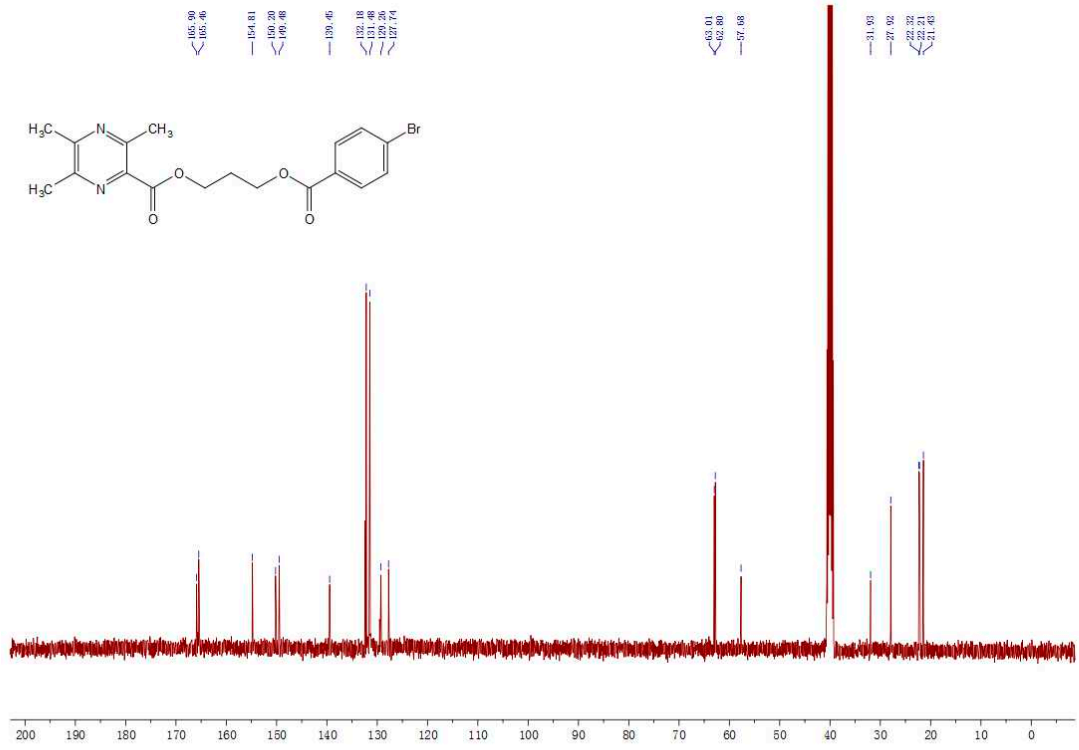

HRMS spectra of **8h**

Formula Predictor Report - 2.lcd

Page 1 of 1

Data File: I:\20170411-GY\2.lcd

Elmt	Val.	Min	Max	Elmt	Val.	Min	Max	Elmt	Val.	Min	Max	Elmt	Val.	Min	Max	Use Adduct
H	1	0	60	O	2	0	5	P	3	0	0	Cu	2	0	0	H
B	3	0	1	18O	2	0	0	S	2	0	0	Br	1	0	1	Na
C	4	0	50	F	1	0	0	Cl	1	0	0	I	3	0	0	
N	3	0	5	Si	4	0	0	Ni	2	0	0					

Error Margin (ppm):	100	DBE Range:	-2.0 - 1000.0	Electron Ions:	both
HC Ratio:	unlimited	Apply N Rule:	yes	Use MSn Info:	no
Max Isotopes:	all	Isotope RI (%):	1.00	Isotope Res:	10000
MSn Iso RI (%):	75.00	MSn Logic Mode:	AND	Max Results:	500

Event#: 1 MS(E+) Ret. Time : 1.133 Scan# : 171

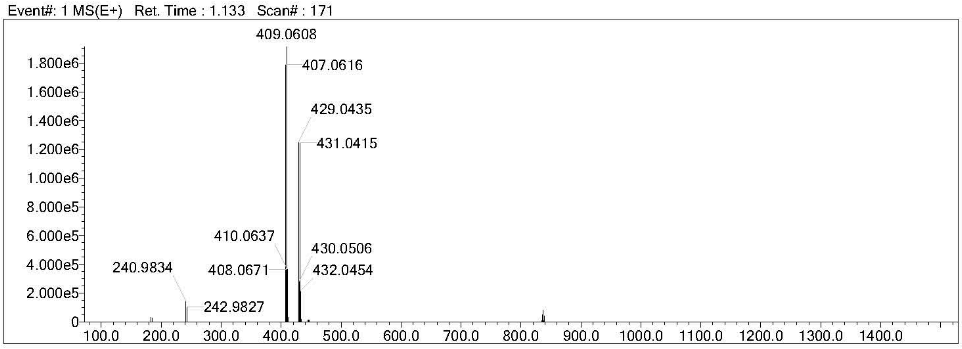

Measured region for 407.0616 m/z

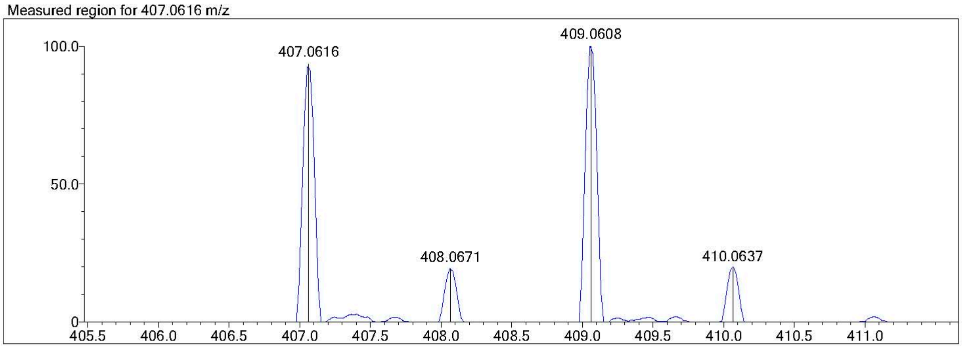

C18 H19 N2 O4 Br [M+H]+ : Predicted region for 407.0601 m/z

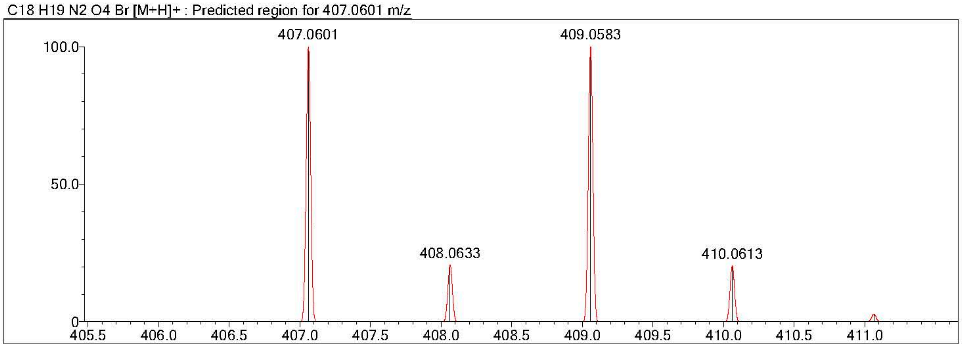

Rank	Score	Formula (M)	Ion	Meas. m/z	Pred. m/z	Df. (mDa)	Df. (ppm)	Iso	DBE
2	68.13	C18 H19 N2 O4 Br	[M+H]+	407.0616	407.0601	1.5	3.68	73.02	10.0

^{1}H NMR (400 MHz, DMSO-d6) of **8i**

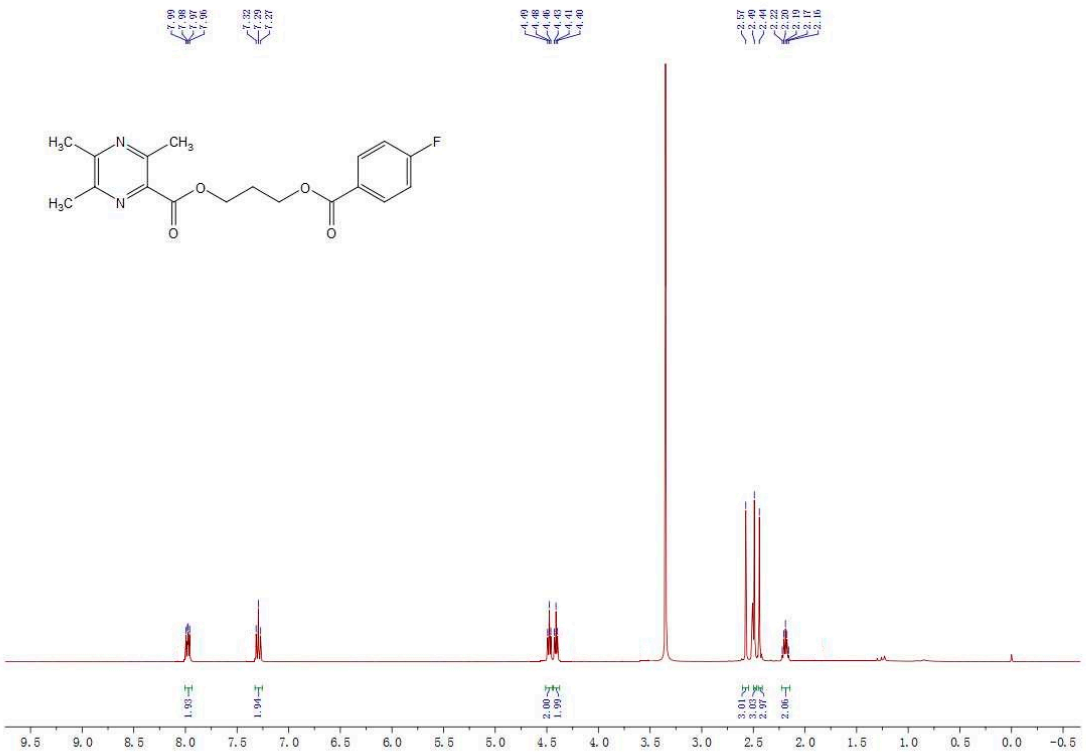

^{13}C NMR (100 MHz, DMSO-d6) of **8i**

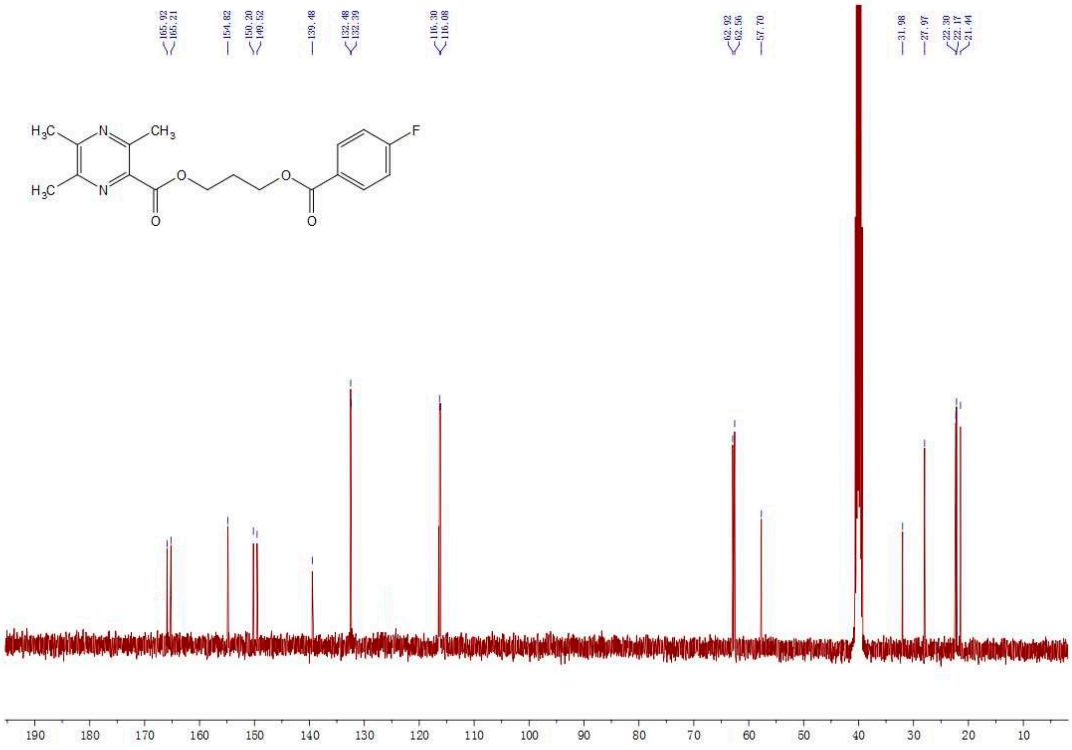

HRMS spectra of **8i**

Formula Predictor Report - 3.lcd

Data File: I:\20170411-GY\3.lcd

Elmt	Val.	Min	Max	Elmt	Val.	Min	Max	Elmt	Val.	Min	Max	Elmt	Val.	Min	Max	Use Adduct
H	1	0	60	O	2	0	5	P	3	0	0	Cu	2	0	0	H
B	3	0	0	18O	2	0	0	S	2	0	0	Br	1	0	1	Na
C	4	0	50	F	1	0	1	Cl	1	0	0	I	3	0	0	
N	3	0	5	Si	4	0	0	Ni	2	0	0					

Error Margin (ppm):	100	DBE Range:	-2.0 - 1000.0	Electron Ions:	both
HC Ratio:	unlimited	Apply N Rule:	yes	Use MSn Info:	no
Max Isotopes:	all	Isotope RI (%):	1.00	Isotope Res:	10000
MSn Iso RI (%):	75.00	MSn Logic Mode:	AND	Max Results:	500

Event#: 1 MS(E+) Ret. Time : 1.013 - 1.227 -> 1.588 Scan# : 153 - 185 -> 239

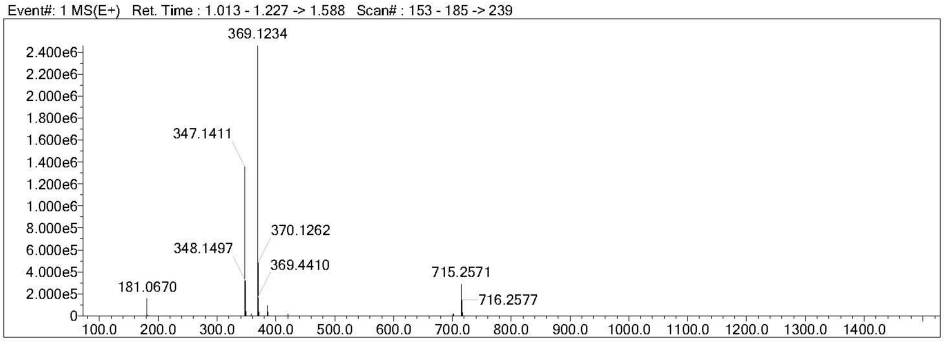

Measured region for 347.1411 m/z

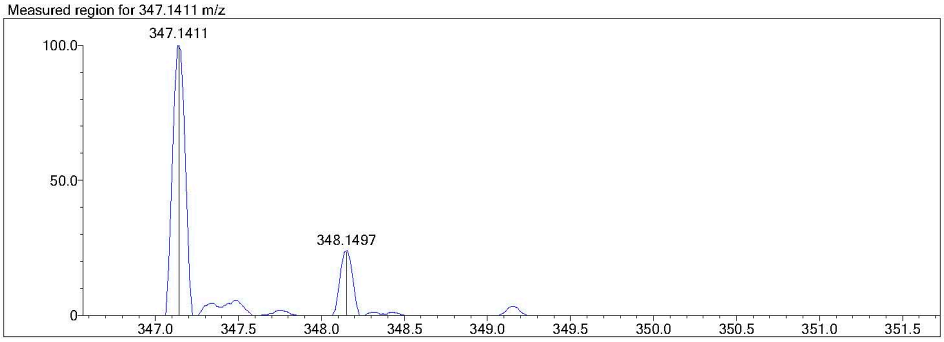

C18 H19 N2 O4 F [M+H]+ : Predicted region for 347.1402 m/z

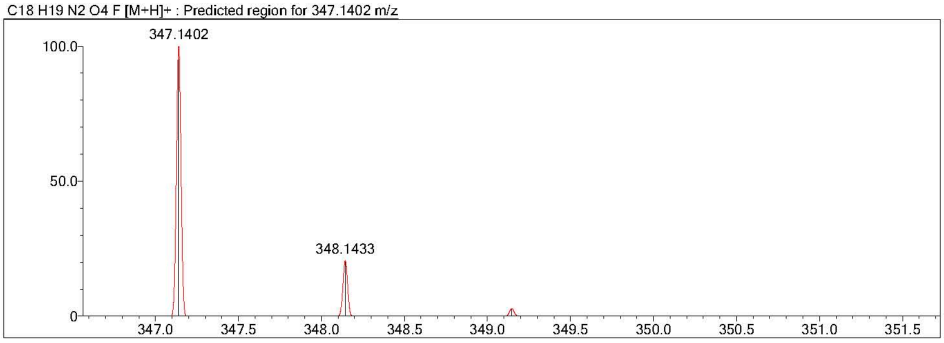

Rank	Score	Formula (M)	Ion	Meas. m/z	Pred. m/z	Df. (mDa)	Df. (ppm)	Iso	DBE
3	75.67	C18 H19 N2 O4 F	[M+H]+	347.1411	347.1402	0.9	2.59	78.80	10.0

^{1}H NMR (400 MHz, DMSO-d6) of **8j**

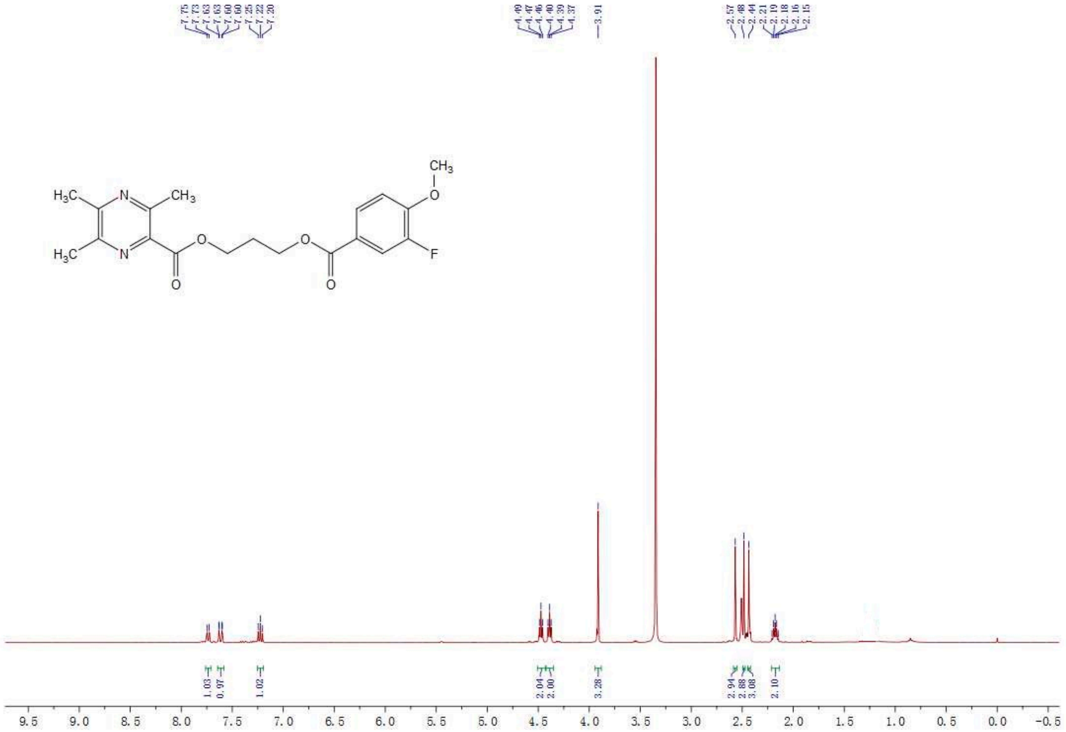

^{13}C NMR (100 MHz, DMSO-d6) of **8j**

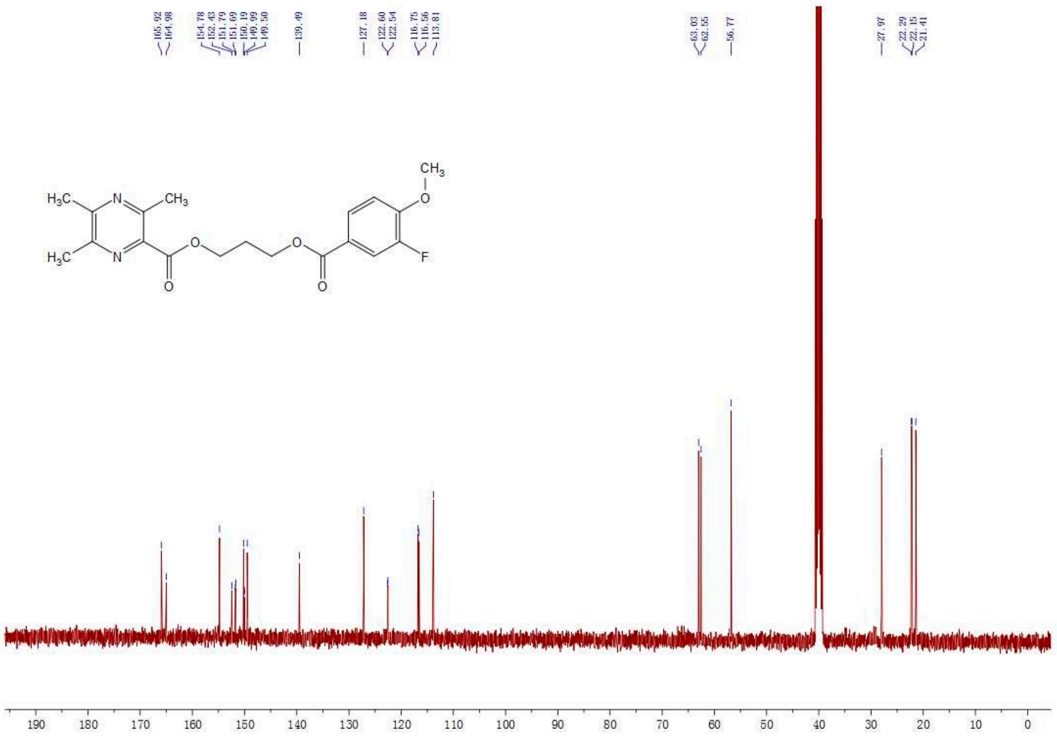

HRMS spectra of **8j**

Data File: I:\20170411-GY\8.lcd

Elmt	Val.	Min	Max	Elmt	Val.	Min	Max	Elmt	Val.	Min	Max	Elmt	Val.	Min	Max	Use Adduct
H	1	0	60	O	2	0	6	P	3	0	0	Cu	2	0	0	H
B	3	0	0	18O	2	0	0	S	2	0	0	Br	1	0	1	Na
C	4	0	50	F	1	0	1	Cl	1	0	0	I	3	0	0	
N	3	0	5	Si	4	0	0	Ni	2	0	0					

Error Margin (ppm): 20
HC Ratio: unlimited
Max Isotopes: all
MSn Iso RI (%): 75.00

DBE Range: -2.0 - 1000.0
Apply N Rule: yes
Isotope RI (%): 1.00
MSn Logic Mode: AND

Electron Ions: both
Use MSn Info: no
Isotope Res: 10000
Max Results: 500

Event#: 1 MS(E+) Ret. Time : 1.107 - 1.160 -> 1.375 Scan# : 167 - 175 -> 207

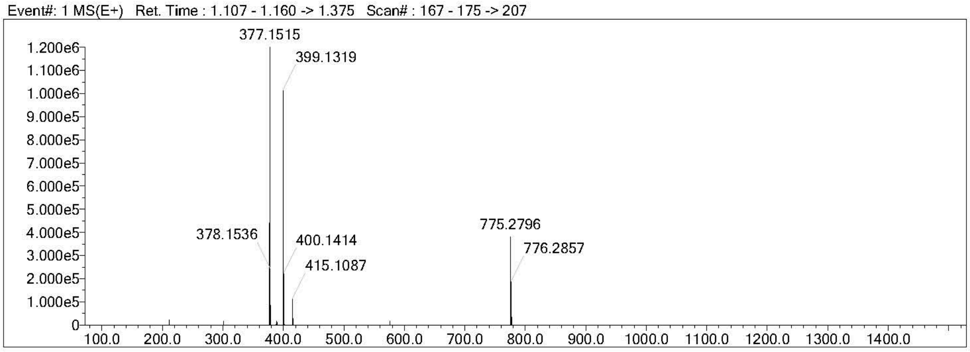

Measured region for 377.1515 m/z

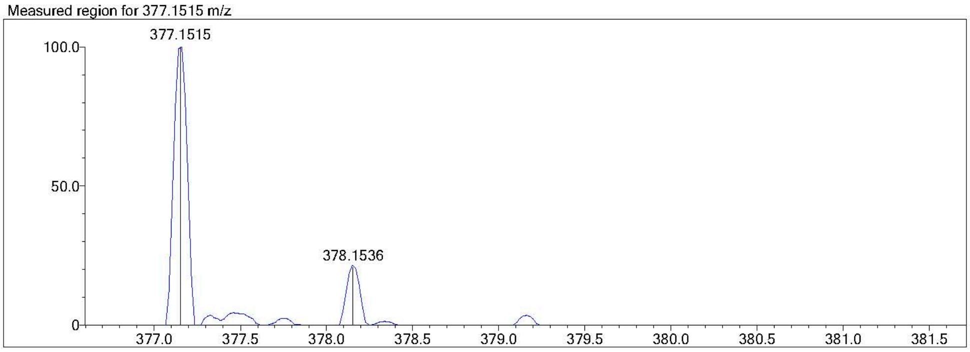

C19 H21 N2 O5 F [M+H]+ : Predicted region for 377.1507 m/z

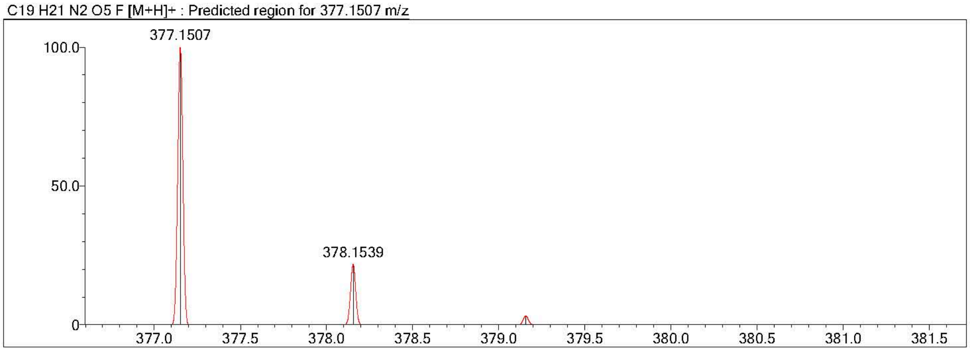

Rank	Score	Formula (M)	Ion	Meas. m/z	Pred. m/z	Df. (mDa)	Df. (ppm)	Iso	DBE
1	84.47	C19 H21 N2 O5 F	[M+H]+	377.1515	377.1507	0.8	2.12	86.91	10.0

^{1}H NMR (400 MHz, DMSO-d6) of **8k**

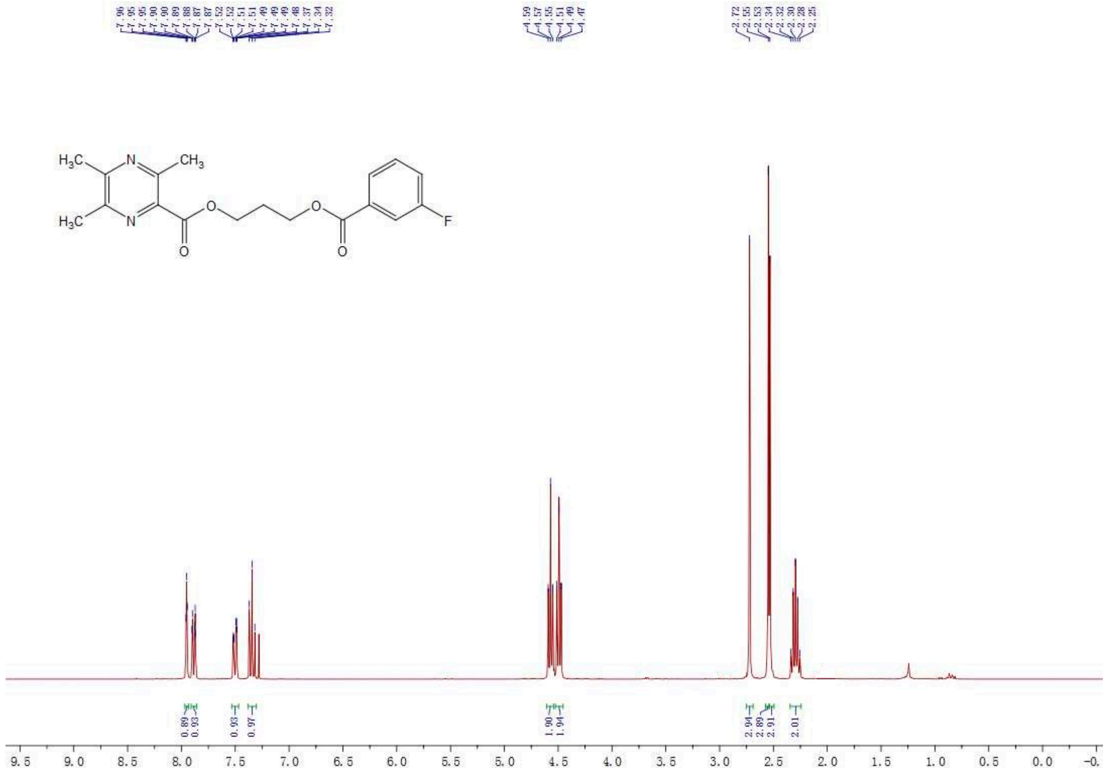

^{13}C NMR (100 MHz, DMSO-d6) of **8k**

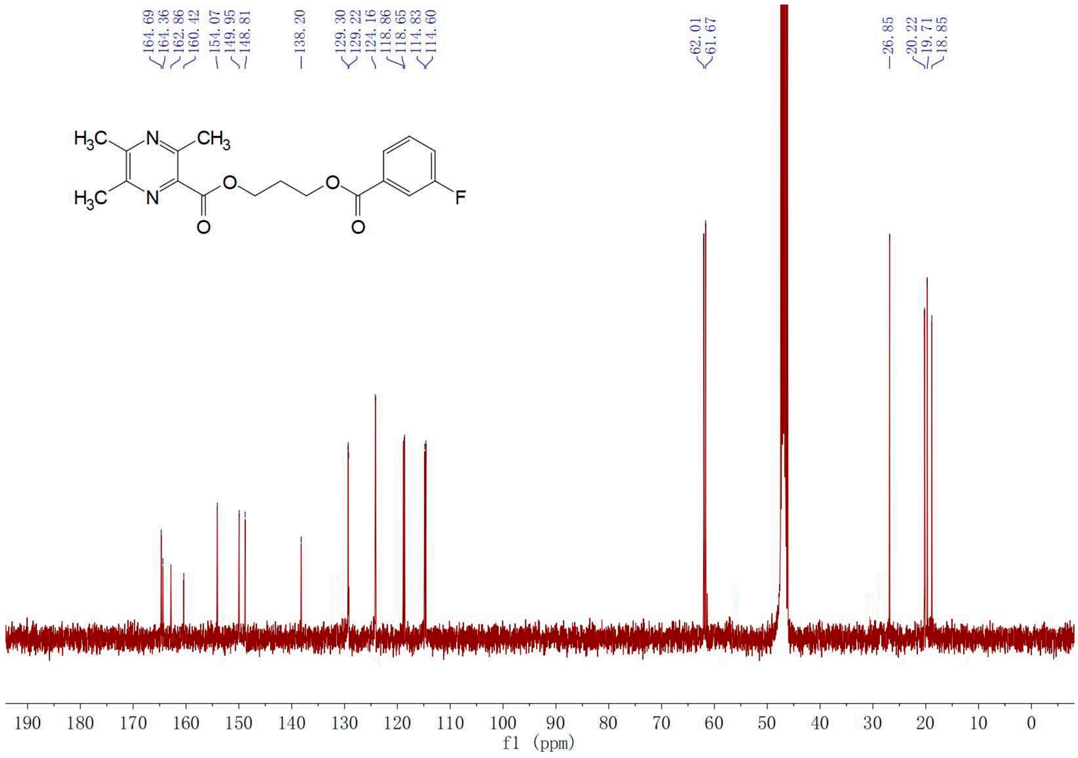

HRMS spectra of **8k**

Formula Predictor Report - 12.lcd   Page 1 of 1

Data File: I:\20170411-GY\12.lcd

Elmt	Val.	Min	Max	Elmt	Val.	Min	Max	Elmt	Val.	Min	Max	Elmt	Val.	Min	Max	Use Adduct
H	1	0	60	O	2	0	6	P	3	0	0	Cu	2	0	0	H
B	3	0	0	18O	2	0	0	S	2	0	0	Br	1	0	1	Na
C	4	0	50	F	1	0	1	Cl	1	0	0	I	3	0	0	
N	3	0	5	Si	4	0	0	Ni	2	0	0					

Error Margin (ppm):	20	DBE Range:	-2.0 - 1000.0	Electron Ions:	both
HC Ratio:	unlimited	Apply N Rule:	yes	Use MSn Info:	no
Max Isotopes:	all	Isotope RI (%):	1.00	Isotope Res:	10000
MSn Iso RI (%):	75.00	MSn Logic Mode:	AND	Max Results:	500

Event#: 1 MS(E+)   Ret. Time : 1.080   Scan# : 163

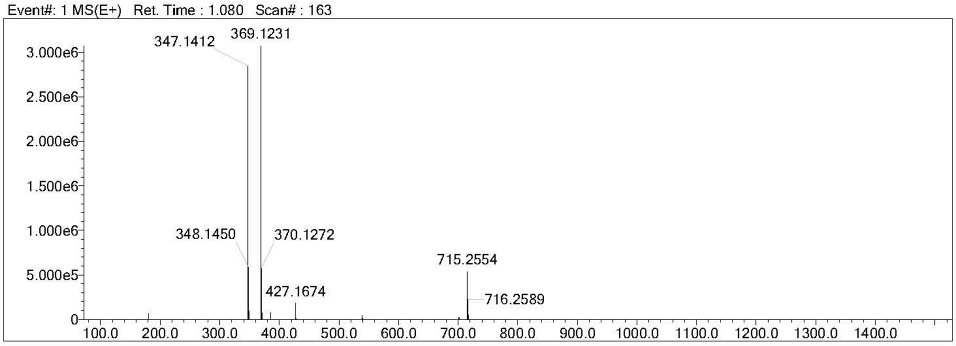

Measured region for 369.1231 m/z

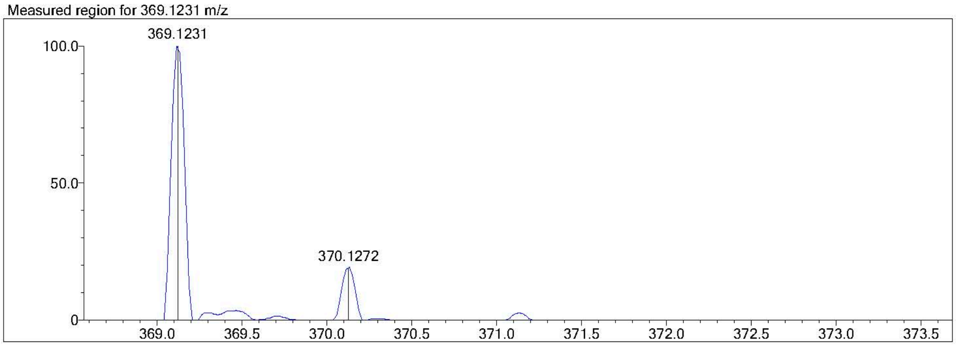

C18 H19 N2 O4 F [M+Na]+ : Predicted region for 369.1221 m/z

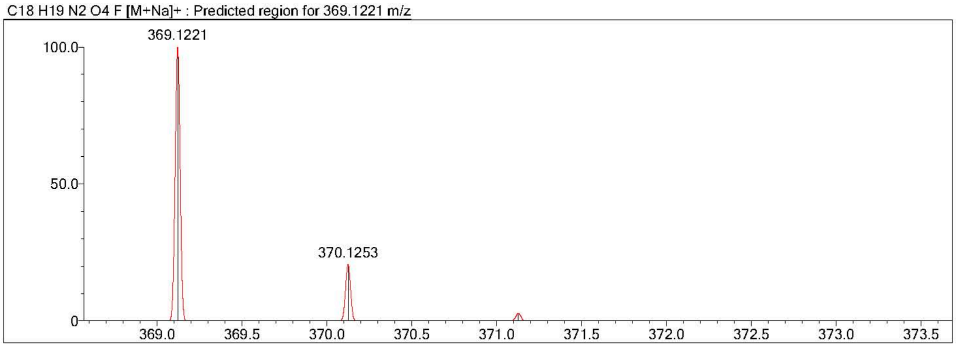

Rank	Score	Formula (M)	Ion	Meas. m/z	Pred. m/z	Df. (mDa)	Df. (ppm)	Iso	DBE
1	83.70	C18 H19 N2 O4 F	[M+Na]+	369.1231	369.1221	1.0	2.71	87.43	10.0

^{1}H NMR (300 MHz, $CDCl_3$) of **8l**

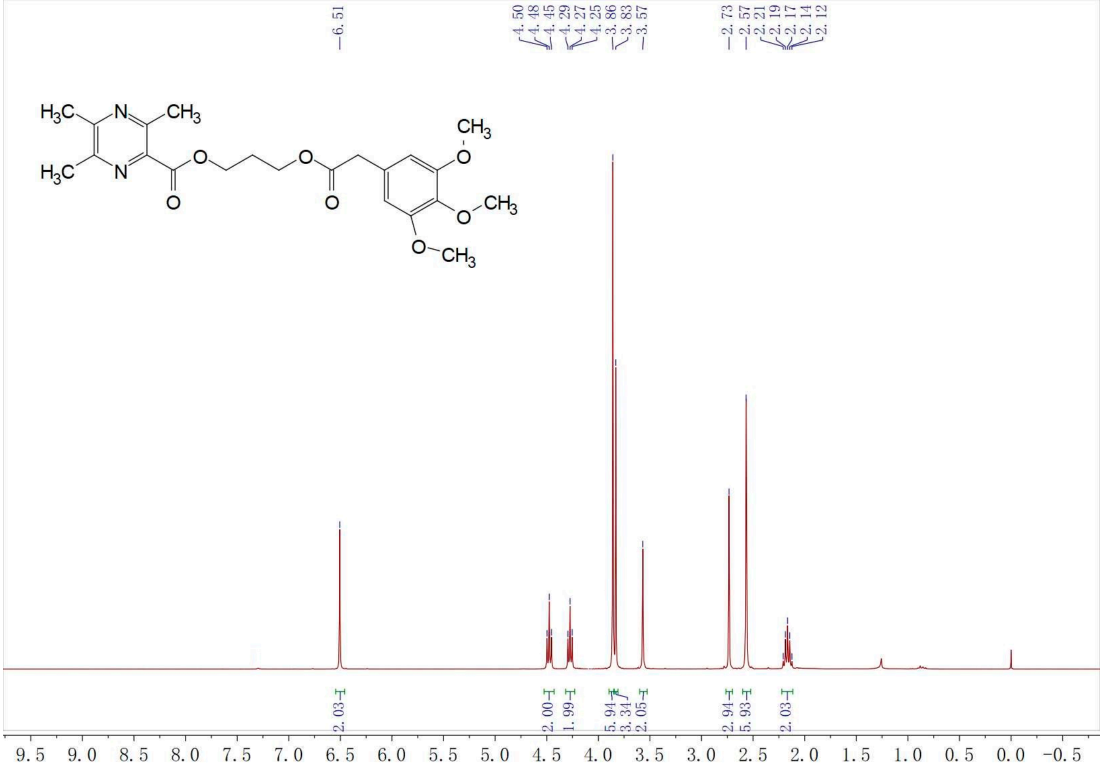

^{13}C NMR (75 MHz, $CDCl_3$) of **8l**

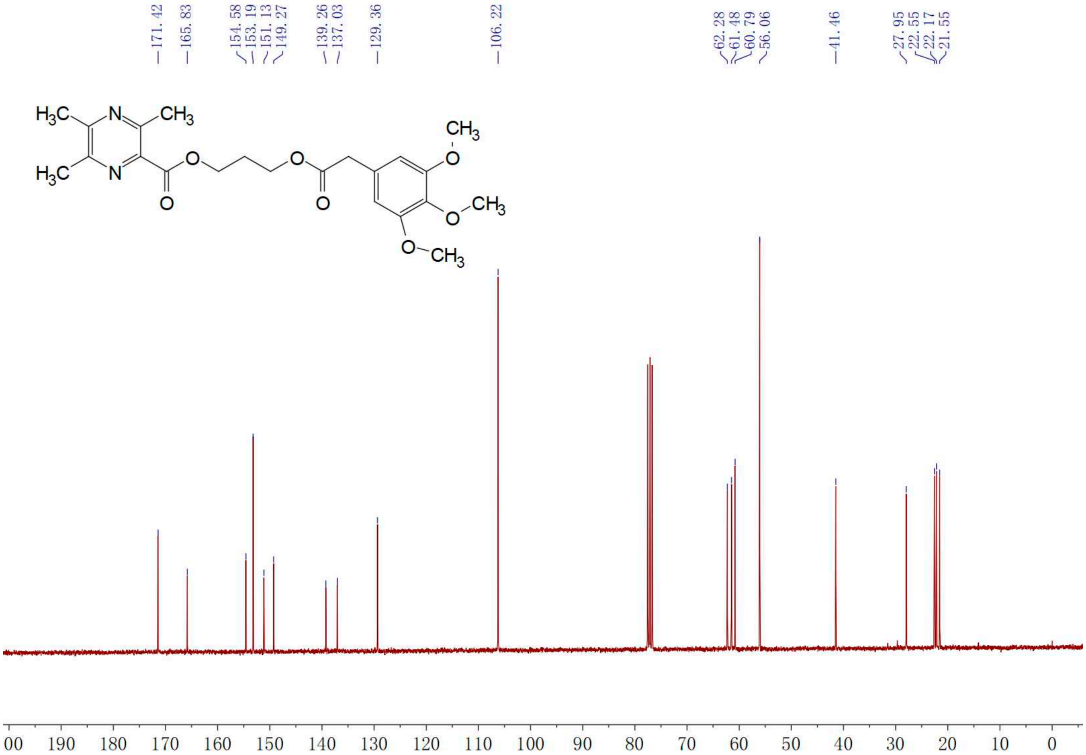

HRMS spectra of **8l**

Formula Predictor Report - 19.lcd

Data File: I:\20170411-GY\19.lcd

Elmt	Val.	Min	Max	Elmt	Val.	Min	Max	Elmt	Val.	Min	Max	Elmt	Val.	Min	Max	Use Adduct
H	1	0	60	O	2	0	7	P	3	0	0	Cu	2	0	0	H
B	3	0	0	18O	2	0	0	S	2	0	0	Br	1	0	1	Na
C	4	0	50	F	1	0	1	Cl	1	0	0	I	3	0	0	
N	3	0	5	Si	4	0	0	Ni	2	0	0					

Error Margin (ppm):	20	DBE Range:	-2.0 - 1000.0	Electron Ions:	both
HC Ratio:	unlimited	Apply N Rule:	yes	Use MSn Info:	no
Max Isotopes:	all	Isotope RI (%):	1.00	Isotope Res:	10000
MSn Iso RI (%):	75.00	MSn Logic Mode:	AND	Max Results:	500

Event#: 1 MS(E+) Ret. Time : 1.147 - 1.187 -> 1.367 Scan# : 173 - 179 -> 205

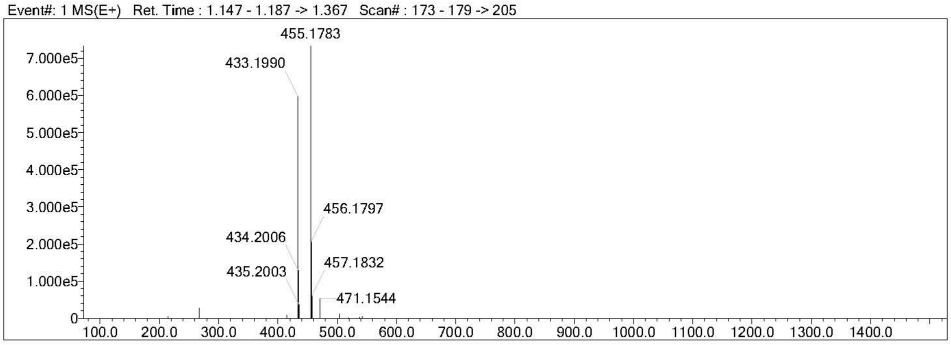

Measured region for 455.1783 m/z

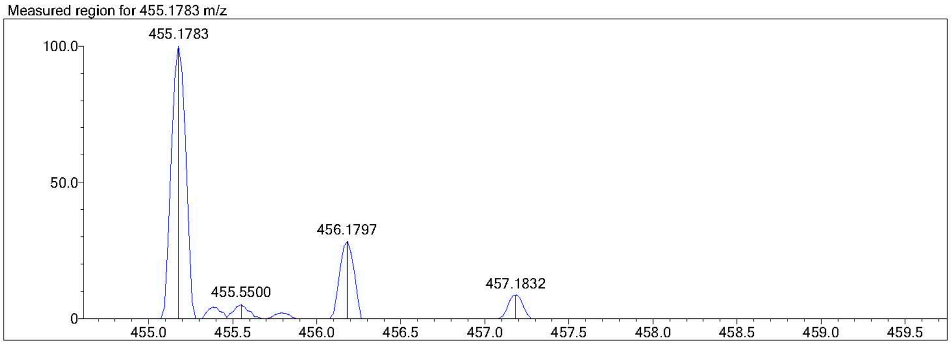

C22 H28 N2 O7 [M+Na]+ : Predicted region for 455.1789 m/z

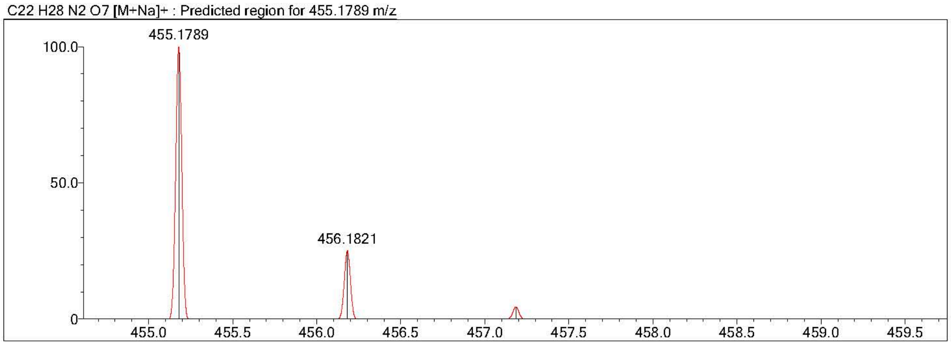

Rank	Score	Formula (M)	Ion	Meas. m/z	Pred. m/z	Df. (mDa)	Df. (ppm)	Iso	DBE
3	69.47	C22 H28 N2 O7	[M+Na]+	455.1783	455.1789	-0.6	-1.32	70.03	10.0

^{1}H NMR (400 MHz, DMSO-d6) of **8m**

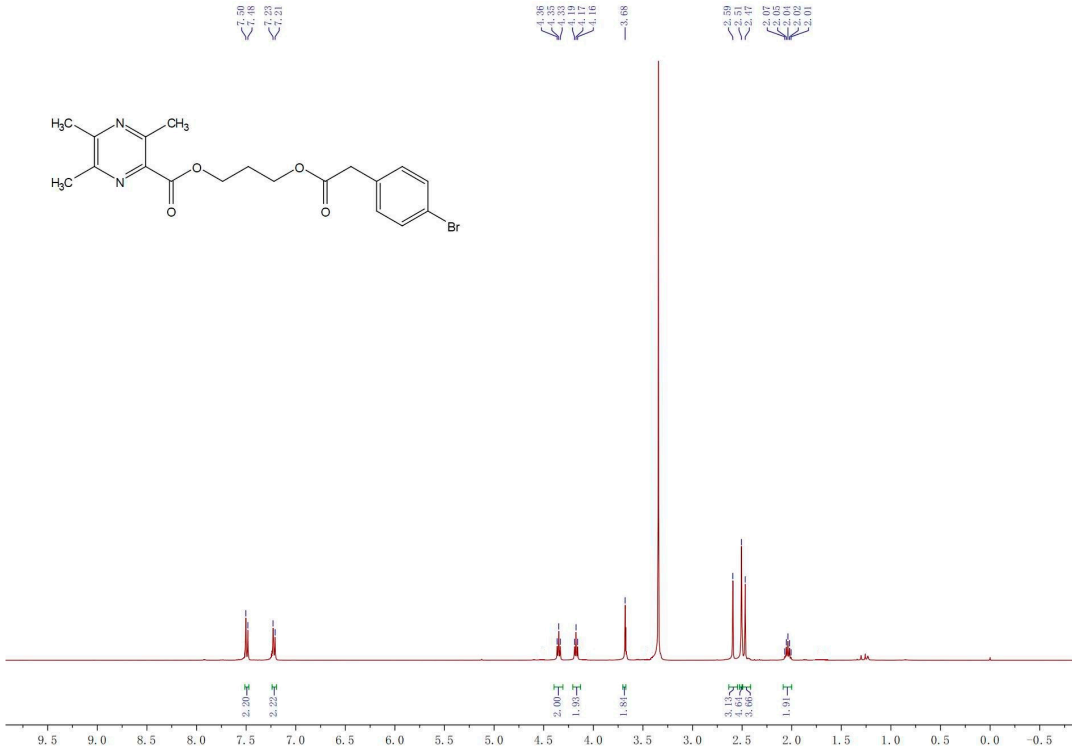

^{13}C NMR (100 MHz, DMSO-d6) of **8m**

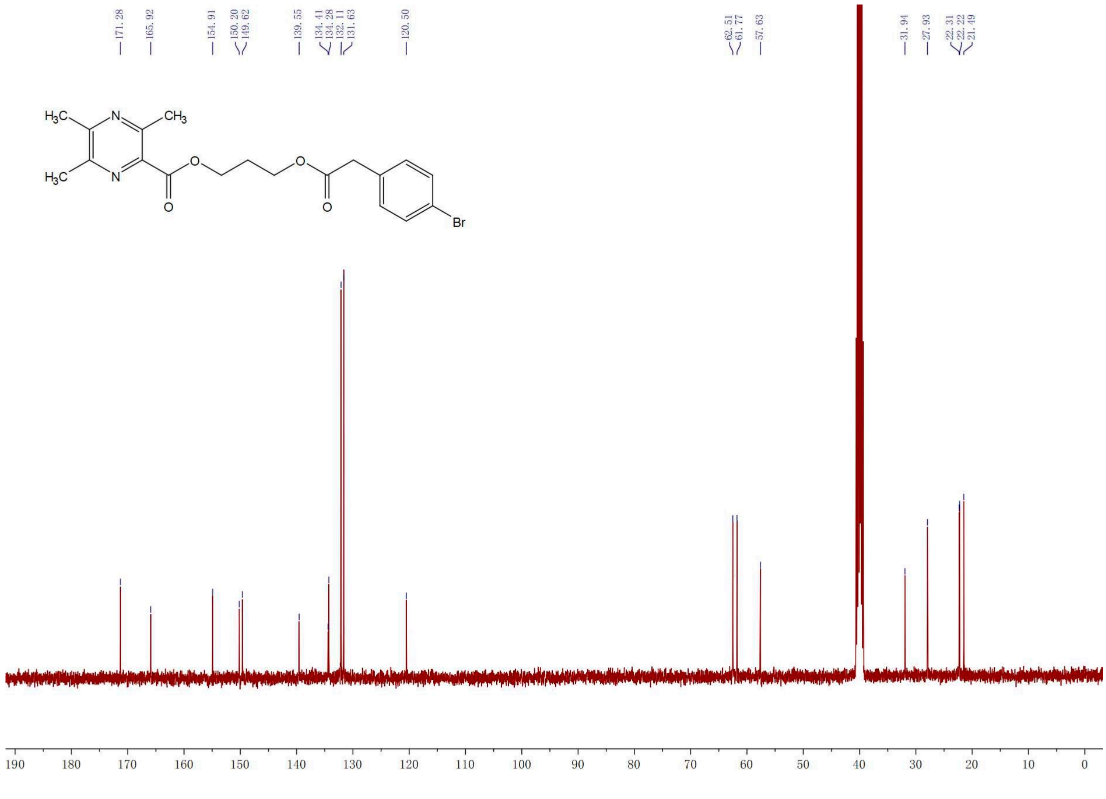

HRMS spectra of **8m**

Formula Predictor Report - 6.lcd

Data File: I:\20170411-GY\6.lcd

Elmt	Val.	Min	Max	Elmt	Val.	Min	Max	Elmt	Val.	Min	Max	Elmt	Val.	Min	Max	Use Adduct
H	1	0	60	O	2	0	6	P	3	0	0	Cu	2	0	0	H
B	3	0	0	18O	2	0	0	S	2	0	0	Br	1	0	1	Na
C	4	0	50	F	1	0	1	Cl	1	0	0	I	3	0	0	
N	3	0	5	Si	4	0	0	Ni	2	0	0					

Error Margin (ppm):	20	DBE Range:	-2.0 - 1000.0	Electron Ions:	both
HC Ratio:	unlimited	Apply N Rule:	yes	Use MSn Info:	no
Max Isotopes:	all	Isotope RI (%):	1.00	Isotope Res:	10000
MSn Iso RI (%):	75.00	MSn Logic Mode:	AND	Max Results:	500

Event#: 1 MS(E+) Ret. Time : 1.093 - 1.280 -> 1.636 Scan# : 165 - 193 -> 247

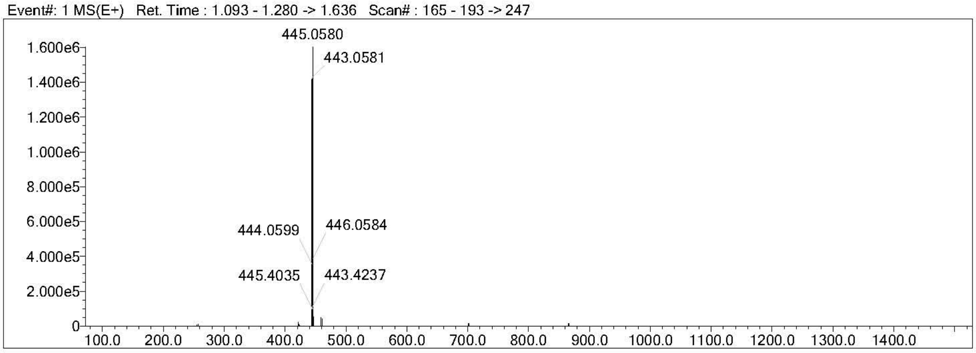

Measured region for 443.0581 m/z

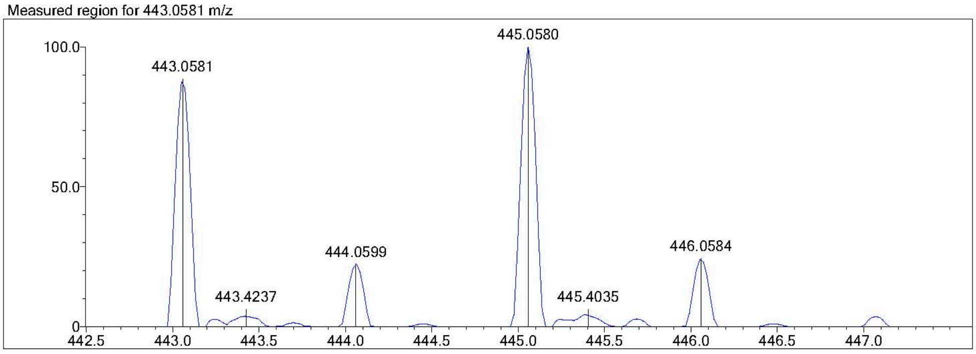

C19 H21 N2 O4 Br [M+Na]+ : Predicted region for 443.0577 m/z

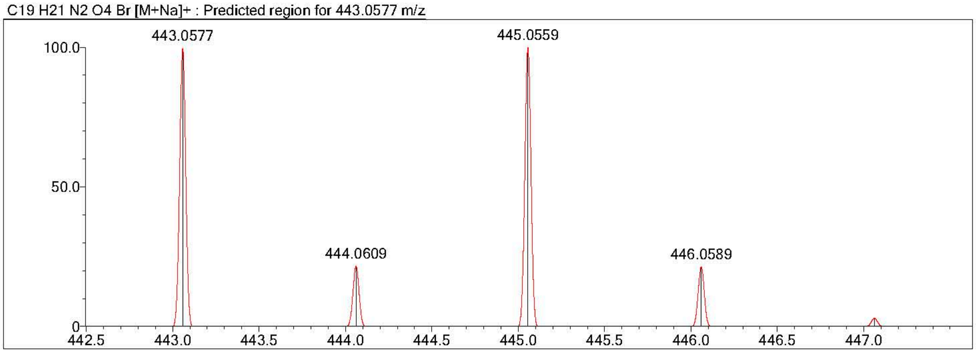

Rank	Score	Formula (M)	Ion	Meas. m/z	Pred. m/z	Df. (mDa)	Df. (ppm)	Iso	DBE
1	72.18	C19 H21 N2 O4 Br	[M+Na]+	443.0581	443.0577	0.4	0.90	72.18	10.0

^{1}H NMR (400 MHz, DMSO-d6) of **8n**

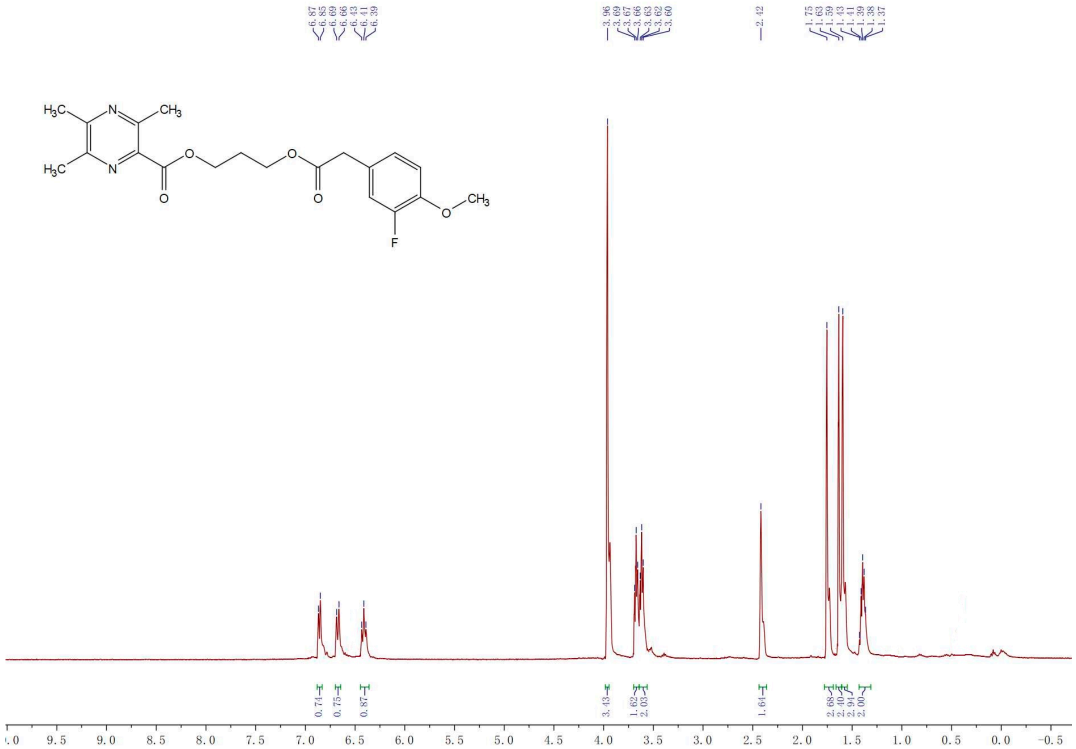

^{13}C NMR (100 MHz, DMSO-d6) of **8n**

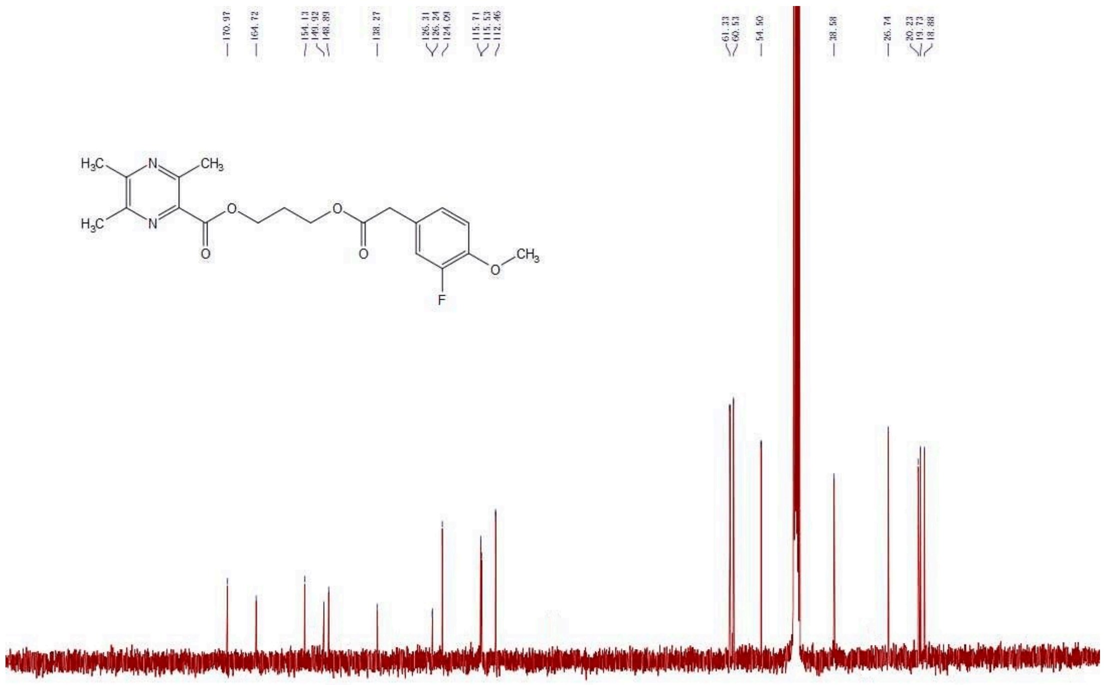

HRMS spectra of **8n**

Data File: I:\20170411-GY\9.lcd

Elmt	Val.	Min	Max	Elmt	Val.	Min	Max	Elmt	Val.	Min	Max	Elmt	Val.	Min	Max	Use Adduct
H	1	0	60	O	2	0	6	P	3	0	0	Cu	2	0	0	H
B	3	0	0	18O	2	0	0	S	2	0	0	Br	1	0	1	Na
C	4	0	50	F	1	0	1	Cl	1	0	0	I	3	0	0	
N	3	0	5	Si	4	0	0	Ni	2	0	0					

Error Margin (ppm):	20	DBE Range:	-2.0 - 1000.0	Electron Ions:	both
HC Ratio:	unlimited	Apply N Rule:	yes	Use MSn Info:	no
Max Isotopes:	all	Isotope RI (%):	1.00	Isotope Res:	10000
MSn Iso RI (%):	75.00	MSn Logic Mode:	AND	Max Results:	500

Event#: 1 MS(E+) Ret. Time : 0.973 - 1.120 -> 1.278 Scan# : 147 - 169 -> 193

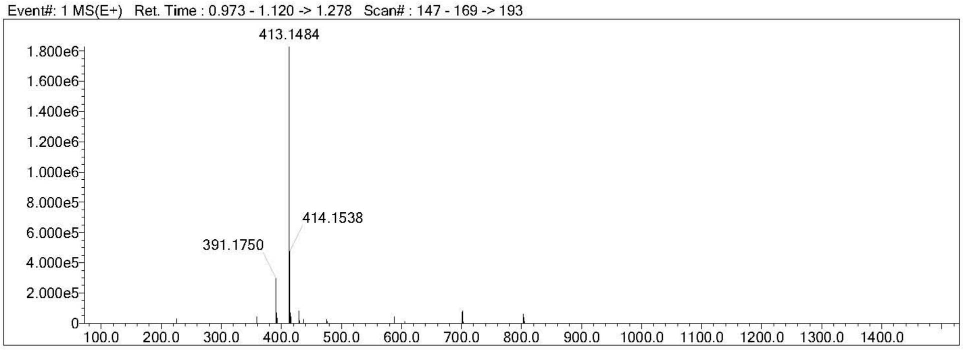

Measured region for 413.1484 m/z

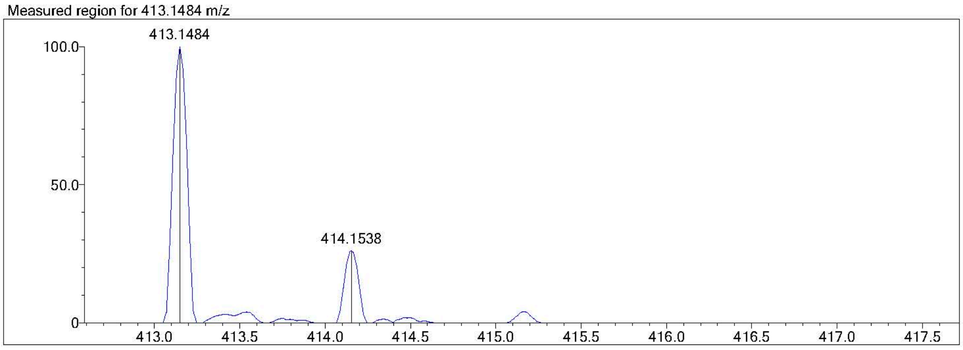

C20 H23 N2 O5 F [M+Na]+ : Predicted region for 413.1483 m/z

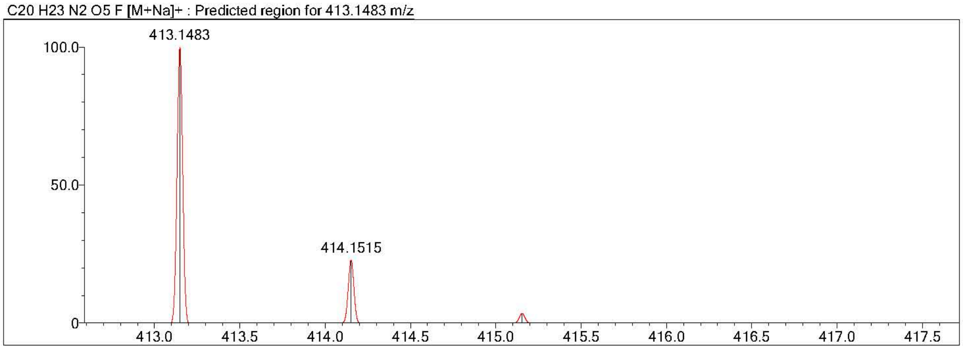

Rank	Score	Formula (M)	Ion	Meas. m/z	Pred. m/z	Df. (mDa)	Df. (ppm)	Iso	DBE
1	87.00	C20 H23 N2 O5 F	[M+Na]+	413.1484	413.1483	0.1	0.24	87.00	10.0

^{1}H NMR (400 MHz, DMSO-d6) of **8o**

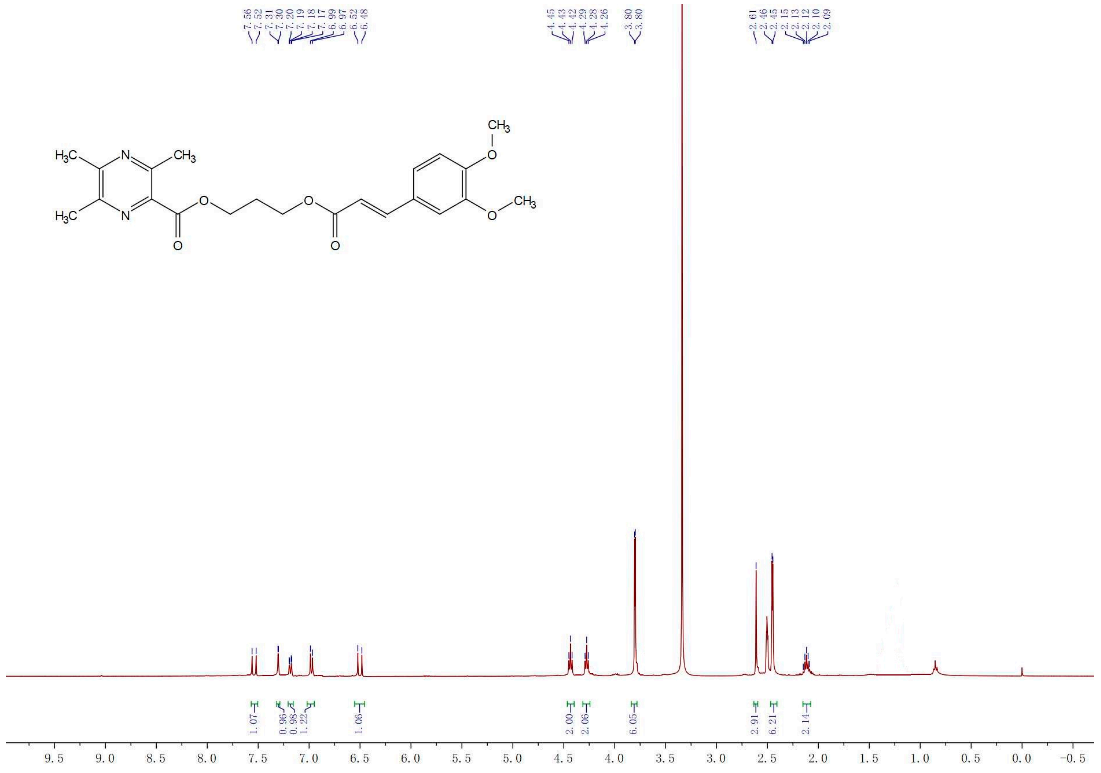

^{13}C NMR (100 MHz, DMSO-d6) of **8o**

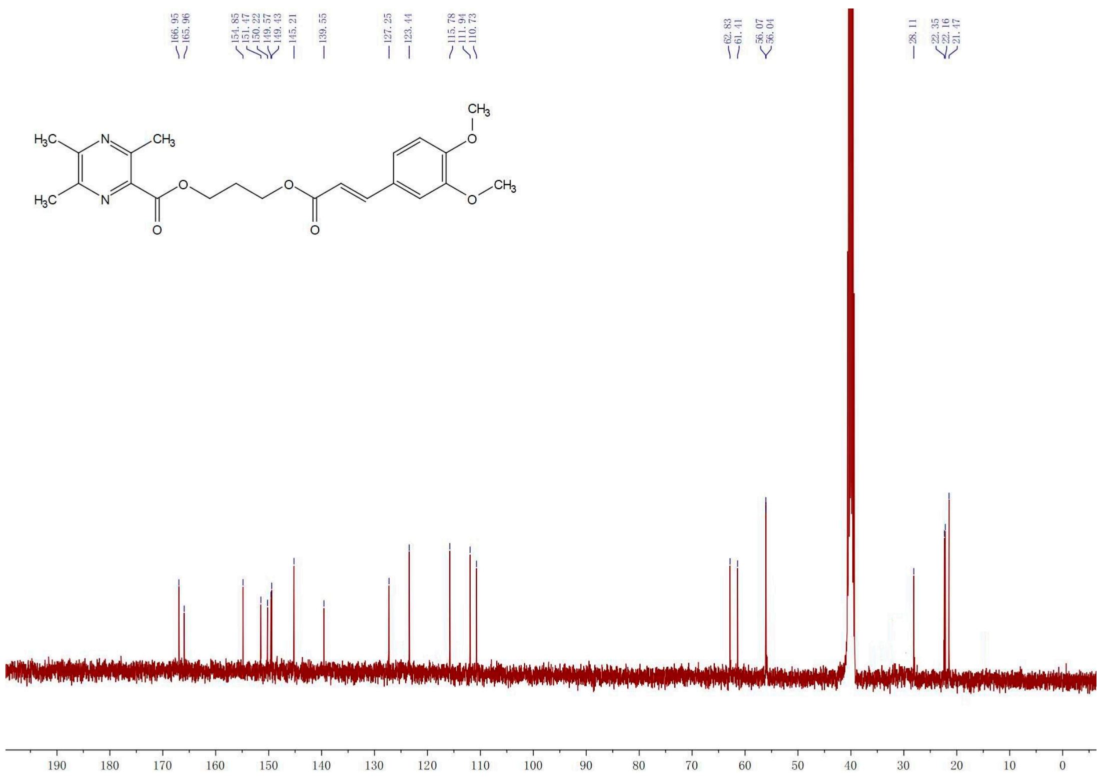

HRMS spectra of **8o**

Formula Predictor Report - 18.lcd

Data File: I:\20170411-GY\18.lcd

Elmt	Val.	Min	Max	Elmt	Val.	Min	Max	Elmt	Val.	Min	Max	Elmt	Val.	Min	Max	Use Adduct
H	1	0	60	O	2	0	7	P	3	0	0	Cu	2	0	0	H
B	3	0	0	18O	2	0	0	S	2	0	0	Br	1	0	1	Na
C	4	0	50	F	1	0	1	Cl	1	0	0	I	3	0	0	
N	3	0	5	Si	4	0	0	Ni	2	0	0					

Error Margin (ppm):	20	DBE Range:	-2.0 - 1000.0	Electron Ions:	both
HC Ratio:	unlimited	Apply N Rule:	yes	Use MSn Info:	no
Max Isotopes:	all	Isotope RI (%):	1.00	Isotope Res:	10000
MSn Iso RI (%):	75.00	MSn Logic Mode:	AND	Max Results:	500

Event#: 1 MS(E+) Ret. Time : 1.187 - 1.227 -> 1.363 Scan# : 179 - 185 -> 205

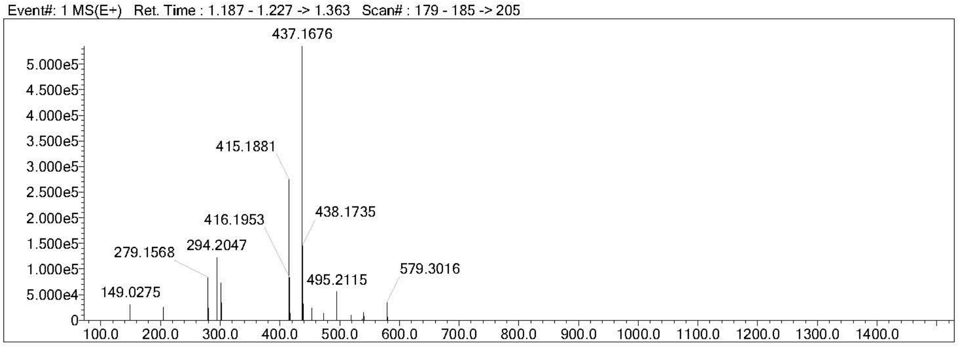

Measured region for 437.1676 m/z

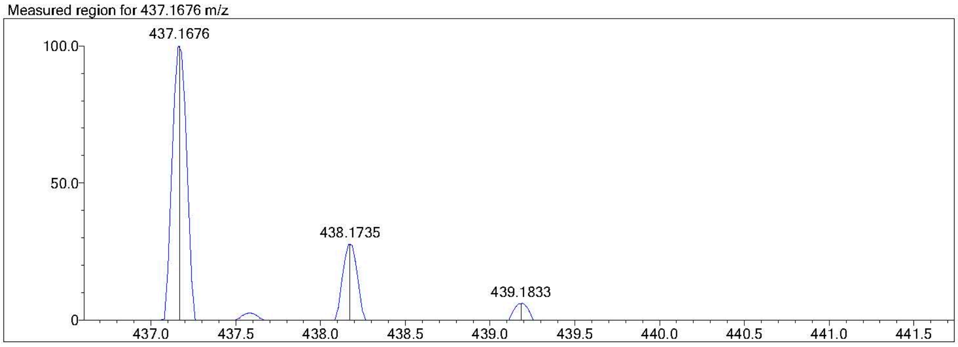

C22 H26 N2 O6 [M+Na]+ : Predicted region for 437.1683 m/z

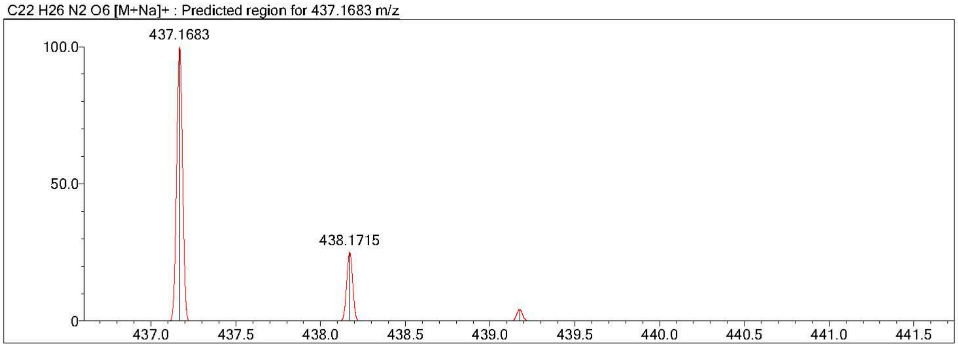

Rank	Score	Formula (M)	Ion	Meas. m/z	Pred. m/z	Df. (mDa)	Df. (ppm)	Iso	DBE
1	80.52	C22 H26 N2 O6	[M+Na]+	437.1676	437.1683	-0.7	-1.60	81.75	11.0

^{1}H NMR (400 MHz, DMSO-d6) of **8p**

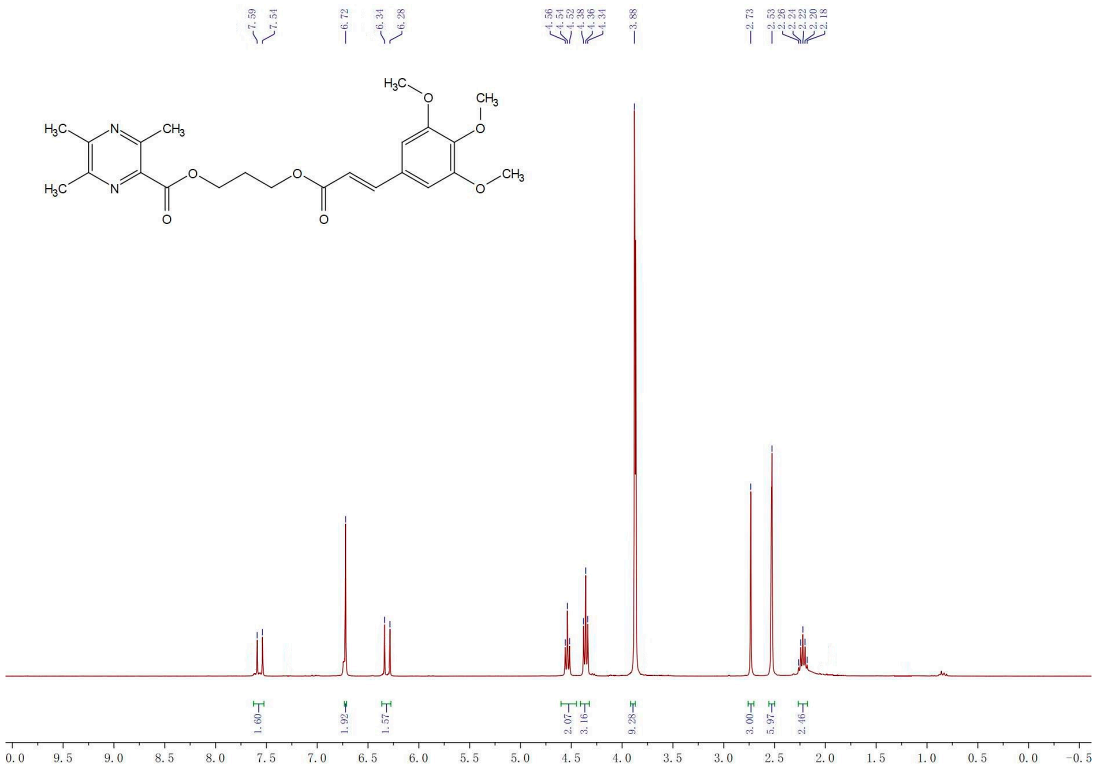

^{13}C NMR (100 MHz, DMSO-d6) of **8p**

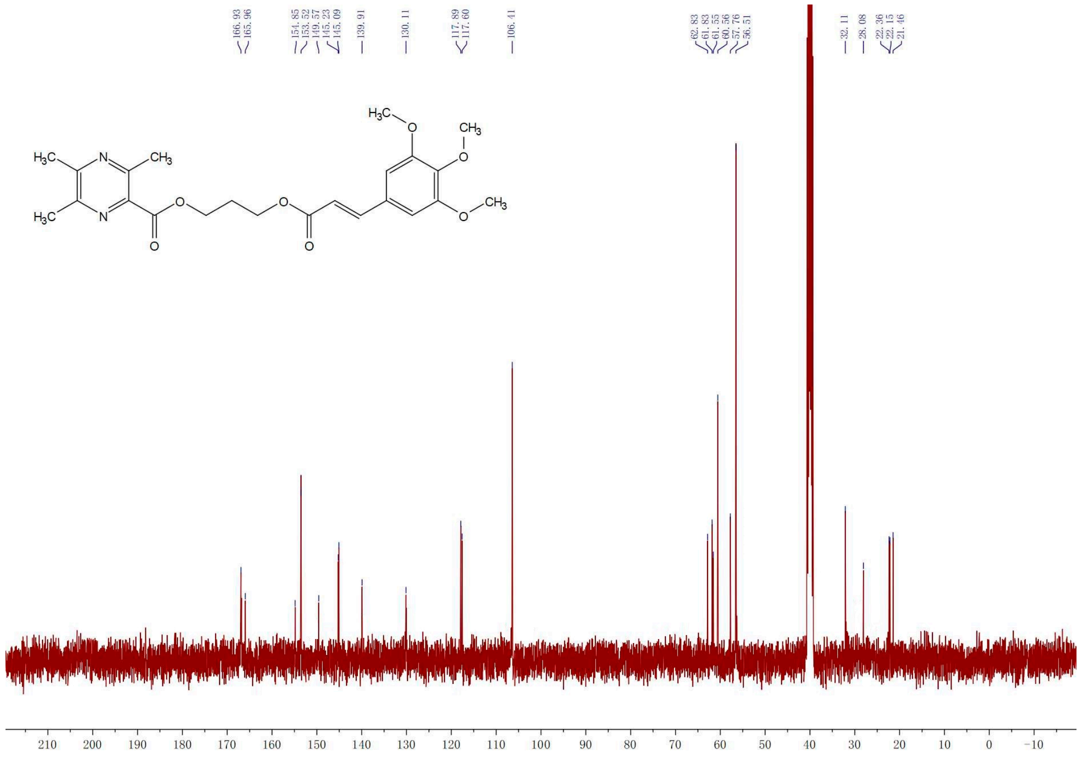

HRMS spectra of **8p**

Formula Predictor Report - 15.lcd   Page 1 of 1

Data File: I:\20170411-GY\15.lcd

Elmt	Val.	Min	Max	Elmt	Val.	Min	Max	Elmt	Val.	Min	Max	Elmt	Val.	Min	Max	Use Adduct
H	1	0	60	O	2	0	7	P	3	0	0	Cu	2	0	0	H
B	3	0	0	18O	2	0	0	S	2	0	0	Br	1	0	1	Na
C	4	0	50	F	1	0	1	Cl	1	0	0	I	3	0	0	
N	3	0	5	Si	4	0	0	Ni	2	0	0					

Error Margin (ppm):	20	DBE Range:	-2.0 - 1000.0	Electron Ions:	both
HC Ratio:	unlimited	Apply N Rule:	yes	Use MSn Info:	no
Max Isotopes:	all	Isotope RI (%):	1.00	Isotope Res:	10000
MSn Iso RI (%):	75.00	MSn Logic Mode:	AND	Max Results:	500

Event#: 1 MS(E+)   Ret. Time : 0.960 - 1.333 -> 1.551   Scan# : 145 - 201 -> 233

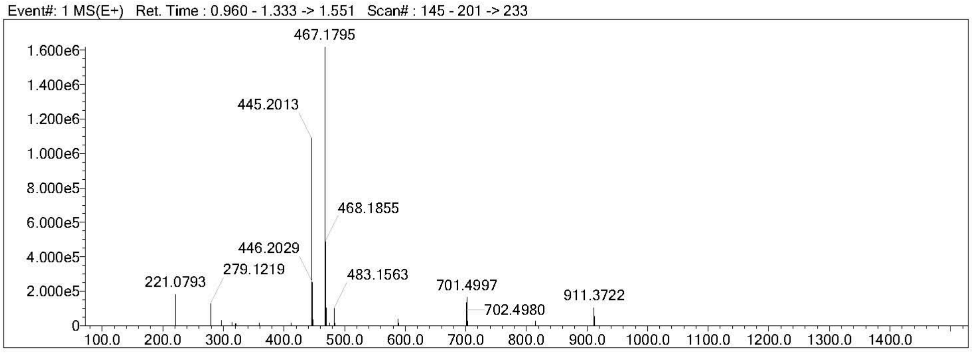

Measured region for 467.1795 m/z

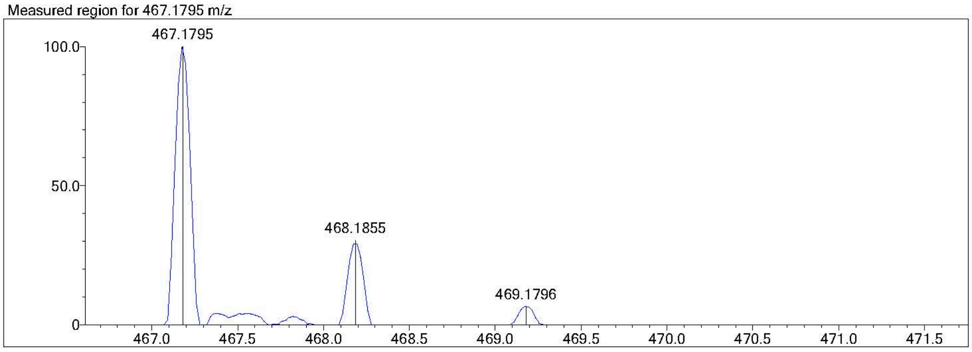

C23 H28 N2 O7 [M+Na]+ : Predicted region for 467.1789 m/z

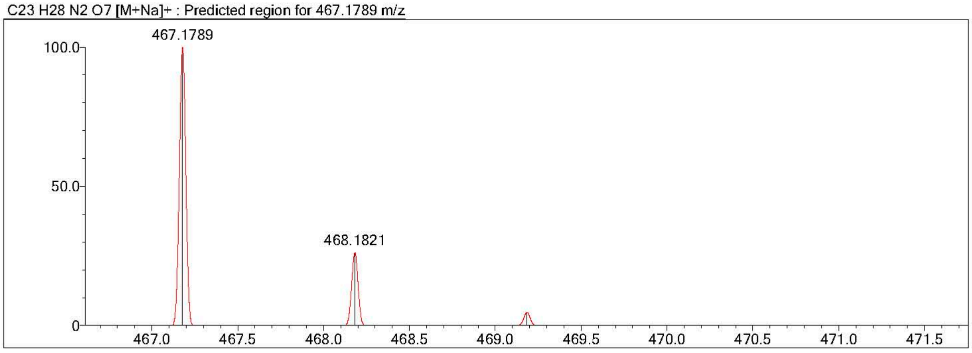

Rank	Score	Formula (M)	Ion	Meas. m/z	Pred. m/z	Df. (mDa)	Df. (ppm)	Iso	DBE
1	84.11	C23 H28 N2 O7	[M+Na]+	467.1795	467.1789	0.6	1.28	84.70	11.0

^{1}H NMR (300 MHz, CDCl3) of **8q**

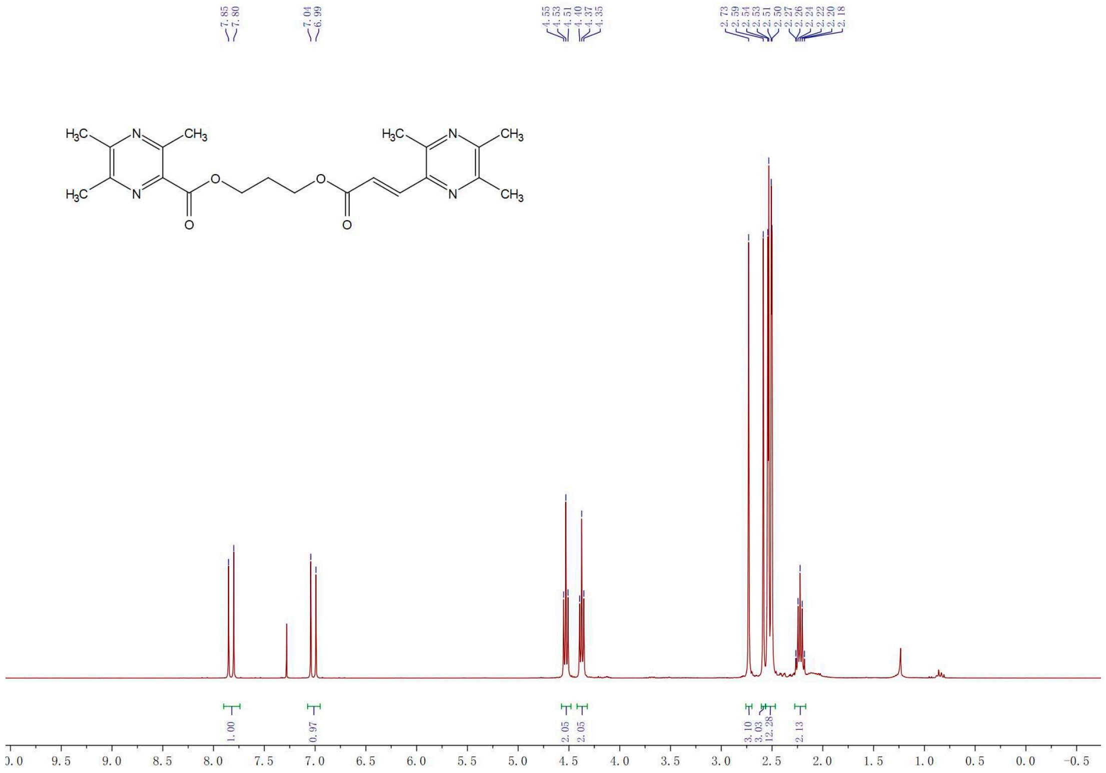

^{13}C NMR (75 MHz, CDCl3) of **8q**

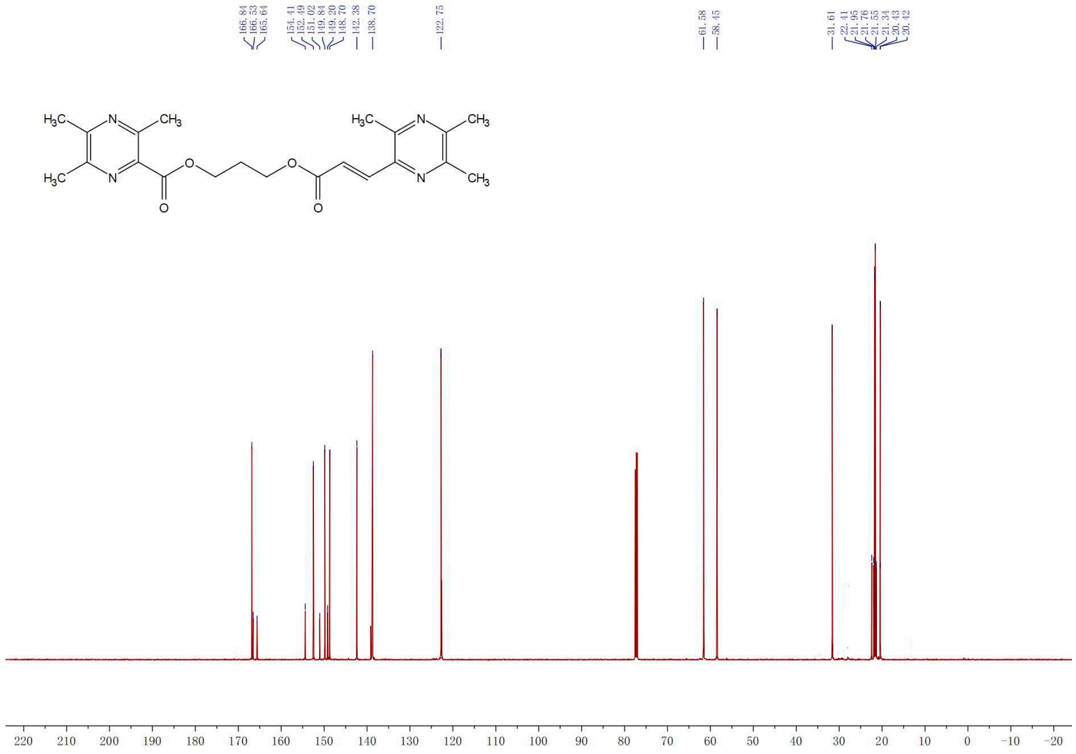

HRMS spectra of **8q**

Formula Predictor Report - 21.lcd | Page 1 of 1

Data File: I:\20170411-GY\21.lcd

Elmt	Val.	Min	Max	Elmt	Val.	Min	Max	Elmt	Val.	Min	Max	Elmt	Val.	Min	Max	Use Adduct
H	1	0	60	O	2	0	7	P	3	0	0	Cu	2	0	0	H
B	3	0	0	18O	2	0	0	S	2	0	0	Br	1	0	1	Na
C	4	0	50	F	1	0	1	Cl	1	0	0	I	3	0	0	
N	3	0	5	Si	4	0	0	Ni	2	0	0					

Error Margin (ppm):	20	DBE Range:	-2.0 - 1000.0	Electron Ions:	both
HC Ratio:	unlimited	Apply N Rule:	yes	Use MSn Info:	no
Max Isotopes:	all	Isotope RI (%):	1.00	Isotope Res:	10000
MSn Iso RI (%):	75.00	MSn Logic Mode:	AND	Max Results:	500

Event#: 1 MS(E+) Ret. Time : 1.067 Scan# : 161

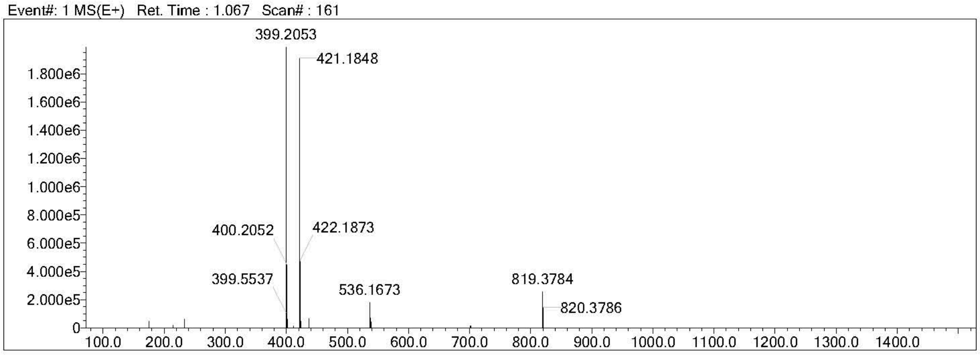

Measured region for 421.1848 m/z

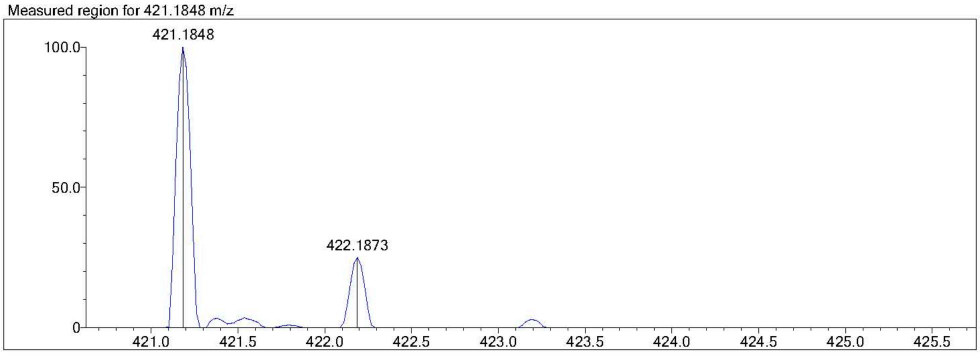

C21 H26 N4 O4 [M+Na]+ : Predicted region for 421.1846 m/z

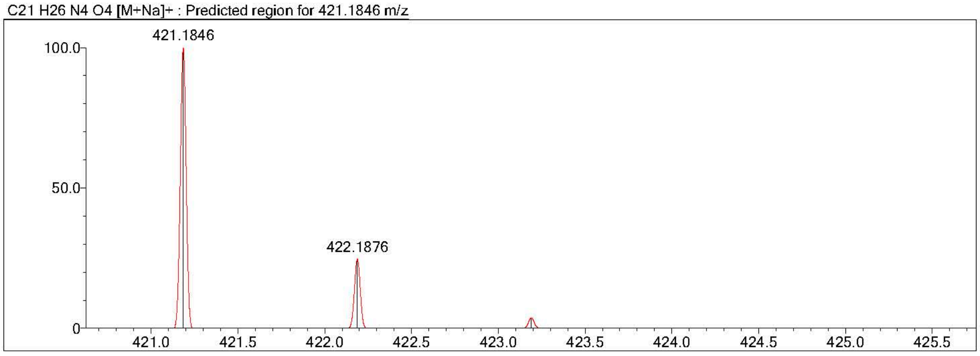

Rank	Score	Formula (M)	Ion	Meas. m/z	Pred. m/z	Df. (mDa)	Df. (ppm)	Iso	DBE
1	91.51	C21 H26 N4 O4	[M+Na]+	421.1848	421.1846	0.2	0.47	91.51	11.0

^{1}H NMR (300 MHz, $CDCl_3$) of **8r**

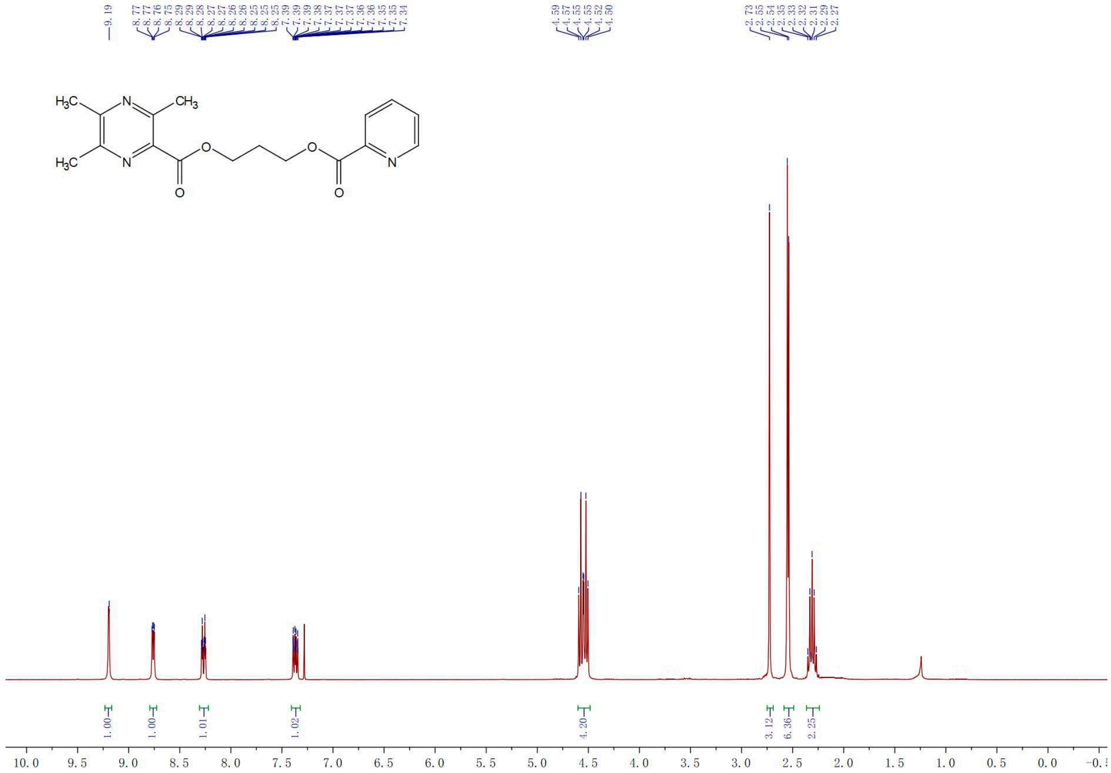

^{13}C NMR (300 MHz, $CDCl_3$) of **8r**

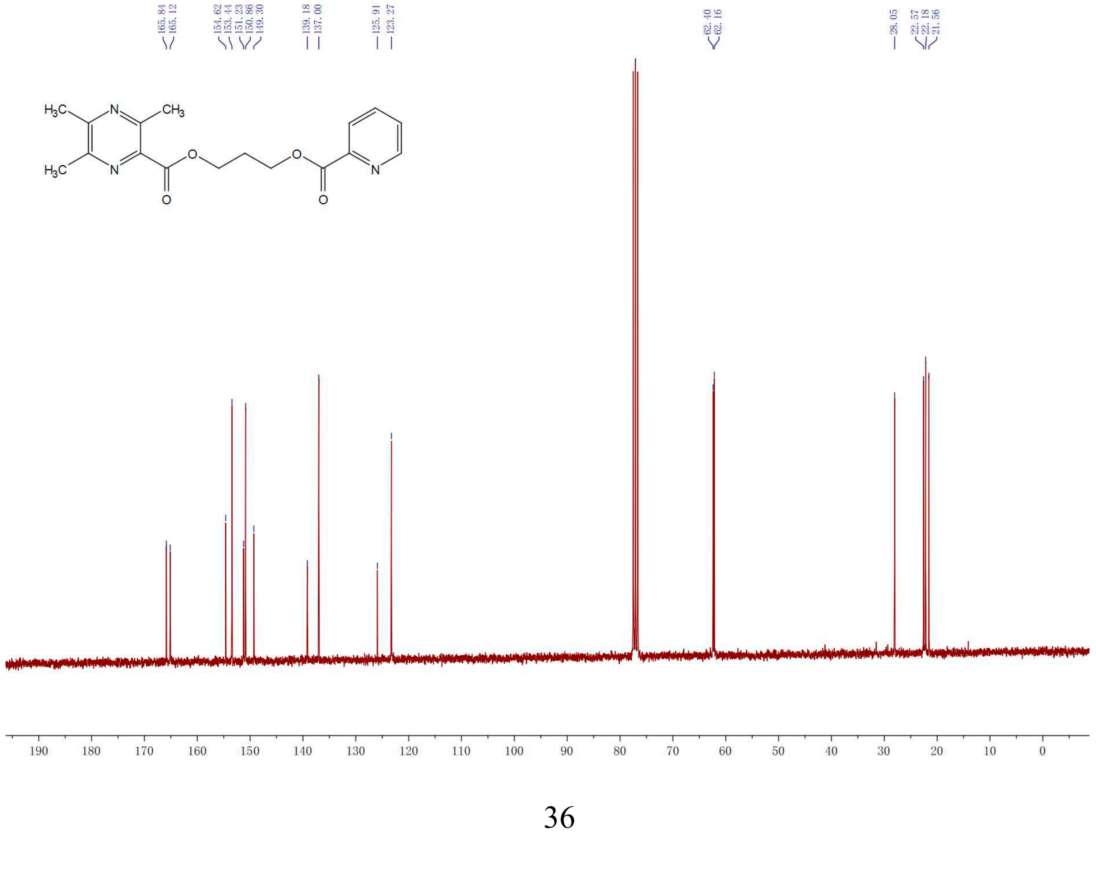

HRMS spectra of **8r**

Formula Predictor Report - 22.lcd

Data File: I:\20170411-GY\22.lcd

Elmt	Val.	Min	Max	Elmt	Val.	Min	Max	Elmt	Val.	Min	Max	Elmt	Val.	Min	Max	Use Adduct
H	1	0	60	O	2	0	7	P	3	0	0	Cu	2	0	0	H
B	3	0	0	18O	2	0	0	S	2	0	0	Br	1	0	1	Na
C	4	0	50	F	1	0	1	Cl	1	0	0	I	3	0	0	
N	3	0	5	Si	4	0	0	Ni	2	0	0					

Error Margin (ppm): 20
HC Ratio: unlimited
Max Isotopes: all
MSn Iso RI (%): 75.00

DBE Range: -2.0 - 1000.0
Apply N Rule: yes
Isotope RI (%): 1.00
MSn Logic Mode: AND

Electron Ions: both
Use MSn Info: no
Isotope Res: 10000
Max Results: 500

Event#: 1 MS(E+) Ret. Time : 0.987 Scan# : 149

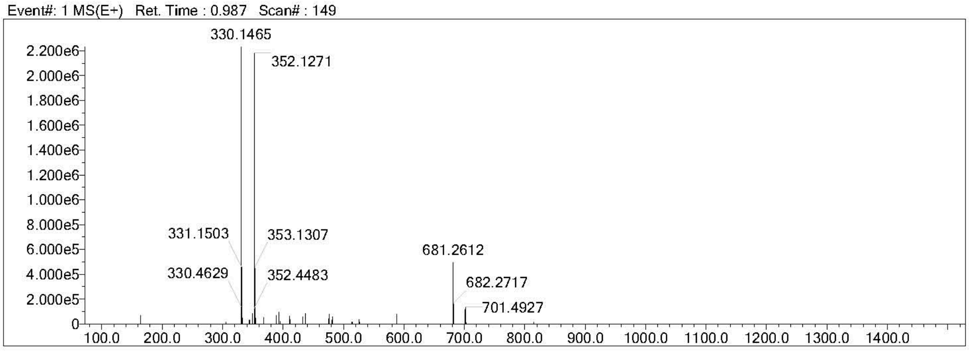

Measured region for 352.1271 m/z

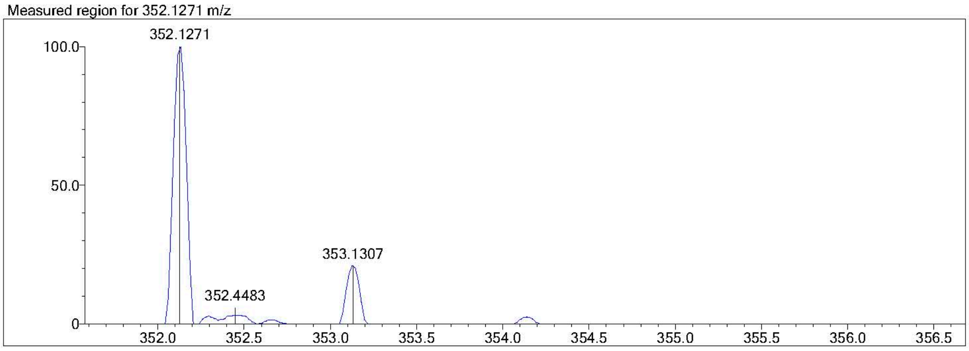

C17 H19 N3 O4 [M+Na]+ : Predicted region for 352.1268 m/z

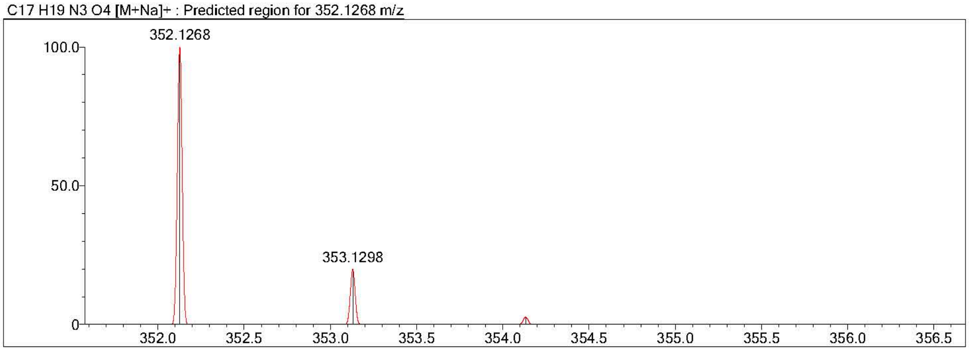

Rank	Score	Formula (M)	Ion	Meas. m/z	Pred. m/z	Df. (mDa)	Df. (ppm)	Iso	DBE
1	86.72	C17 H19 N3 O4	[M+Na]+	352.1271	352.1268	0.3	0.85	86.72	10.0
